# Decarboxylative and dehydrative coupling of dienoic acids and pentadienyl alcohols to form 1,3,6,8-tetraenes

**DOI:** 10.3762/bjoc.13.41

**Published:** 2017-02-28

**Authors:** Ghina’a I Abu Deiab, Mohammed H Al-Huniti, I F Dempsey Hyatt, Emma E Nagy, Kristen E Gettys, Sommayah S Sayed, Christine M Joliat, Paige E Daniel, Rupa M Vummalaneni, Andrew T Morehead, Andrew L Sargent, Mitchell P Croatt

**Affiliations:** 1 Department of Chemistry and Biochemistry, University of North Carolina at Greensboro, Greensboro, NC 27402, USA; 2 Department of Chemistry, East Carolina University, Greenville, NC 27858, USA

**Keywords:** decarboxylation, diene, dienoate, palladium, pentadienyl, tetraene

## Abstract

Dienoic acids and pentadienyl alcohols are coupled in a decarboxylative and dehydrative manner at ambient temperature using Pd(0) catalysis to generate 1,3,6,8-tetraenes. Contrary to related decarboxylative coupling reactions, an anion-stabilizing group is not required adjacent to the carboxyl group. Of mechanistic importance, it appears that both the diene of the acid and the diene of the alcohol are required for this reaction. To further understand this reaction, substitutions at every unique position of both coupling partners was examined and two potential mechanisms are presented.

## Introduction

**Scheme 1:**
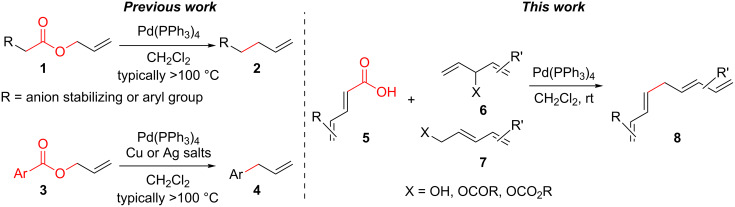
Prior and current decarboxylative couplings.

The construction of sp^2^–sp^3^ carbon–carbon bonds remains a difficult and important problem in organic synthesis. Cross-coupling reactions provide avenues to these otherwise difficult reactions, but often require prefunctionalization of the coupling partners [1–9]. However, recent C–H activation research has enabled the use of further simplified starting materials [10–18]. Another approach to the formation of C–C bonds is through decarboxylative coupling reactions (Scheme 1). This can be arrived in a one-component fashion via the removal of CO_2_ from an ester or in a two-component manner by removal of CO_2_ from a carboxylic acid and coupling this to a substrate with a benzylic or allylic leaving group [19–20].

**Scheme 2:**
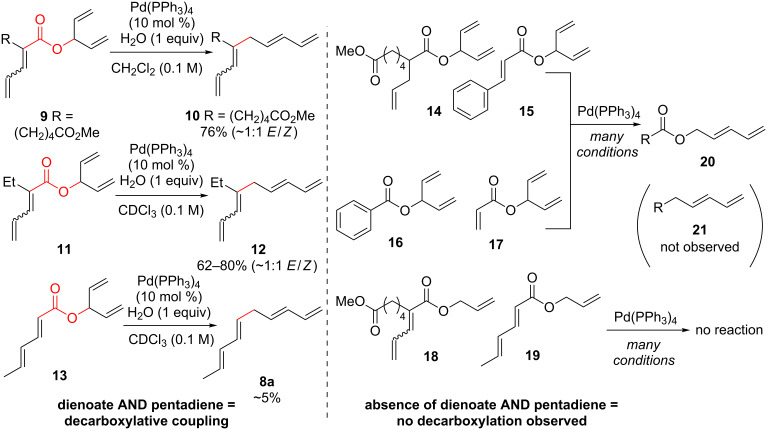
Esters examined in the decarboxylation reaction.

Typical Pd(0)-catalyzed decarboxylative coupling reactions utilize an allylic or benzylic ester with either an anion-stabilizing group adjacent to the carboxyl group (i.e., carbonyl [19,21–22], nitrile [23–25], nitro [26–27], or alkyne [21,28–32], Scheme 1), or use an aryl carboxylate [33–34] which typically requires the assistance of silver or copper(I) salts for the decarboxylative step. It is rare to use a pentadienyl electrophile [35], or to have a diene or simple alkene adjacent to the carboxyl group [20,36–39]. Despite the absence of this type of reactivity, the decarboxylative coupling of a pentadienyl dienoate (**9**; Scheme 2) was desirable enough for our group’s synthesis of clinprost that we attempted the reaction [40–41]. Fortunately, this coupling reaction was successfully employed in our reported nine-step synthesis of clinprost [41]. A structurally related compound (**11**) reacted similarly, however, the sorbate derivative (**13**) was low yielding with the majority of the material only rearranging to the linear ester. In all three of these cases, we never observed the more stable, fully conjugated tetraene. ”Skipped diene” motifs are found in various natural products and there are few methods available to prepare these dienes [42–52]. Skipped tetraene systems have even fewer methods for their synthesis [53–55], which makes the method described herein even more valuable.

It was determined that modifying the dienoate motif yielded only the rearranged product under the reaction conditions, including the dihydro (**14**), cinnamate (**15**), benzoate (**16**), and acrylate (**17**) analogues (Scheme 2). Moreover, allylic dienoates **18** and **19** gave no reaction with Pd(0) catalysis. These results led us to the determination that there was a unique reactivity imbued to the molecule by having both the dienoate and pentadienyl moieties. Herein, are presented more details for this reaction, including the substrate scope for the intermolecular case.

## Results and Discussion

In addition to determining the requisite nature of both the pentadienyl and dienoate groups, it was found that trace amounts of water were required for decarboxylative coupling (Table 1). For example, careful exclusion of water from reagents and solvent and performing the reaction in the glovebox led to formation of rearranged product and no decarboxylative coupling reaction (**22**, Table 1, entry 1). Less than 1 equivalent of water allowed for a slow reaction and incomplete conversion, 1–2 equivalents was optimal with yields around 70% and more water was not beneficial (Table 1, entries 2–8). The use of equimolar amounts of methanol and water as a proton source allowed for decarboxylation to take place but with a low yield (Table 1, entry 9) and the reaction run in TFE as a solvent did not result in any decarboxylation (Table 1, entry 10).

**Table 1:** Optimization of the one-component decarboxylation reaction.^a^

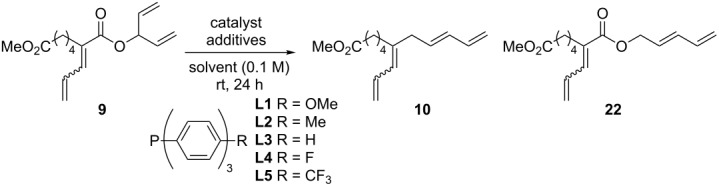				

Entry	Catalyst	Solvent	Additives	Yield of **10** (Yield of **22**)^b^

1	Pd(PPh_3_)_4_	CH_2_Cl_2_	anhydrous	0% (99%)
2	Pd(PPh_3_)_4_	CH_2_Cl_2_	0.5 equiv H_2_O	27%
3	Pd(PPh_3_)_4_	CH_2_Cl_2_	1.1 equiv H_2_O	77%
4	Pd(PPh_3_)_4_	CH_2_Cl_2_	1.3 equiv H_2_O	72%
5	Pd(PPh_3_)_4_	CH_2_Cl_2_	silylated glass, 1 equiv H_2_O	55% (15%)
6	Pd(PPh_3_)_4_	CH_2_Cl_2_	dry glass balls	37% (24%)
7	Pd(PPh_3_)_4_	CH_2_Cl_2_	wet glass balls	51%
8	Pd(PPh_3_)_4_	CH_2_Cl_2_/H_2_O	biphasic	49%
9	Pd(PPh_3_)_4_	CH_2_Cl_2_	1 equiv MeOH, 1 equiv H_2_O	33% (26%)
10	Pd(PPh_3_)_4_	TFE	trace CH_2_Cl_2_	0%
11	Pd_2_(dba)_3_	CH_2_Cl_2_	0 mol % PPh_3_, 1 equiv H_2_O	0%
12	Pd_2_(dba)_3_	CH_2_Cl_2_	10 mol % PPh_3_, 1 equiv H_2_O	64%
13	Pd_2_(dba)_3_	CH_2_Cl_2_	20 mol % PPh_3_, 1 equiv H_2_O	61%
14	Pd_2_(dba)_3_	CH_2_Cl_2_	30 mol % PPh_3_, 1 equiv H_2_O	12%
15	Pd_2_(dba)_3_	CH_2_Cl_2_	10 mol % **L1**, 1 equiv H_2_O	70%
16	Pd_2_(dba)_3_	CH_2_Cl_2_	10 mol % **L2**, 1 equiv H_2_O	70%
17	Pd_2_(dba)_3_	CH_2_Cl_2_	10 mol % **L4**, 1 equiv H_2_O	18%
18	Pd_2_(dba)_3_	CH_2_Cl_2_	10 mol % **L5**, 1 equiv H_2_O	10%
19	Pd(OAc)_2_	CH_2_Cl_2_	1 equiv H_2_O	0%
20	Pd(OAc)_2_	CH_2_Cl_2_	40 mol % PPh_3_, 1 equiv H_2_O	10%
21	none	CH_2_Cl_2_	1 equiv PPh_3_, 1 equiv H_2_O	0%

^a^Reaction Conditions: Pd metal (10 mol %) and the indicated solvent and additives for 24 hours. ^b^Isolated yields.

In addition to the requirement for water, it was determined that phosphine ligands were necessary (Table 1, entry 11), either as ligands or as participants in the reaction as discussed later. The typical catalyst used, Pd(PPh_3_)_4_, worked well, however, it was found that a more ideal ratio of palladium metal to ligand was 1:1 or 1:2, with greater amounts of triphenylphosphine lowering the reaction yield when using the Pd_2_dba_3_ catalyst (Table 1, entries 12–14). It was determined that reactions performed in the presence of electron-rich ligands had both quicker kinetics and more efficient yields compared to electron-deficient ligands (Table 1, entries 15–18 and Supporting Information File 1 for kinetic information). Although not as efficient, it was found that a palladium(II) catalyst functioned in this reaction, presumably functioning as a pre-catalyst and being reduced in situ to the palladium(0) catalyst (Table 1, entries 19 and 20). As a control reaction, it was found that no reaction occurred in the absence of palladium catalyst (Table 1, entry 21).

As shown earlier, bis-allylic sorbate **13** (Scheme 2) was found to be low yielding for the decarboxylative coupling reaction. Reactions of sorbate **13** monitored by ^1^H NMR showed nearly quantitative isomerization of the bis-allylic group into a linear pentadienyl system. Increasing the reaction time did not result in greater conversion to tetraene **8a**, which indicates that the products may be competitively ligating and poisoning the Pd(0) catalyst (see Supporting Information File 1 for additional evidence of product inhibition). The isomerization reaction to form **22** was presumably occurring via ionization of the allylic system using Pd(0), followed by recombination of the carboxylate at the terminal position of the pentadienyl system. Based on these data, we hypothesized that a two-component reaction using a dienoic acid and bis-allylic acetate might be possible, however, the presence of both water and a carboxylic acid would increase the possibility for isomerization of the 1,3,6,8-tetraenes into the fully conjugated 1,3,5,7-tetraenes, or possibly polymerization.

**Scheme 3:**
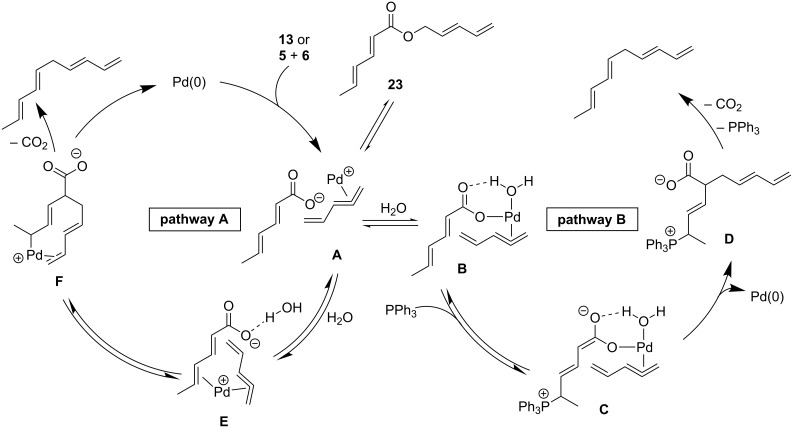
Possible mechanistic pathways.

Despite the low yield for decarboxylation with sorbate **13**, the initial attempt used inexpensive sorbic acid as the dienoic acid. Gratifyingly, this reaction was successful and it was again determined that no isomerization to the fully conjugated system was observed (Table 2, entry 1). Other bis-allylic leaving groups were studied and, unexpectedly, it was determined that divinylcarbinol was superior (Table 2, entries 1–6). In fact, the better leaving groups were either slow or ineffective. This could be due to the less basic leaving groups not sufficiently deprotonating sorbic acid, which may be required for this reaction as is discussed mechanistically later (Scheme 3). Similar to the single component reaction, more than two equivalents of phosphine, relative to palladium metal, was detrimental (compare entries 12–14 of Table 1 with entries 6–8 of Table 2), however, the reaction was successful using Pd(PPh_3_)_4_ (Table 1, entry 9).

**Table 2:** Optimization of the two-component decarboxylation reaction.^a^

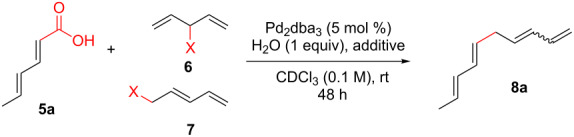			

Entry	Pentadienyl group	Additive	Yield^b^

1	**6a**, X = OAc	PPh_3_ (20 mol %)	12%
2	**6b**, X = OCO_2_Me	PPh_3_ (20 mol %)	35%
3	**6c**, X = OBz	PPh_3_ (20 mol %)	11%
4	**6d**, X = O_2_C(4-CF_3_Ph)	PPh_3_ (20 mol %)	6%
5	**7a**, X = Br	PPh_3_ (20 mol %)	0%
6	**6e**, X = OH	PPh_3_ (20 mol %)	40%
7	**6e**, X = OH	PPh_3_ (10 mol %)	18%
8	**6e**, X = OH	PPh_3_ (30 mol %)	24%
9^c^	**6e**, X = OH	NA	28%

^a^Reaction conditions: Sorbic acid (**5a**, 1 equiv), pentadienyl group (**6** or **7**, 1 equiv), Pd_2_(dba)_3_·CHCl_3_ (5 mol %) unless indicated otherwise, H_2_O (1 equiv), in CDCl_3_ for 48 hours. ^b^NMR yields. ^c^Pd(PPh_3_)_4_ (10 mol %).

To further understand this interesting decarboxylative coupling reaction, a handful of different pentadienyl electrophiles and dienoic acids were examined (Table 3). Typically, the pentadienyl alcohol was used; however, in some cases the acetate was superior. It was found that both a methyl or phenyl substituent on the alcohol derivative would result in branched product **8b** or **8d** as a major product with a product ratio of 6:1 or 4:1, respectively (Table 3, entries 2–4). The yields for these reactions were low, but the remaining material was typically starting material and the ester where the acid and alcohol are coupled together. There was no effect on the yields upon leaving the reactions longer than 48 hours and it was found that the addition of tetraene product inhibited the reaction (see Supporting Information File 1 for details). With these highly unsubstituted tetraene products, it is hypothesized that the product may be sequestering the palladium catalyst. Two cyclic dienyl acetates were also studied (Table 3, entries 5 and 6) and they yielded tetraenes **8f** and **8g**. The dienes of entries 5 and 6 could have formed additional isomers by coupling to the other end of the pentadienyl group, but only one regioisomer was observed.

**Table 3:** Substrate scope for the two-component decarboxylation reaction.^a^

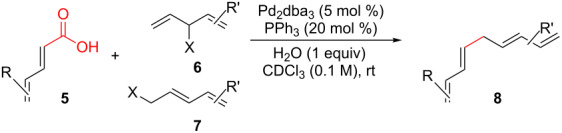			

Entry	Dienoic acid	Pentadienyl group	Yield (product)^b^

1	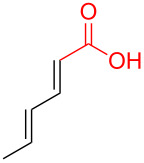 **5a**	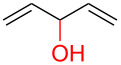 **6e**	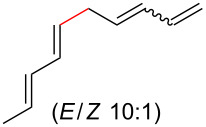 40%^b^ (**8a**)
2	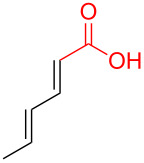 **5a**	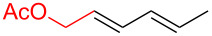 **7b**	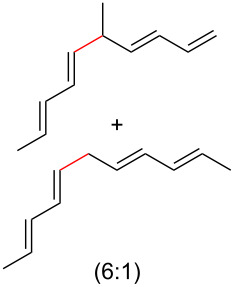 8%^c^ (**8b**/**8c**)
3	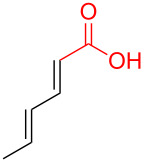 **5a**	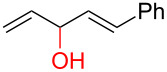 **6f**	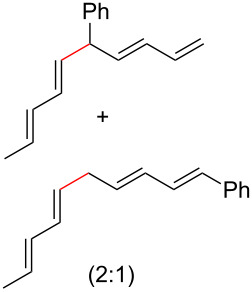 21%^b^, 17%^c^ (**8d**/**8e**)
4	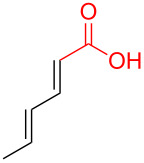 **5a**	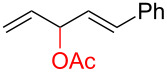 **6g**	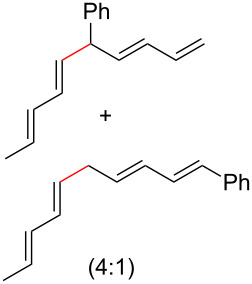 13%^b^, 6%^c^ (**8d**/**8e**)
5	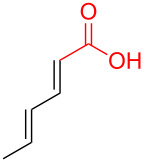 **5a**	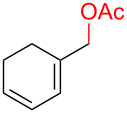 **7c**	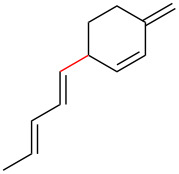 16%^c^ (**8f**)
6	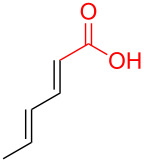 **5a**	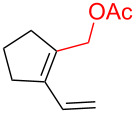 **7d**	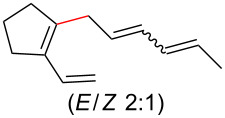 18%^c^ (**8g**)
7	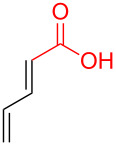 **5b**	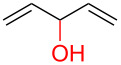 **6e**	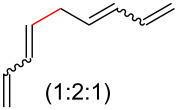 14%^b^ (**8h**/**8i**/**8j**)
8	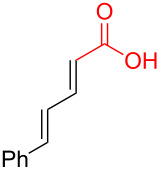 **5c**	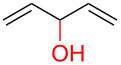 **6e**	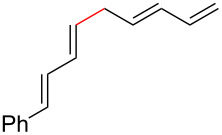 24%^c^ (**8k**)
9	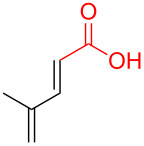 **5d**	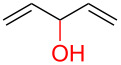 **6e**	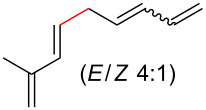 36%^b^ (**8l**)
10	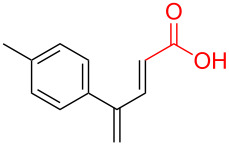 **5e**	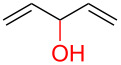 **6e**	decomposition
11	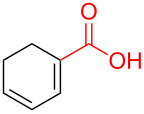 **5f**	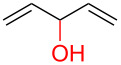 **6e**	decomposition
12	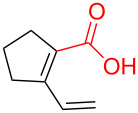 **5g**	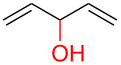 **6e**	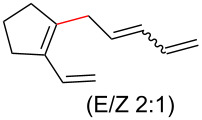 74%^c^ (**8m**)

^a^Reaction conditions: Dienoic acid (**5**, 1 equiv), pentadienyl group (**6** or **7**, 1 equiv), H_2_O (1 equiv), Pd_2_(dba)_3_·CHCl_3_ (5 mol %), PPh_3_ (20 mol %), in CDCl_3_ for 48 h. ^b^NMR yields due to volatility of product. ^c^Isolated yields.

With respect to the dienoic acid, it was determined that the unsubstituted compound, pentadienoic acid, underwent decarboxylative coupling, although as a mixture of *E*/*E*, *E*/*Z*, and *Z*/*Z* isomers (Table 3, entry 7). Alkyl and aryl substituents were possible on the dienoate with the exception of an aryl group at the gamma position (Table 3, entries 8–10). Two cyclic dienoic acids were synthesized [56–57] and while the cyclohexadienoic acid did not decarboxylate (Table 3, entry 11), the vinylcyclopentenoic acid had a good yield of a complex tetraene (Table 3, entry 12).

Based on the information obtained during optimization and screening of compounds, two potential mechanisms are proposed (Scheme 3). Both options allow for the one (**13**) or two (**5** and **6**) component process to be used while also allowing for the reversible formation of linear ester **23**. Pathway B involves a Morita–Baylis–Hillman type process. The role of water would be to hydrogen bond to the carboxylate to make the system more electrophilic (**B**). This would accelerate the addition of the phosphine to generate zwitterion **C** [58]. Preliminary modeling for this ion indicates that both the electrophilic terminal vinyl group of the pentadienyl ligand and the nucleophilic α-carbon are in close proximity to one another. Formation of the carbon–carbon bond would then regenerate the Pd(0) catalyst and phosphonium carboxylate **D**. Decarboxylative elimination of the phosphine results in formation the 1,3,6,8-tetraene. It is proposed for pathway B that the dienoate is required so that the α-carbon is not blocked by the bulky phosphine group since it can add in a 1,6- or 1,4-manner, both reversibly.

**Figure 1:**
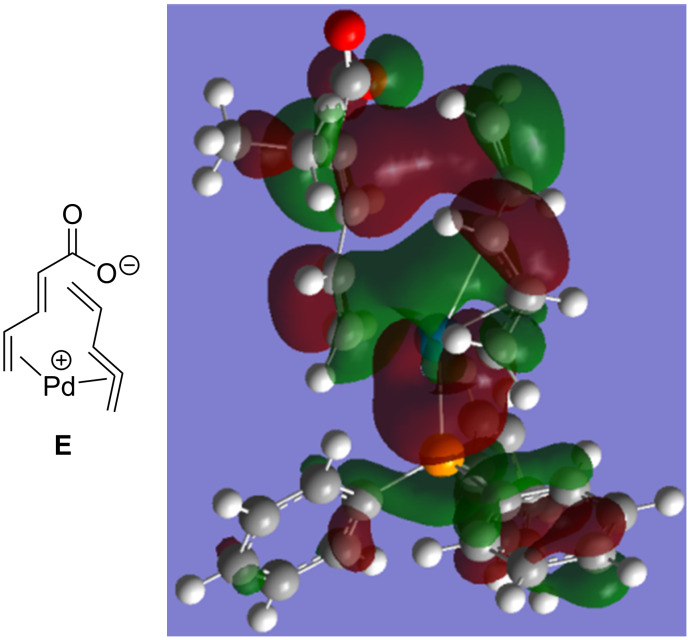
Calculated HOMO of transition state between **E** and **F**.

Alternatively, pathway A has the palladium catalyst coordinate to one of the alkenes of the dienoate instead of the carboxylate (**E**). It is proposed that a water cluster around the carboxylate would enable this process by hydrogen bonding to the carboxylate. The conversion of **E** to **F** would form the C–C bond by having the palladium catalyst convert from one type of η^3^-allyl and π-complex (**E**) to a different allyl/π-complex (**F**). Finally, decarboxylative reduction of the palladium would release the product while regenerating the catalyst. Preliminary computational calculations using NEB [59] support pathway A and the HOMO of the transition state between **E** and **F** (Figure 1) calculated using the Gaussian 09 implementation of DFT with a B3LYP functional, 6-31g* basis, and polarized continuum model of solvation for DCM, shows close proximity of two in-phase orbitals for the requisite C–C bond, whereas removal of any one of the alkenes from this structure would lead to anti-bonding relationships to bond to the alpha carbon.

## Conclusion

In summary, we present information that is of value to advancing the area of metal-catalyzed decarboxylative coupling reactions, specifically those of pentadienyl dienoates that do not require an anion-stabilizing group, are run at ambient temperature, and can utilize the more accessible alcohol for a leaving group. This reaction was advanced to be possible in a two-component fashion, allowing for the conversion of dienoic acids and pentadienyl alcohols into 1,3,6,8-tetraenes with the only stoichiometric byproducts being water and carbon dioxide. These reactions currently require a diene motif with each coupling partner, but the product maintains the independent reactivity opportunities of these isolated dienes as opposed to forming the fully conjugated 1,3,5,7-tetraene. A variety of substrates were explored where each of the unique positions on the coupling partners was modified and two different mechanistic pathways are presented. A more in-depth mechanistic analysis to improve the yields and to explore other reactivity possibilities based on this process are currently being studied and will be published in due time.

## Supporting Information

File 1 Experimental procedures and analytical data for all substrates and products, product inhibition study, computational calculation information, and relevant energies and Cartesian coordinates.
