# Supplementary material for: Decarboxylative and dehydrative coupling of dienoic acids and pentadienyl alcohols to form 1,3,6,8-tetraenes
Source: Beilstein J Org Chem. 2017 Feb 28;13:384–92. doi: 10.3762/bjoc.13.41 (PMC5355911; doi:10.3762/bjoc.13.41)
Supplement: File 1 — Experimental procedures and analytical data for all substrates and products, product inhibition study, computational calculation information, and relevant energies and Cartesian coordinates. [file Beilstein_J_Org_Chem-13-384-s001.pdf]

Supporting Information

for

Decarboxylative and dehydrative coupling of dienoic acids and pentadienyl alcohols to form 1,3,6,8-tetraenes

Ghina'a I. Abu Deiab[‡,1], Mohammed H. Al-Huniti[1,‡], I. F. Dempsey Hyatt[§1],Emma E. Nagy[1], Kristen E. Gettys[1], Sommayah S. Sayed[1], Christine M. Joliat[1], Paige E. Daniel[1], Rupa M. Vummalaneni[1], Andrew T. Morehead, Jr.[2], Andrew L. Sargent[2] and Mitchell P. Croatt*[,1]

Address: [1] Department of Chemistry and Biochemistry, University of North Carolina at Greensboro, Greensboro, NC 27402, USA and [2] Department of Chemistry, East Carolina University, Greenville, NC 27858, USA

Email: Mitchell P. Croatt - mpcroatt@uncg.edu

*Corresponding author

[‡]Contributed equally

[§]Current Address: Department of Chemistry, Adelphi University, Garden City, NY 11530, USA

Experimental procedures and analytical data for all substrates and products, product inhibition study, computational calculation information, and relevant energies and Cartesian coordinates

Table of contents for the supporting information

General information – page S2

Kinetic data for different phosphine ligands – page S3

Experimental procedures and characterization of pentadienyl dienoates – page S4

Experimental procedures and characterization of dienoic acids – page S6

Experimental procedures and characterization of pentadienyl groups – page S13

Experimental procedures for single component decarboxylative coupling – page S30

Experimental procedures for two component decarboxylative coupling – page S32

Product inhibition experiment – page S49

Computational methods – page S49

Relevant Cartesian coordinates – page S50

References – page S62

General information

All anhydrous reactions were performed in oven dried glassware under a nitrogen atmosphere. Unless otherwise noted, all solvents and reagents were obtained from commercial sources and used without further purification. NMR yields were obtained by using dimethyl terphthalate as an internal standard in a $CDCl_3$ solution. Chromatographic purification was performed using silica gel (60 Å, 32–63 μm). NMR spectra were recorded in $CDCl_3$ using a JEOL ECA 400 spectrometer (400 MHz for ^1H, 100 MHz for ^{13}C, and 376.5 MHz for ^{19}F) and JEOL ECA spectrometer (500 MHz for ^1H, 125 MHz for ^{13}C, and 470 MHz for ^{19}F). Coupling constants, *J*, are reported in hertz (Hz) and multiplicities are listed as singlet (s), doublet (d), triplet (t), quartet (q), quintet (quint), doublet of doublets (dd), triplet of triplets (tt), multiplet (m), etc. High Resolution Mass Spectra were acquired on a Thermo Fisher Scientific LTQ Orbitrap XL MS system. Low Resolution Mass Spectrometry was accomplished using Gas Chromatography on a Shimadzu GC2010-QP2010S instrument.

Kinetic data for different phosphine ligands

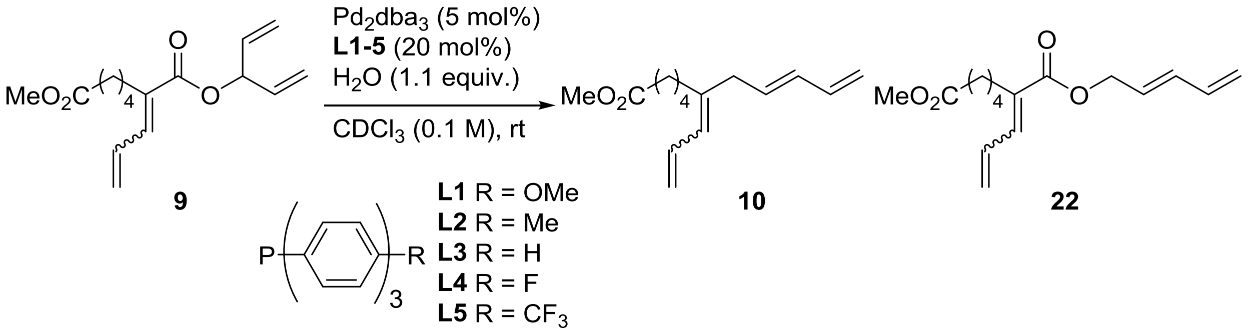

To a prepared solution of $Pd_2dba_3 \cdot CHCl_3$ (5 mol %) and designated ligand (see above and Chart; 20 mol %) in $CDCl_3$ (0.1 M) was added pentadienyl dienoate **9** (20 mg, 0.072 mmol) at ambient temperature. ^{1}H NMR spectra were obtained upon dissolution of the reagents and subsequent ^{1}H NMR spectra were obtained every 90 minutes. The formation of the product **10** was monitored and plotted versus time in Chart S1.

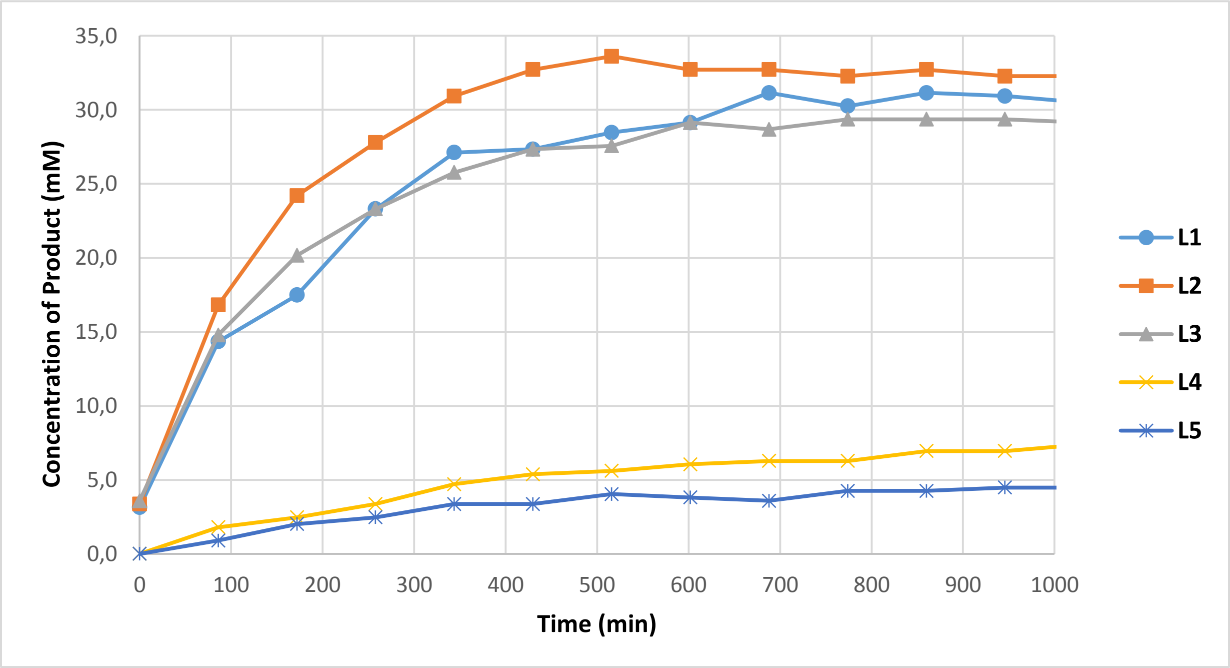

Chart S1. Formation of product **10** using different phosphine ligands

Experimental procedures and characterization of pentadienyl dienoates

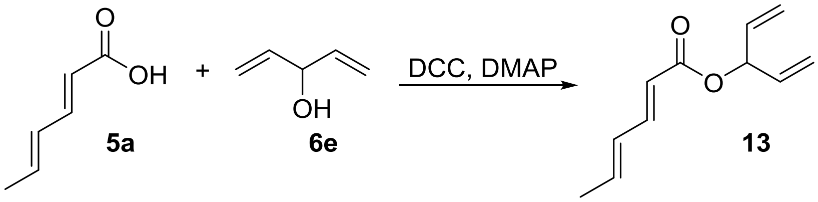

Penta-1,4-dien-3-yl (2*E*,4*E*)-hexa-2,4-dienoate (13)

To an ice-cooled solution of DCC (0.83 g, 4.0 mmol) in THF (15 mL) were sequentially added sorbic acid (0.50 g, 2.8 mmol), 1,4-pentadien-3-ol (**6e**, 0.34 mL, 3.4 mmol) and DMAP (70 mg, 0.57 mmol). The reaction was allowed to warm to room temperature for 18 hours before it was filtered through Celite® and washed with hexane. The filtrate was concentrated and purified via silica gel chromatography (95:5, hexanes/EtOAc) to yield ester **13** (0.42 g, 82%) as a colorless oil. R_f = 0.78 (88:12, hexanes/EtOAc).

^{1}H NMR (500 MHz, $CDCl_3$) δ 7.31 – 7.26 (m, 1H), 6.23 – 6.11 (m, 1H), 5.91 – 5.83 (m, 3H), 5.80 – 5.77 (m, 2H), 5.30 (dt, J_d = 17.0 Hz, J_t = 1.5 Hz, 2H), 5.22 (dt, J_d = 10.0 Hz, J_t = 1.0 Hz, 2H), 1.86 (d, J = 6.0 Hz, 3H) ppm.

^{13}C NMR (100 MHz, $CDCl_3$) δ 166.3, 145.5, 139.7, 135.3 (2C), 129.8, 118.9, 117.4 (2C), 74.8, 18.8 ppm.

HRMS (APCI): calcd. for $[C_{11}H_{14}O_2+H]^+$: 179.1067, found: 179.1066.

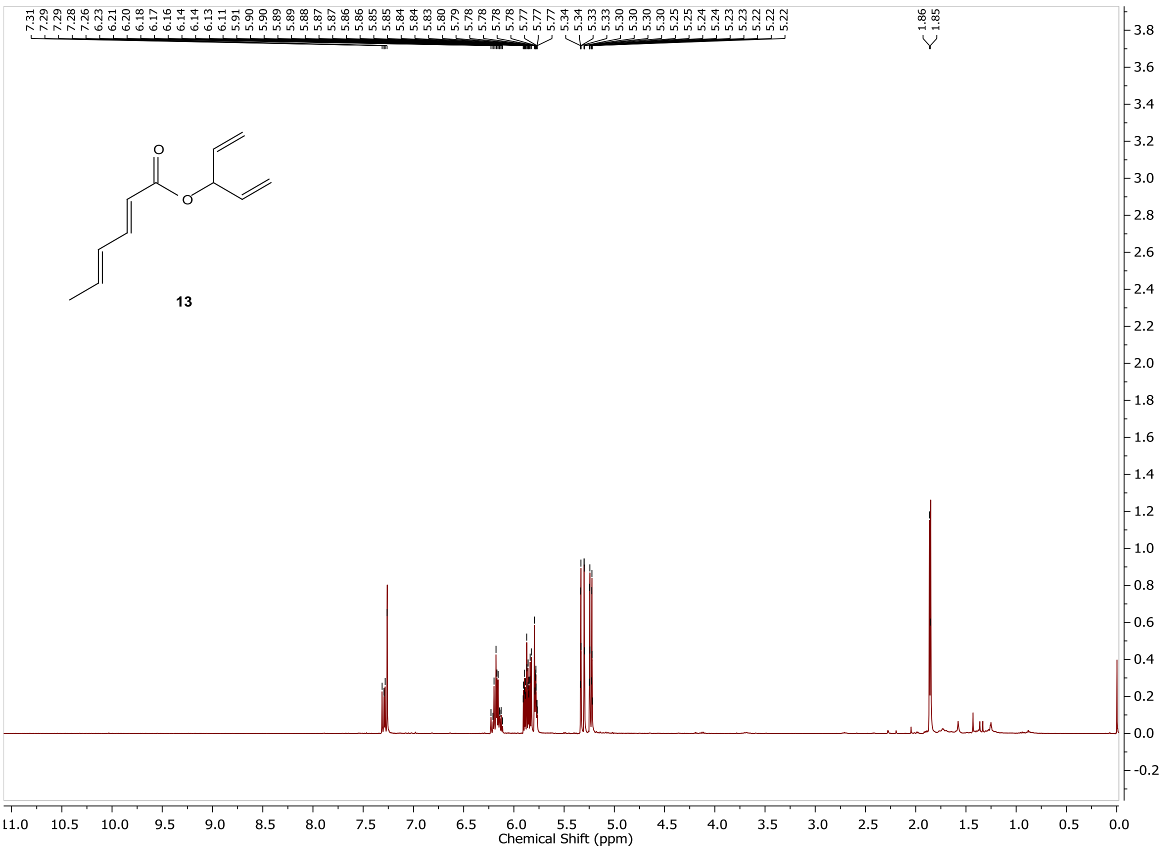
7.31 7.29 7.29 7.28 7.26 6.23 6.21 6.20 6.18 6.17 6.16 6.14 6.14 6.13 6.11 5.91 5.90 5.90 5.89 5.89 5.88 5.87 5.87 5.86 5.86 5.85 5.85 5.84 5.84 5.83 5.80 5.79 5.78 5.78 5.78 5.77 5.77 5.77 5.34 5.34 5.33 5.33 5.30 5.30 5.30 5.25 5.25 5.24 5.24 5.23 5.23 5.22 5.22 5.22
1.86 1.85
O
O
13
Chemical Shift (ppm)

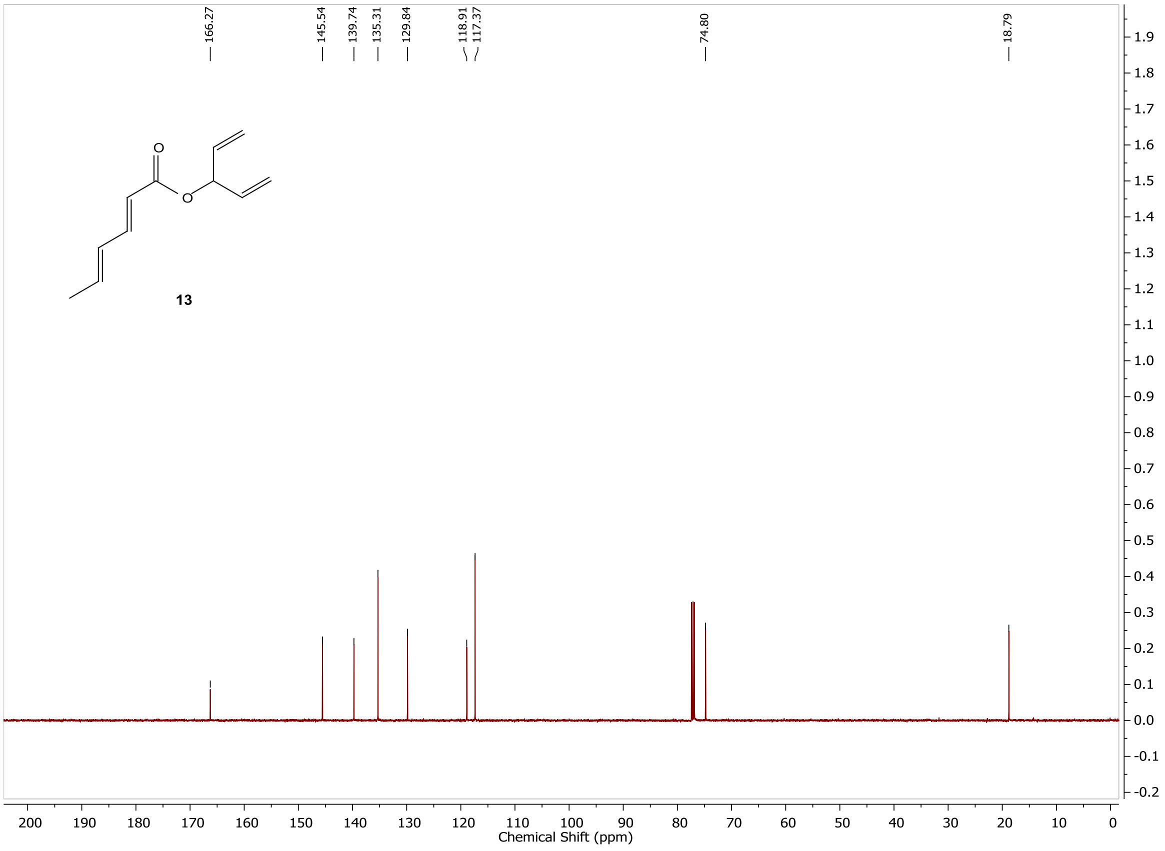
166.27
145.54
139.74
135.31
129.84
118.91
117.37
74.80
18.79
O
O
13
Chemical Shift (ppm)

Experimental procedures and characterization of dienoic acids

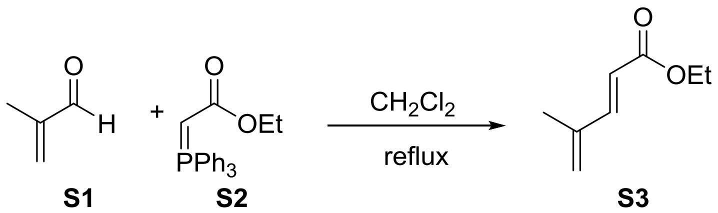

(*E*)-Ethyl 4-methylpenta-2,4-dienoate (S3)

Following a previously reported procedure, ester **S3** was synthesized as a light yellow oil, (1.2 g, 70%). R_f = 0.40 (95:5, hexanes/EtOAc). ^{1}H and ^{13}C NMR are consistent with literature reports.[i]

^{1}H NMR (500 MHz, $CDCl_3$) δ 7.35 (d, *J* = 16.0 Hz, 1H), 5.86 (d, *J* = 16.0 Hz, 1H), 5.36 – 5.32 (m, 2H), 4.21 (q, *J* = 7.2 Hz, 2H), 1.88 (dd, *J* = 1.4, 0.8 Hz, 3H), 1.30 (t, *J* = 7.1 Hz, 3H) ppm.

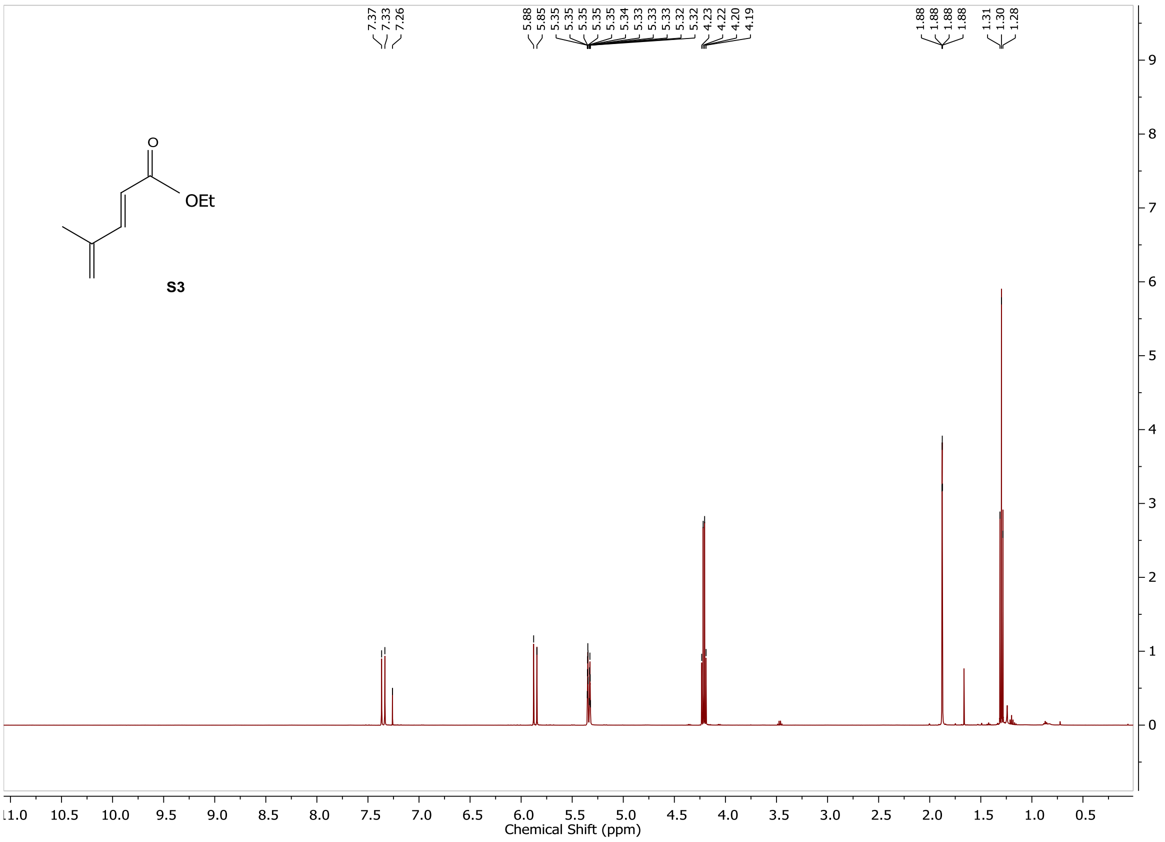

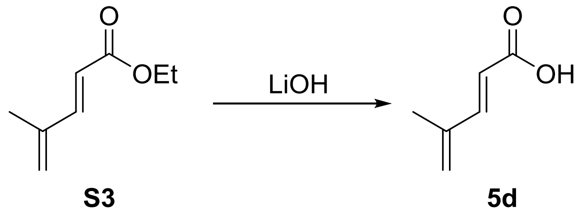

(*E*)-4-Methylpenta-2,4-dienoic acid (5d)

Following a previously reported procedure, acid **5d** was synthesized as an off-white solid, (71 mg, 90%), R_f = 0.55 (50:50, hexanes/EtOAc, large streak). ^{1}H and ^{13}C NMR are consistent with literature reports.[i]

^{1}H NMR (500 MHz, $CDCl_3$) δ 7.45 (d, *J* = 15.7 Hz, 1H), 5.88 (d, *J* = 15.7 Hz, 1H), 5.41 (s, 1H), 5.40 (s, 1H), 1.91 (d, *J* = 1.0 Hz, 3H) ppm.

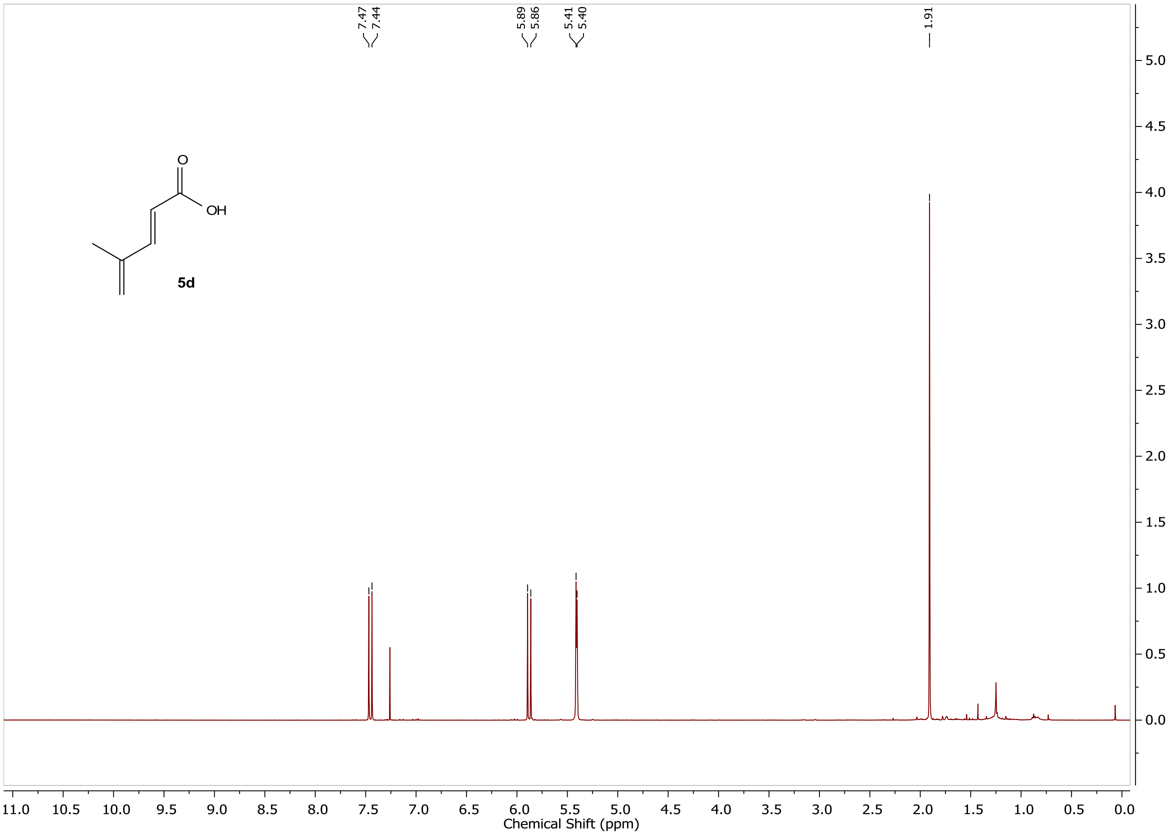

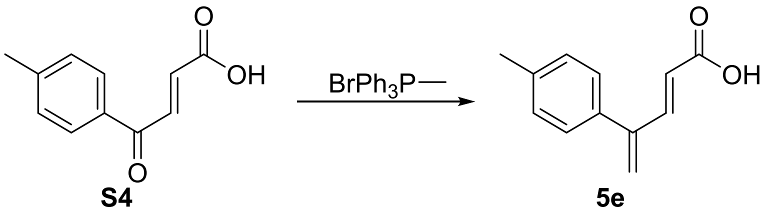

(*E*)-4-(*p*-Tolyl)penta-2,4-dienoic acid (5e)

To a solution of *trans*-3-(4-methylbenzoyl)acrylic acid (**S4**, 1.0 g, 5.2 mmol) and methyltriphenylphosphonium bromide (2.8 g, 7.9 mmol) at −78 °C in THF (70 mL) was added dropwise a solution of potassium *tert*-butoxide (1.1 g, 10 mmol) in THF (12 mL). The reaction mixture was allowed to warm gradually to room temperature for 2.5 hours and turned from yellow to dark orange. The mixture was quenched with water at 0 °C, acidified with 10% aqueous HCl to a pH ≈ 3 and extracted 3× with EtOAc. The combined organic layers were dried using Na_2SO_4 and concentrated. Purification via silica gel chromatography (95:5, hexanes/EtOAc) yielded acid **5e** (299 mg, 31%) as a yellow oil. R_f = 0.79 (95:5, hexanes/EtOAc). This compound was found to be unstable at room temperature for several hours and it was taken directly to the next reaction. Due to the instability, HRMS data was not obtained and the NMR spectra contained moderate solvent impurities. These impurities were taken into account when determining the yield of **5e**.

^{1}H NMR (400 MHz, $CDCl_3$) δ 7.62 (d, *J* = 15.9 Hz, 1H), 7.18 (s, 4H), 5.86 (d, *J* = 15.8 Hz, 1H), 5.64 (s, 1H), 5.54 (s, 1H), 2.35 (s, 3H) ppm.

^{13}C NMR (100 MHz, $CDCl_3$) δ 172.4, 148.3, 146.2, 138.0, 135.4, 129.2 (2C), 128.2 (2C), 124.9, 120.7, 21.3 ppm.

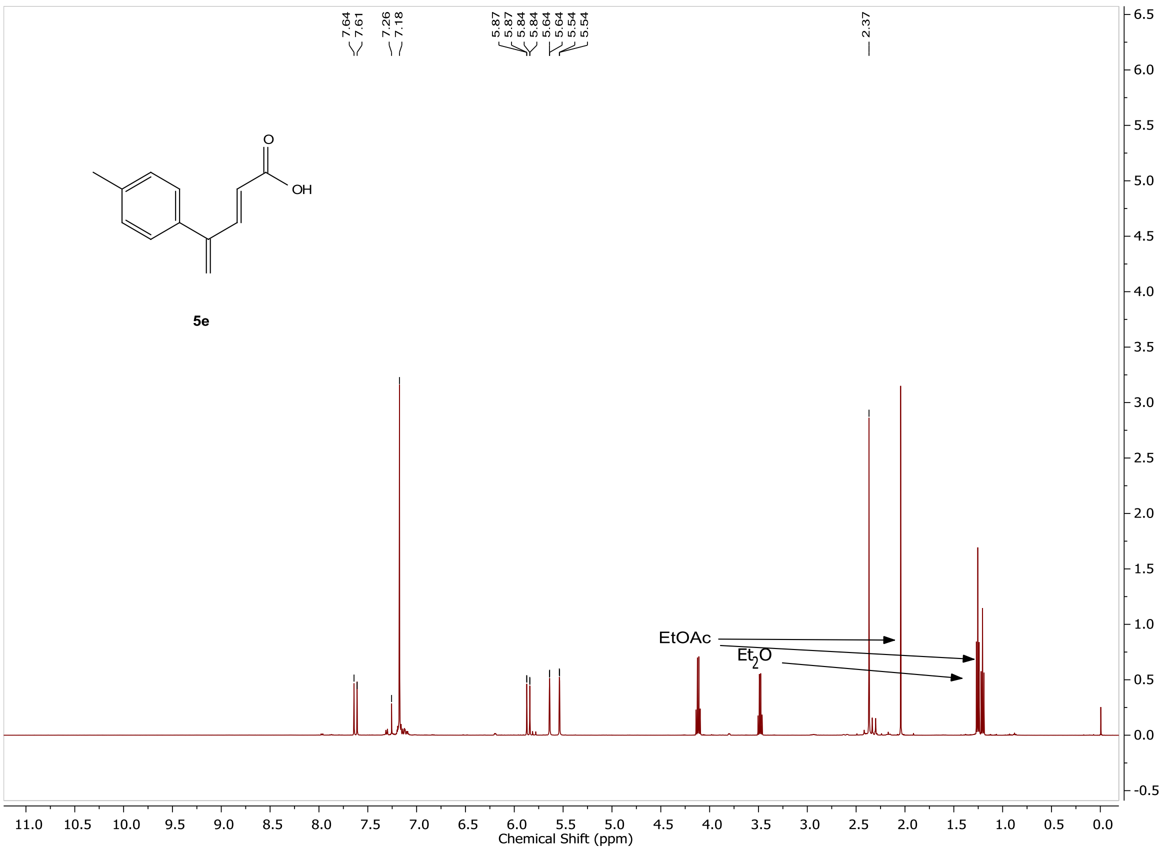

7.64
7.61
7.26
7.18
5.87
5.87
5.84
5.84
5.64
5.64
5.54
5.54
2.37
OH
O
5e
EtOAc
Et_2O
Chemical Shift (ppm)

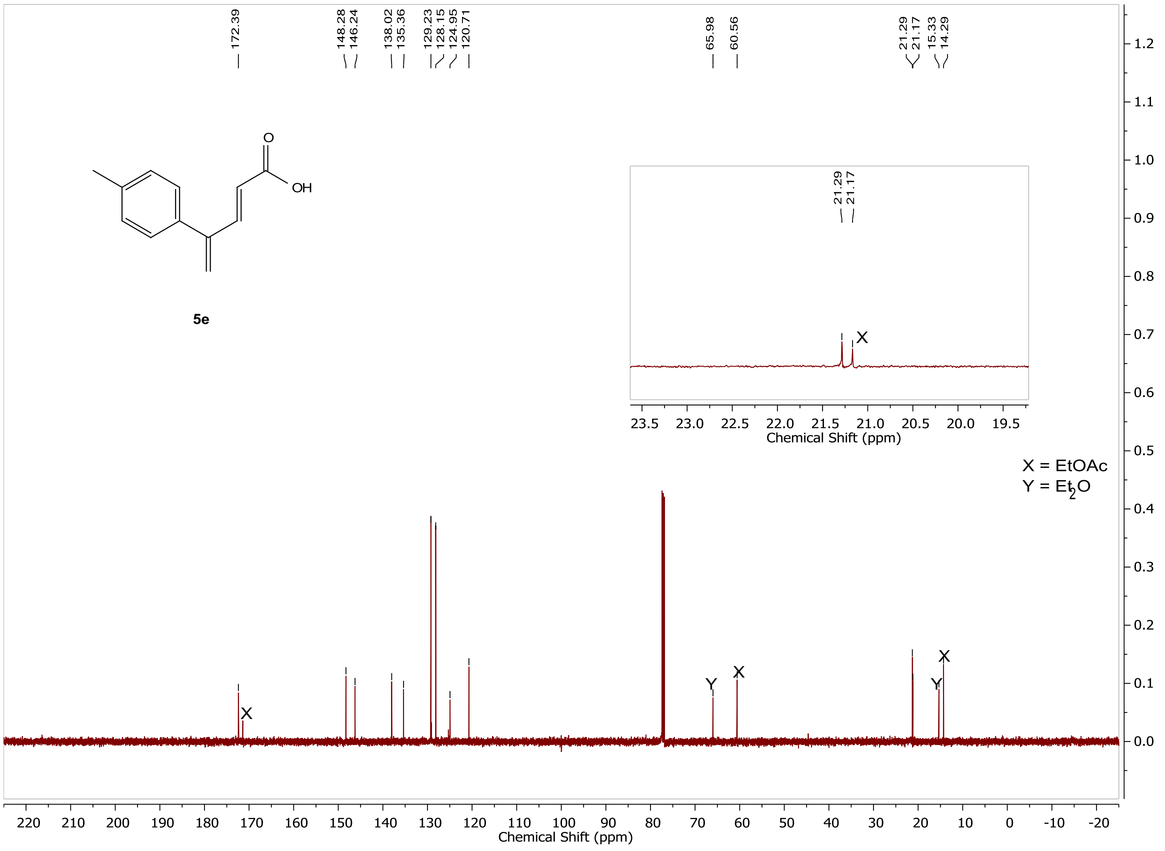

172.39
148.28
146.24
138.02
135.36
129.23
128.15
124.95
120.71
65.98
60.56
21.29
21.17
15.33
14.29
OH
O
5e
X = EtOAc
Y = Et_2O
Chemical Shift (ppm)

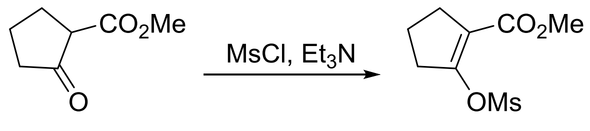

Methyl 2-((methylsulfonyl)oxy)cyclopent-1-enecarboxylate (S6)

Following a previously reported procedure, mesylate **S6** was synthesized as a brown oil (434 mg, 40% yield). R_f = 0.29 (70:30, hexanes/EtOAc). ^{1}H and ^{13}C NMR are consistent with literature reports.[ii]

^{1}H NMR (500 MHz, $CDCl_3$) δ 3.74 (s, 3H), 3.25 (s, 3H), 2.80 (tt, *J* = 8.0, 2.7 Hz, 1H), 2.64 (tt, *J* = 7.7, 2.6 Hz, 2H) 1.97 (quint, *J* = 7.8 Hz, 2H) ppm.

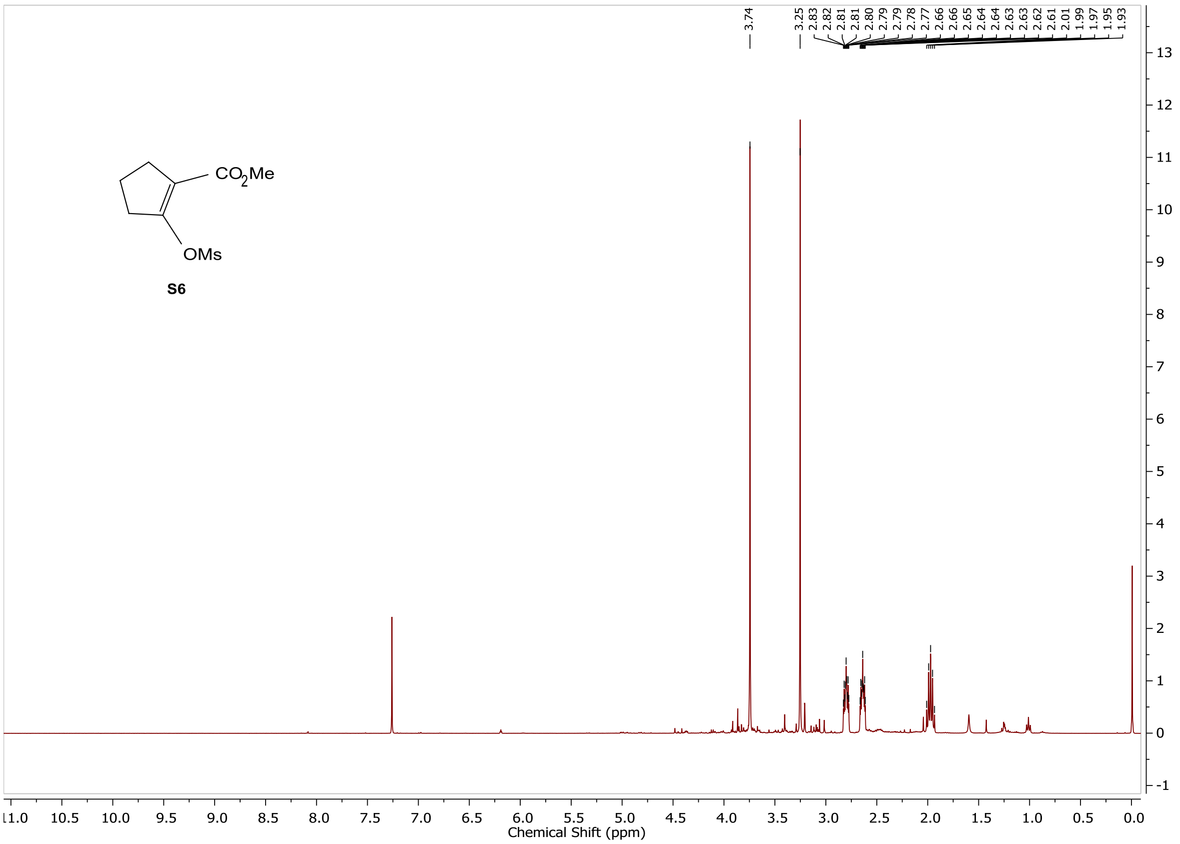

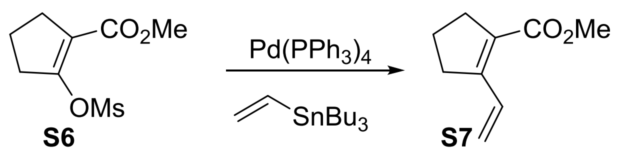

Methyl 2-vinylcyclopent-1-ene-1-carboxylate (S7)

To a solution of LiBr (356 mg, 4.1 mmol) and $Pd(PPh_3)_4$ (316 mg, 0.27 mmol) in THF (17 mL) was added a solution of methyl 2-((methylsulfonyl)oxy)cyclopent-1-ene-1-carboxylate (**S6**, 600 mg, 2.7 mmol) and vinyltributylstannane (1.6 mL, 5.9 mmol) in THF (10 mL) under an atmosphere of N_2. The solution was heated in a microwave reactor at 90 °C for 24 h, cooled to room temperature, diluted with CH_2Cl_2 and washed with water. The aqueous layer was back extracted with CH_2Cl_2, and the combined organic layers were washed with 10% NH_4OH solution, water and saturated aqueous NaCl, dried over Na_2SO_4 and concentrated. Purification via silica gel chromatography (97:2, hexanes/diethyl ether) yielded ester **S7** (250 mg, 60%) as a colorless oil. R_f = 0.52 (90:10, hexanes/EtOAc). ^{1}H and ^{13}C NMR are consistent with literature reports.[iii]

^{1}H NMR (400 MHz, $CDCl_3$) δ 7.51 (dd, *J* = 17.6, 10.8 Hz, 1H), 5.44 – 5.38 (m, 2H), 3.74 (s, 3H), 2.72 – 2.65 (m, 4H), 1.86 (quint, *J* = 7.6 Hz, 2H) ppm.

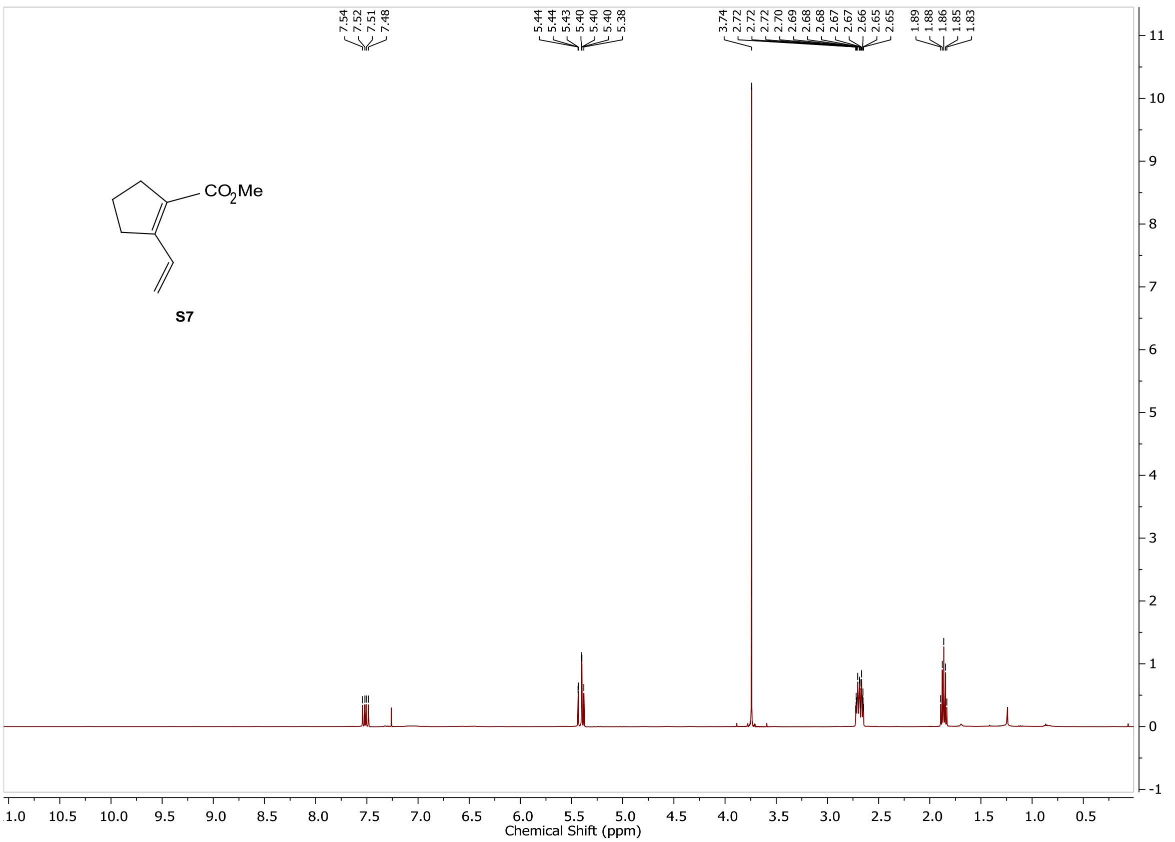

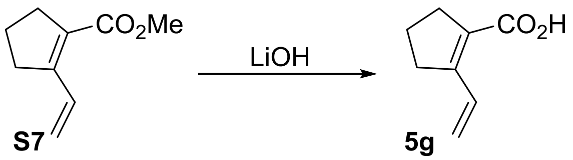

2-Ethenyl-1-cyclopentenecarboxylic acid (5g)

Following a previously reported procedure, acid **5g** was synthesized as an off-white solid (38 mg, 83% yield). R_f = 0.90 (89:10:1, hexanes/EtOAc/AcOH). ^{1}H and ^{13}C NMR are consistent with literature reports.[iv]

^{1}H NMR (400 MHz, $CDCl_3$) δ 12.24 (bs, 1H), 7.56 (dd, *J* = 17.6, 10.8 Hz, 1H), 5.47 (dt, J_d = 17.6 Hz, J_t = 0.6 Hz, 1H), 5.44 (d, *J* = 10.8 Hz, 1H), dtd (J_d = 9.8, 1.6, J_t = 7.8, 4H), 1.89 (quint, *J* = 7.7 Hz, 2H) ppm.

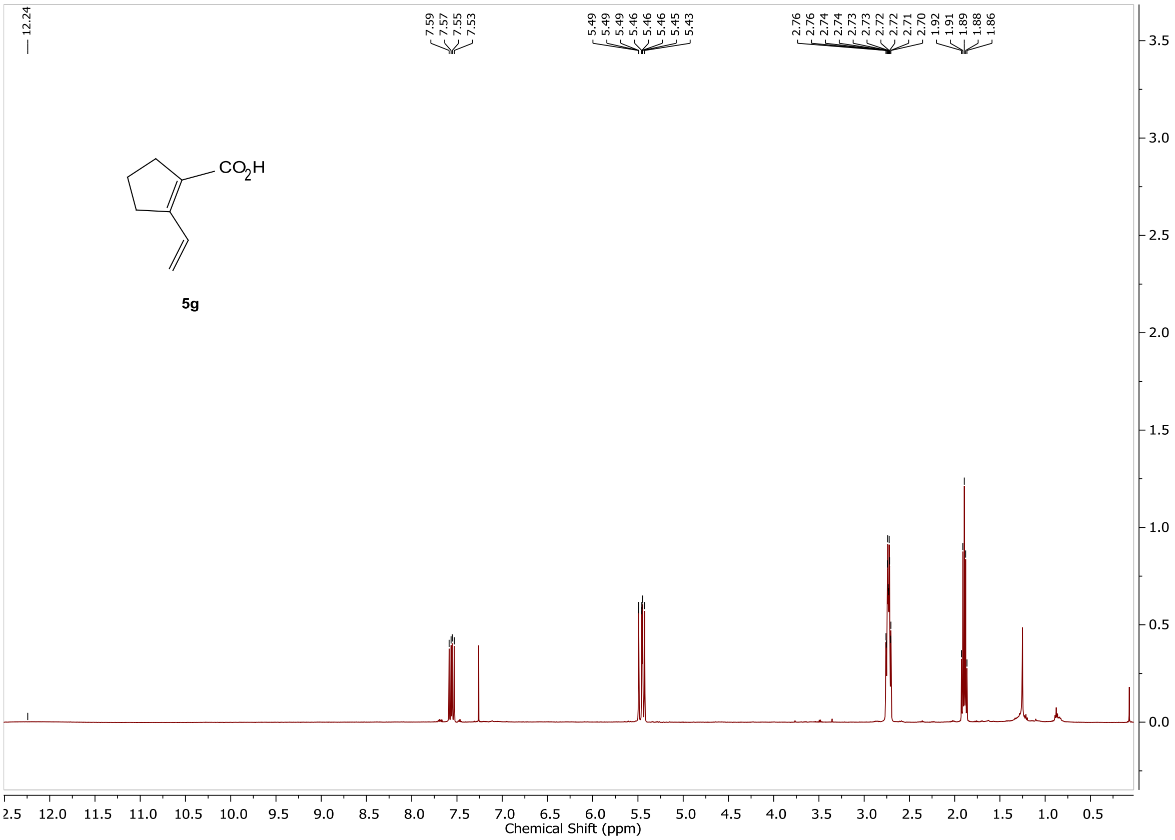

Experimental procedures and characterization of pentadienyl groups

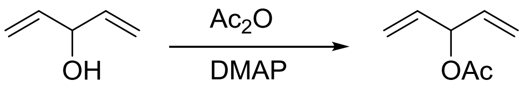

6e **6a**

Penta-1,4-dien-3-yl acetate (6a)

Following a previously reported procedure, acetate **6a** was synthesized as a colorless liquid, (1.6 g, 64%). R_f = 0.44 (80:20, hexanes/EtOAc). ^{1}H and ^{13}C NMR are consistent with literature reports.[v]

^{1}H NMR (400 MHz, $CDCl_3$) δ 5.83 (ddd, *J* = 17.0, 10.5, 6.0 Hz, 2H), 5.69 (tt, *J* = 6.0, 1.2 Hz, 1H), 5.29 (dt, J_d = 17.1 Hz, J_t = 1.1 Hz, 2H), 5.22 (dt, J_d = 10.5 Hz, J_t = 1.2 Hz, 2H), 2.09 (s, 3H) ppm.

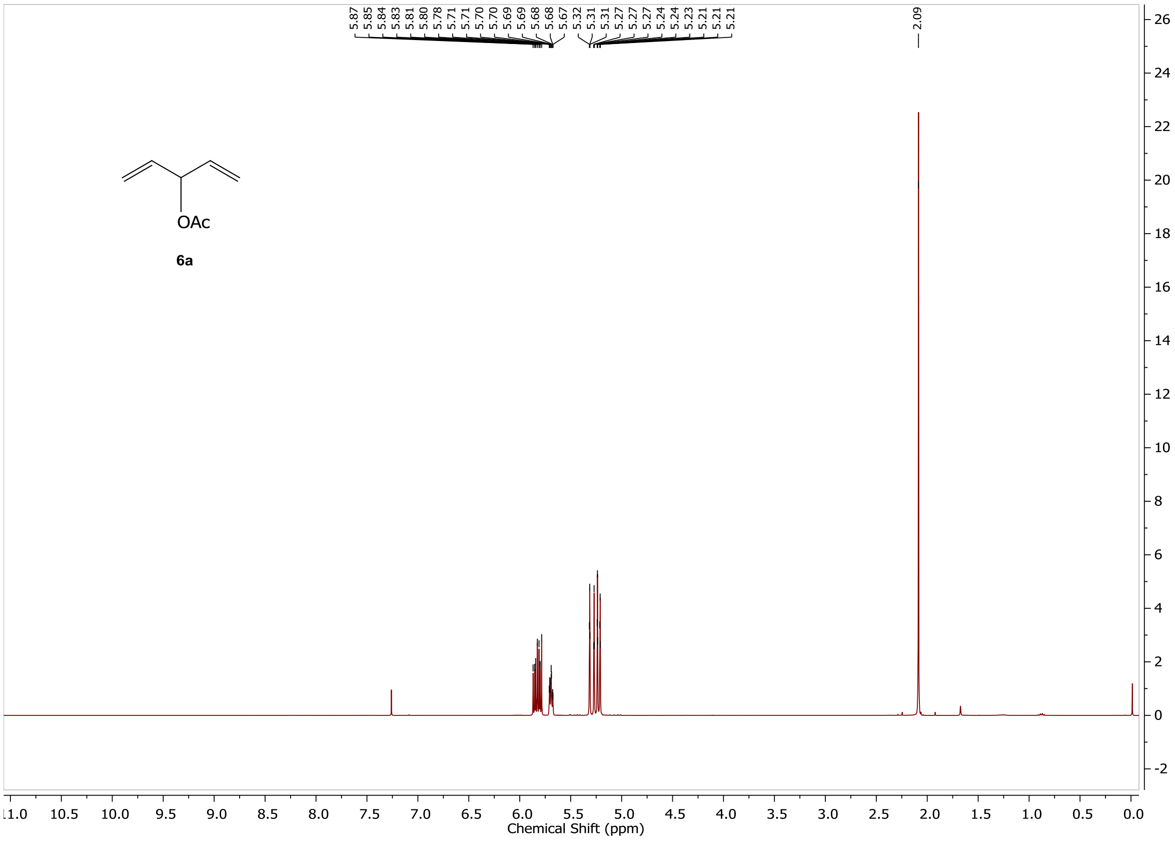

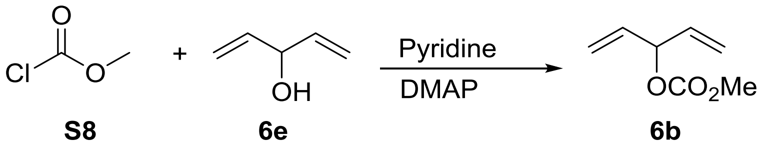

Methyl penta-1,4-dien-3-yl carbonate (6b)

Following a previously reported procedure, carbonate **6b** was synthesized as a yellow liquid, (202 mg, 36%). R_f = 0.30 (80:20, hexanes/EtOAc). ^{1}H and ^{13}C NMR are consistent with literature reports.[vi]

^{1}H NMR (500 MHz, $CDCl_3$) δ 5.90 – 5.82 (m, 2H), 5.52 (tt, J = 6.3, 1.2 Hz, 1H), 5.35 (dt, J_d = 17.2, J_t = 1.2 Hz, 2H), 5.27 (dt, J_d = 10.5, J_t = 1.2 Hz, 2H), 3.79 (s, 3H) ppm.

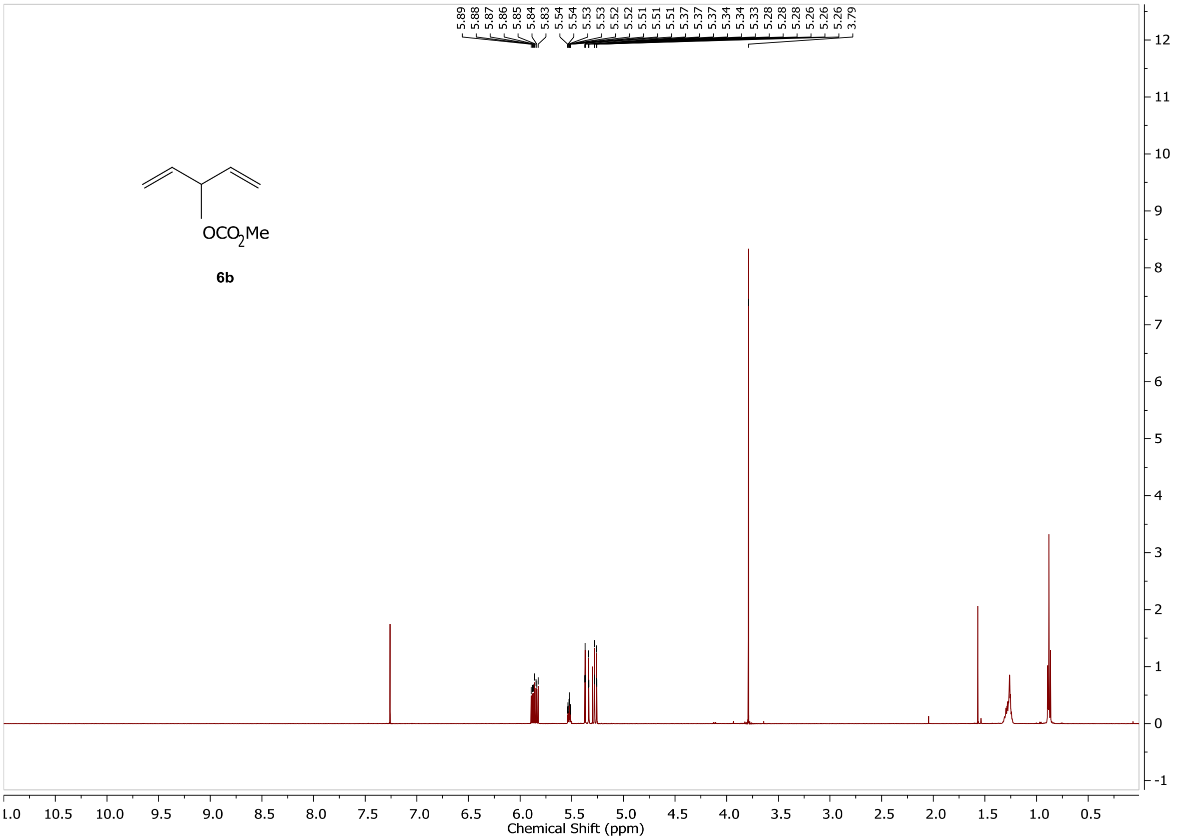

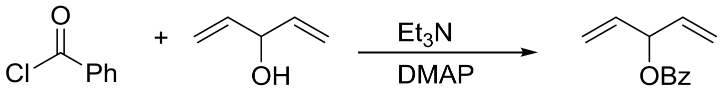

S9 **6e** **6c**

Penta-1,4-dien-3-yl benzoate (6c)

Benzoyl chloride **S9** (5.2 mL, 45 mmol), Et_3N (6.3 mL, 45 mmol) and DMAP (183 mg, 1.5 mmol) were sequentially added slowly to a precooled solution of 1,4-pentadien-3-ol (**6e**, 1.5 mL, 15 mmol) in CH_2Cl_2 (33 mL) at 0 °C. After stirring for 18 hours at room temperature, the reaction was quenched with saturated NH_4Cl solution, washed with hexane and extracted with CH_2Cl_2. The organic layers were combined, dried using Na_2SO_4 and concentrated. Purification via silica gel chromatography (90:10, hexanes/EtOAc) yielded benzoate **6c** (1.4 g, 48%) as a colorless liquid. R_f = 0.18 (90:10, hexanes/EtOAc). ^{1}H and ^{13}C NMR are consistent with literature reports.[xii]

^{1}H NMR (400 MHz, $CDCl_3$) δ 8.10 – 8.08 (m, 2H), 7.57 (t, *J* = 7.4 Hz, 1H), 7.45 (t, *J* = 7.7 Hz, 2H), 6.01 – 5.92 (m, 3H), 5.43 – 5.38 (m, 2H), 5.29 (d, *J* = 9.6 Hz, 2H) ppm.

^{13}C NMR (100 MHz, $CDCl_3$) δ 165.6, 135.1 (2C), 133.1, 130.4, 129.8 (2C), 128.5 (2C), 117.7 (2C), 75.6 ppm.

HRMS (APPI) calcd. for $[C_{12}H_{12}O_2+H]^+$: 189.0913, found: 189.0909.

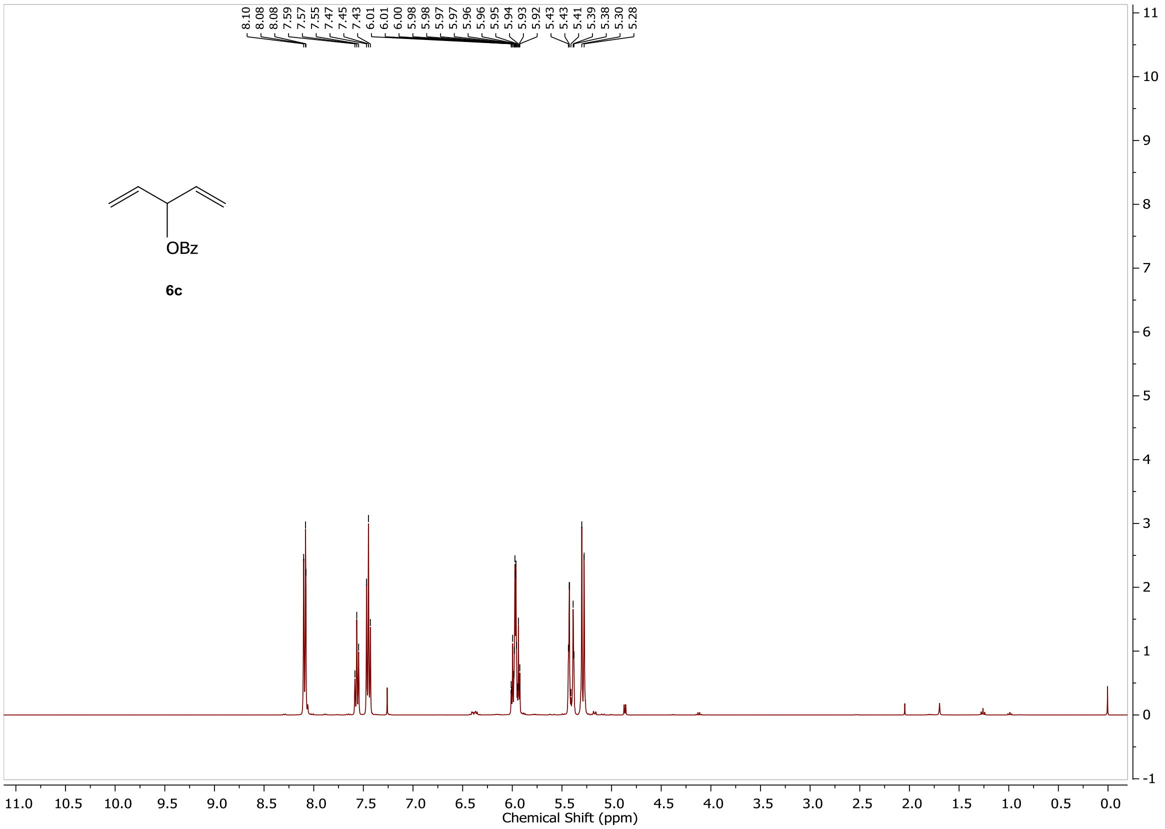

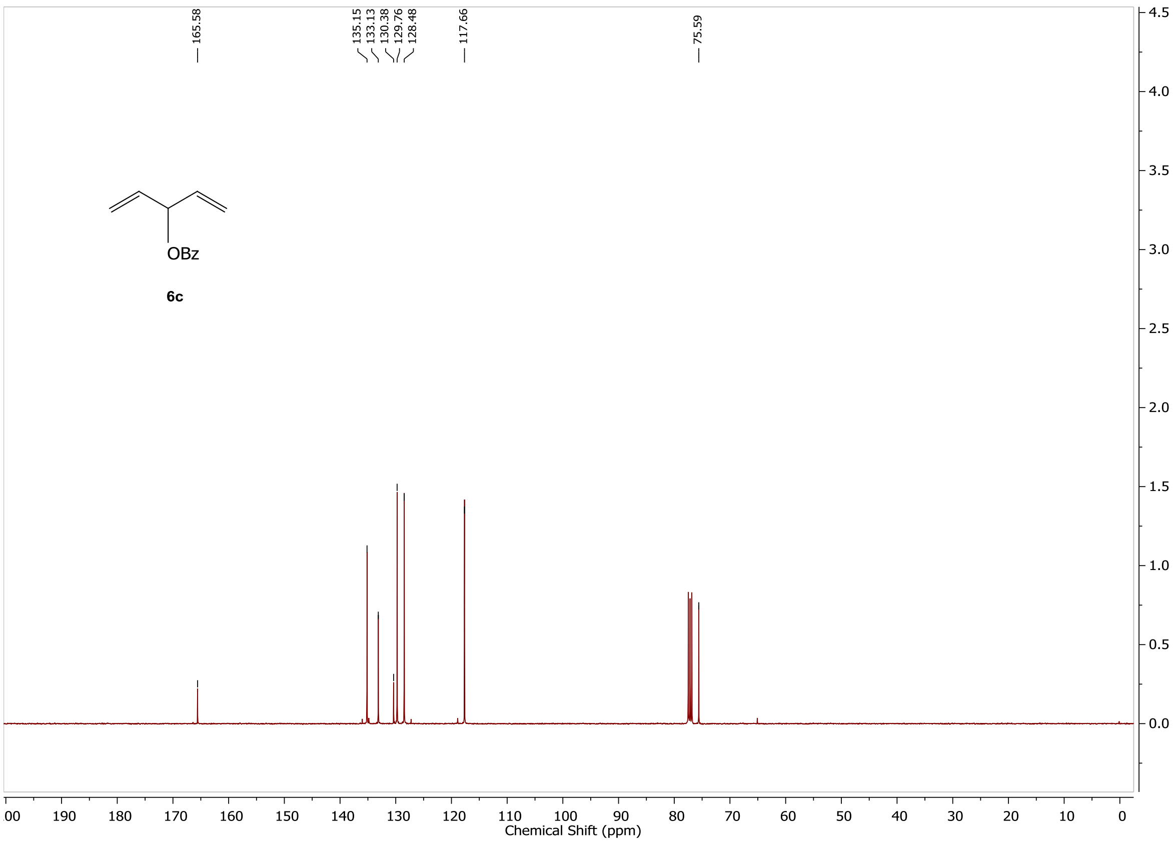

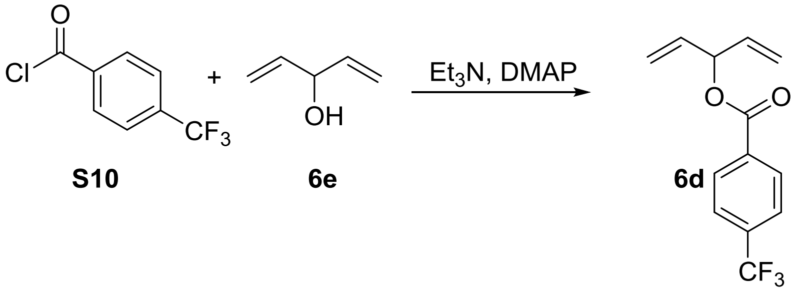

Penta-1,4-dien-3-yl 4-trifluoromethylbenzoate (6d)

To a solution of 1,4-pentadien-3-ol **6e** (0.25 mL, 2.6 mmol) in CH_2Cl_2 (26 mL) precooled to 0 °C, were sequentially added 4-(trifluoromethyl)benzoyl chloride (**S10**, 0.4 mL, 2.6 mmol), Et_3N (1.1 mL, 7.7 mmol) and DMAP (31 mg, 0.25 mmol). After stirring for 18 hours at room temperature, the reaction was quenched with saturated NH_4Cl solution, washed with hexane and extracted with CH_2Cl_2. The organic layers were combined and dried using Na_2SO_4 and concentrated. Purification via silica gel chromatography (90:10, hexanes/EtOAc) yielded benzoate **6d** (529 mg, 83%) as a faint yellow liquid. R_f = 0.82 (80:20, hexanes/EtOAc).

^{1}H NMR (400 MHz, $CDCl_3$) δ 8.19 (d, J = 8.2 Hz, 2H), 7.71 (d, J = 8.2 Hz, 2H), 6.01 – 5.92 (m, 3H), 5.44 – 5.38 (m, 2H), 5.31 (d, J = 9.6 Hz, 2H) ppm.

^{13}C NMR (100 MHz, $CDCl_3$) δ 164.4, 134.8 (2C), 134.5 (q, $J_{C\text{-}F}$ = 45 Hz, 1C), 133.7, 130.2 (2C), 125.5 (q, $J_{C\text{-}F}$ = 4.0 Hz, 2C), 123.7 (q, $J_{C\text{-}F}$ = 272 Hz, 1C), 118.1 (2C), 76.3 ppm.

^{19}**F NMR** (376.5 MHz, $CDCl_3$) δ -63.00 (s, 3F) ppm.

HRMS (APPI) calcd. for [$C_{13}H_{11}O_2F_3$]$^+$: 256.0711, found: 256.0706.

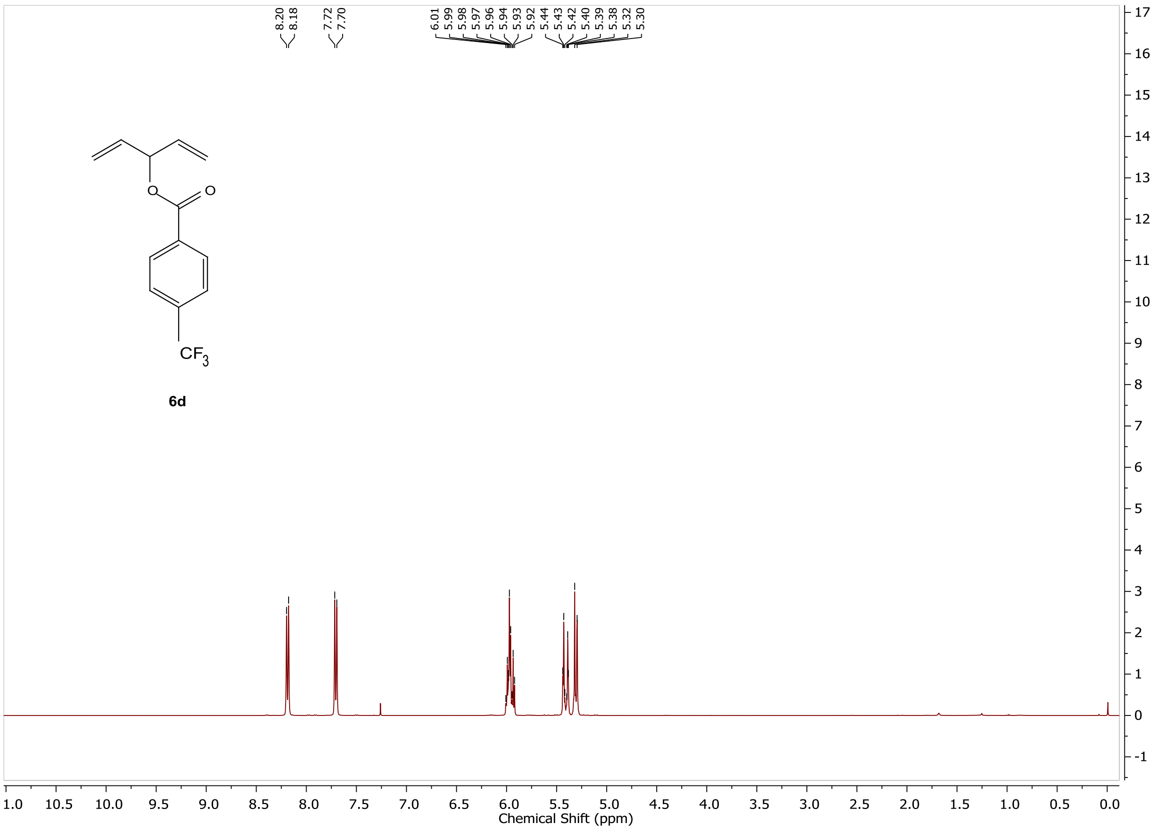

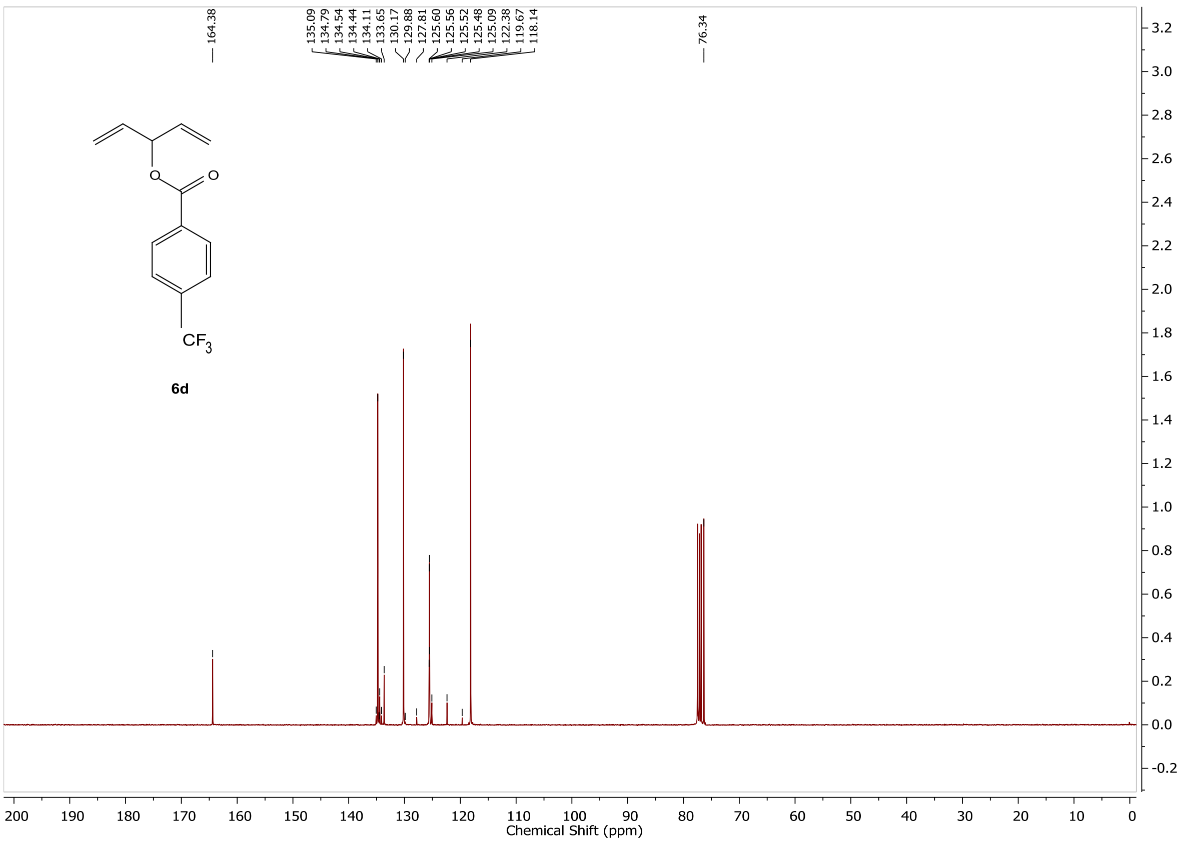
164.38
135.09
134.79
134.54
134.44
134.11
133.65
130.17
129.88
127.81
125.60
125.56
125.52
125.48
125.09
122.38
119.67
118.14
76.34
O
O
CF3
Chemical Shift (ppm)

6d

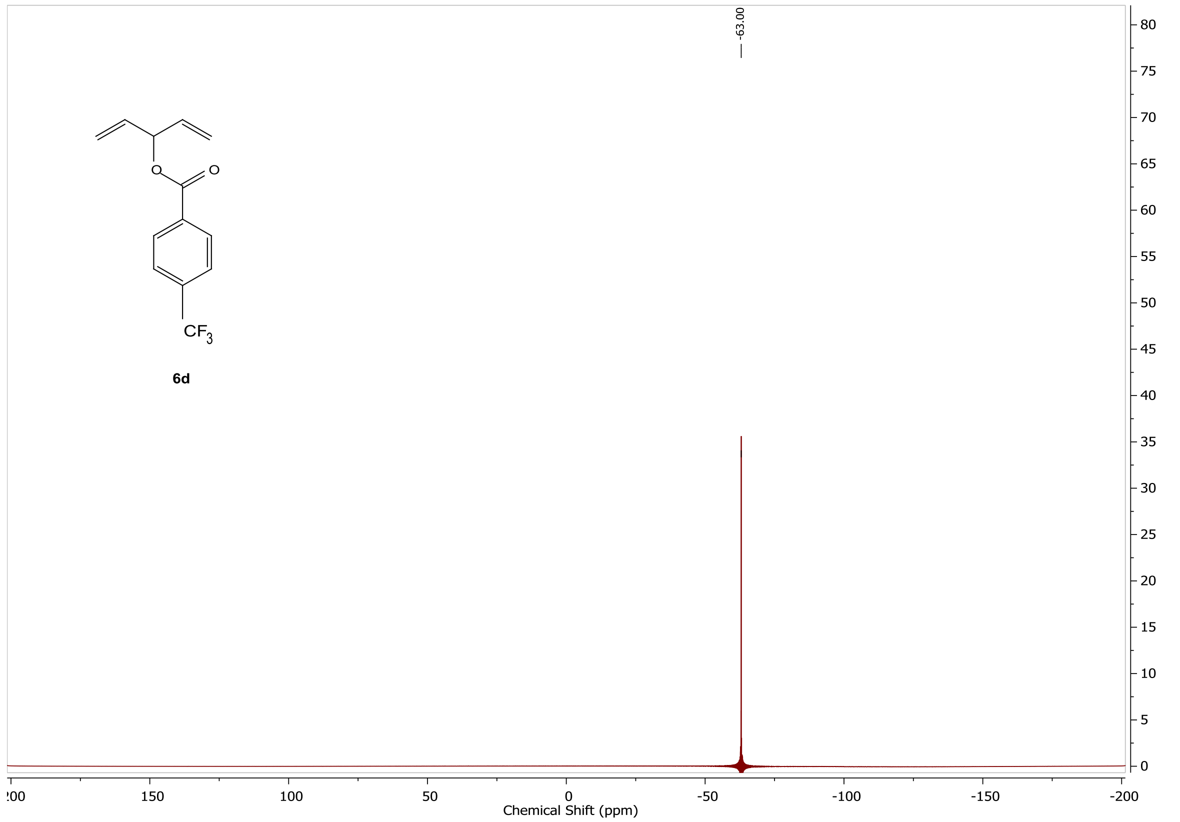
-63.00
O
O
CF3
Chemical Shift (ppm)

6d

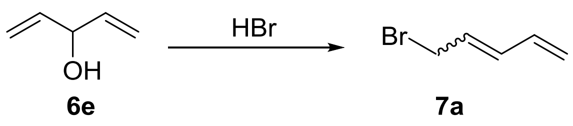

(*E*)-5-Bromo-1,3-diene (7a)

To a stirred solution of 1,4-pentadien-3-ol (**6e**, 0.50 mL, 5.1 mmol) in pentane (0.4 mL) at 0 °C, was added HBr (0.5 mL, 9.2 mmol, 48%) dropwise. After stirring for 4 hours at room temperature, the reaction mixture was diluted with diethyl ether, quenched with a saturated, aqueous sodium bicarbonate solution, extracted with diethyl ether and dried over Na_2SO_4. The solution was concentrated and purified via silica gel chromatography (pentane) to give **7a** (183 mg, 24%) as a colorless liquid. The impurities were taken into account when determining the yield of **7a**. R_f = 0.90 (80:20, hexanes/EtOAc). The product is a mixture of two diastereomers in a 10:1 ratio. Only the major, *E* diastereomer, was fully characterized. ^{1}H and ^{13}C NMR are consistent with literature reports.[vii]

^{1}H NMR (400 MHz, $CDCl_3$) δ 6.38 – 6.26 (m, 2H), 5.89 (dt, J_d = 13.6, J_t = 7.8 Hz, 1H), 5.30 – 5.26 (m, 1H), 5.17 (dd, J = 9.3, 1.7 Hz, 1H), 4.03 (d, J = 8.0 Hz, 2H) ppm.

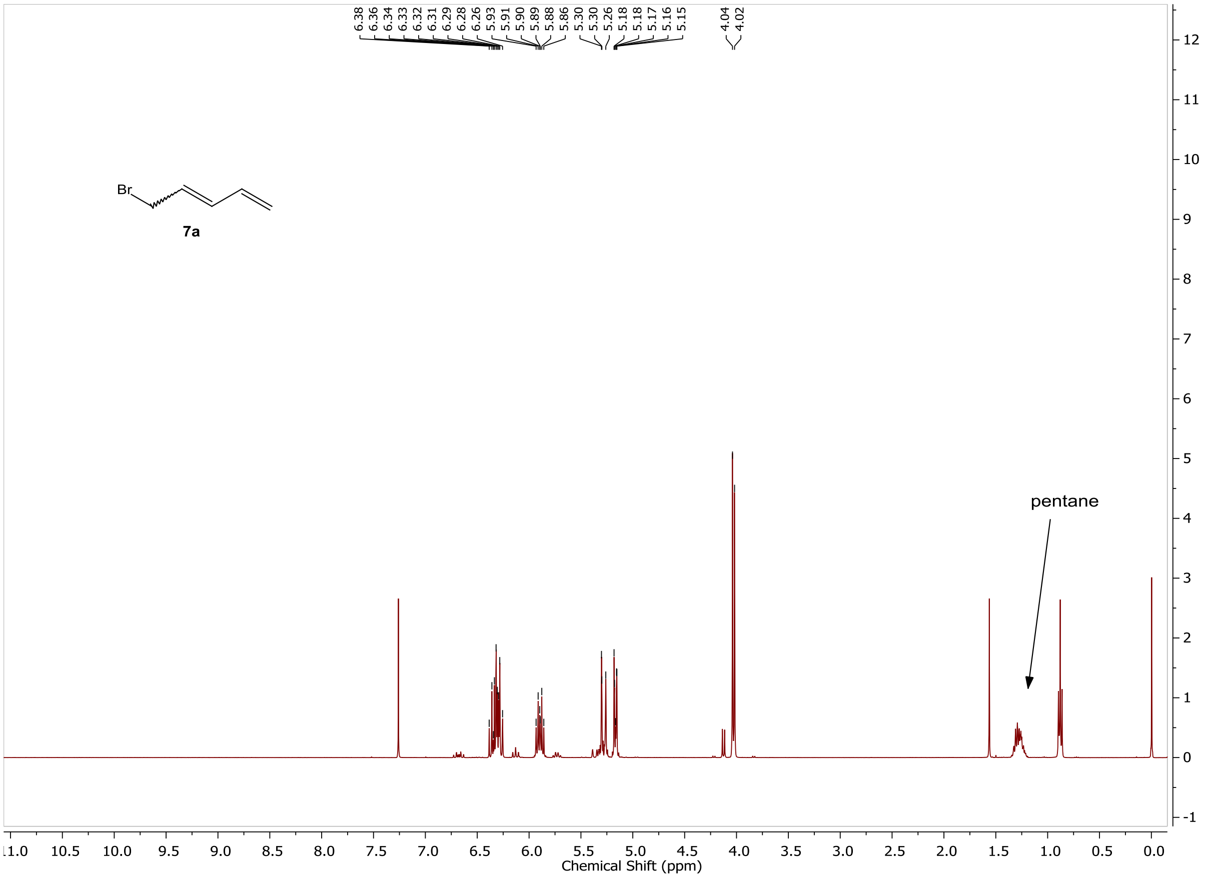

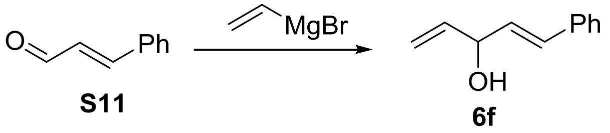

(1*E*)-1-Phenylpenta-1,4-dien-3-ol (6f)

Vinyl magnesium bromide (0.7 M in THF, 13 mL, 9.1 mmol) was added dropwise to a precooled solution of *trans*-cinnamaldehyde **S11** (0.95 ml, 7.6 mmol) in THF (10 mL) at 0 °C. The reaction mixture was allowed to warm to room temperature and stirred for 4 hours before it was quenched with saturated NH_4Cl solution and extracted with ethyl acetate. The organic layers were combined, dried using $MgSO_4$, and filtered. The solution was concentrated and purified via silica gel chromatography (80:20, hexanes/EtOAc) which yielded alcohol **6f** (938 mg, 86%) as a yellow oil. R_f = 0.26 (80:20, hexanes/EtOAc). ^{1}H and ^{13}C NMR are consistent with literature reports.[viii]

^{1}H NMR (400 MHz, $CDCl_3$) δ 7.41 – 7.39 (m, 2H), 7.34 – 7.30 (m, 2H), 7.25 (t, J = 7.0 Hz, 1H), 6.62 (d, J = 16.0 Hz, 1H), 6.24 (dd, J = 16.0, 6.4 Hz, 1H), 5.98 (ddd, J = 16.8, 10.5, 5.9 Hz, 1H), 5.35 (dt, J_d = 17.5, J_t = 1.4 Hz, 1H), 5.21 (dt, J_d = 10.3, J_t = 1.1 Hz, 1H), 4.82 (t, J = 6.4 Hz, 1H), 1.75 (bs, 1H) ppm.

^{13}C NMR (100 MHz, $CDCl_3$) δ 139.3, 136.7, 130.9, 130.4, 128.7 (2C), 127.9, 126.7 (2C), 115.6, 73.9 ppm.

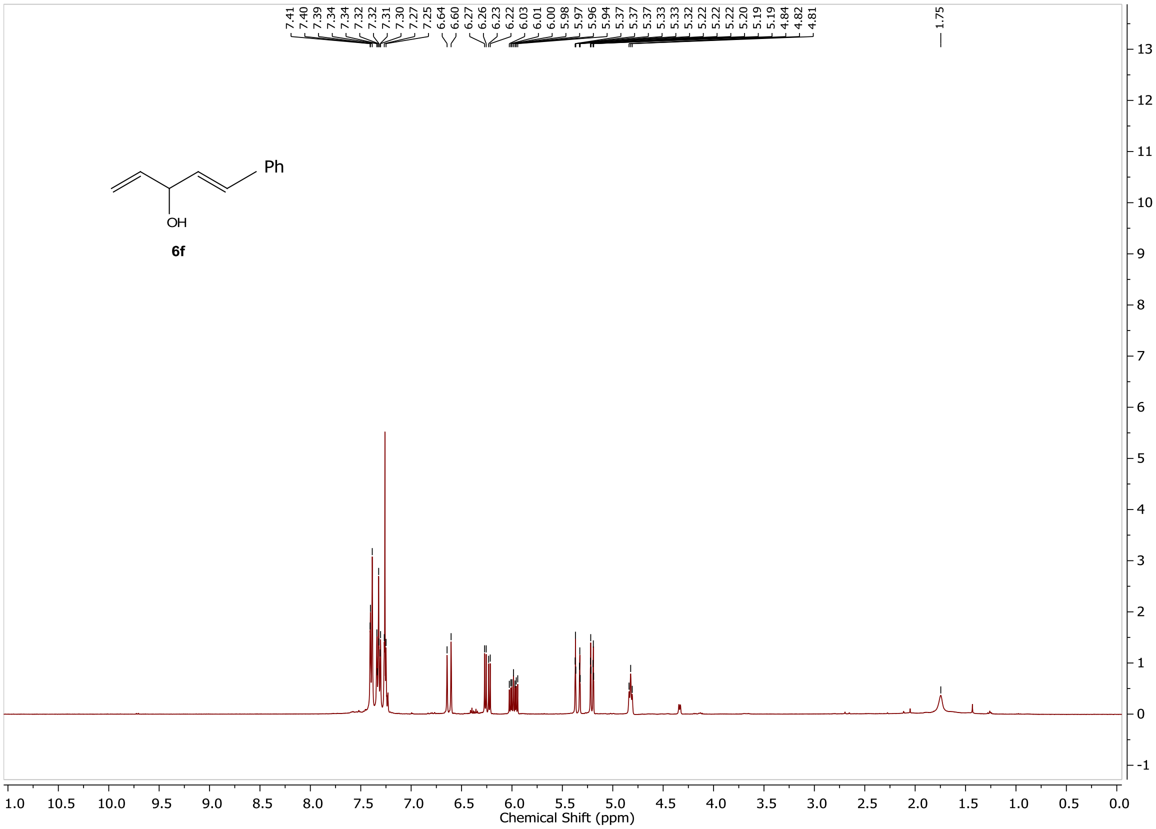

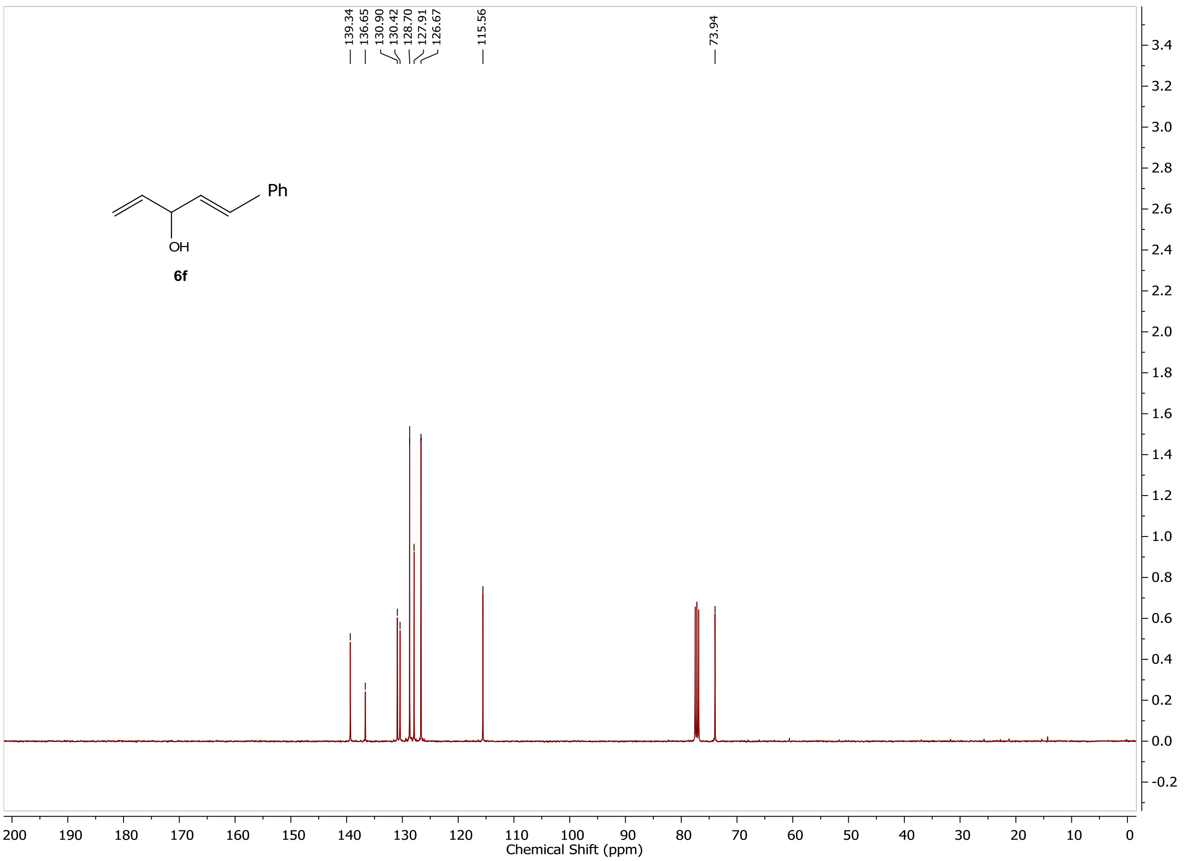

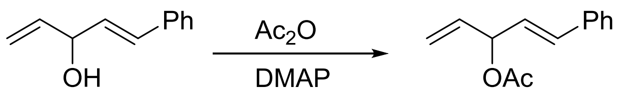

(*E*)-1-Phenylpenta-1,4-dien-3-yl acetate (6g)

Acetic anhydride (0.45 mL, 4.3 mmol) was added dropwise to a solution of DMAP (0.95 g, 0.78 mmol) and (*E*)-1-phenylpenta-1,4-dien-3-ol (**6f**, 0.55 g, 3.5 mmol) in CH_2Cl_2 (7.1 mL) at 0 °C. The reaction mixture was allowed to warm to room temperature and stirred for 24 hours before it was concentrated under reduced pressure and purified via silica gel chromatography (97:3, hexanes/EtOAc) to yield acetate **6g** (168 mg, 53%) as a yellow oil. R_f = 0.56 (97:3, hexanes/EtOAc). ^{1}H and ^{13}C NMR are consistent with literature reports.[ix]

^{1}H NMR (400 MHz, $CDCl_3$) δ 7.32 – 7.21 (m, 3H), 7.19 – 7.15 (m, 2H), 6.56 (d, *J* = 16.0 Hz, 1H), 6.25 – 6.12 (m, 1H), 6.10 (dd, *J* = 16.0, 6.8 Hz, 1H), 5.89 – 5.74 (m, 1H), 5.30 – 5.26 (m, 1H), 5.20 – 5.17 (m, 1H), 2.03 (s, 3H) ppm.

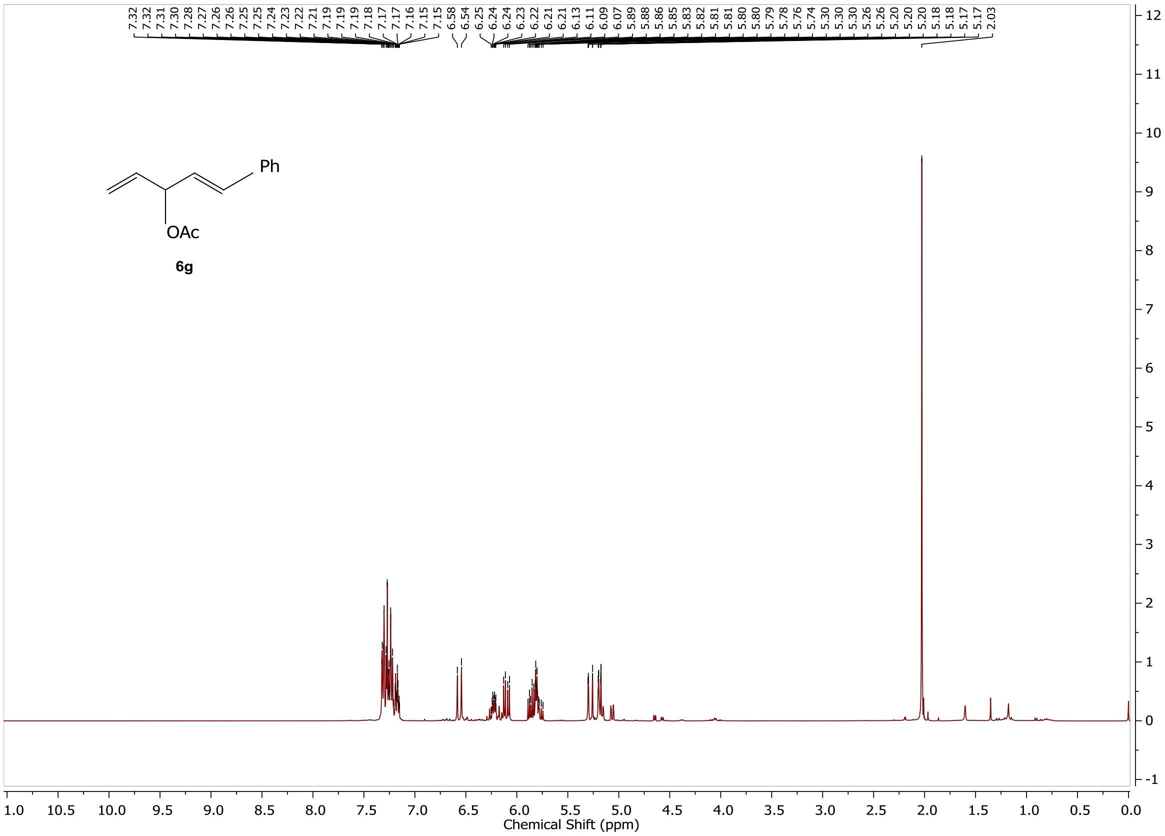

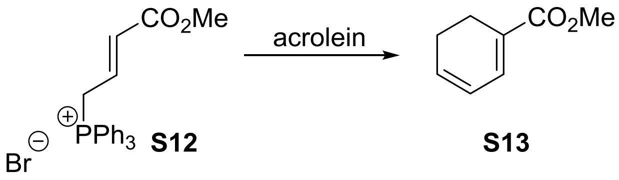

Methyl cyclohexa-1,3-diene-1-carboxylate (S13)

Following a previously reported procedure, ester **S13**[x] was synthesized as a colorless oil, (350 mg, 30%). R_f = 0.38 (75:25, hexanes/EtOAc). ^{1}H and ^{13}C NMR are consistent with literature reports.[x] This compound was prone to air oxidation to methyl benzoate so it was moved forward synthetically prior to complete removal of the solvent.

^{1}H NMR (400 MHz, $CDCl_3$) δ 7.00 – 6.96 (m, 1H), 6.10 (dt, J_d = 8.6, J_t = 4.3 Hz, 1H), 6.01 (ddt, J_d = 9.5, 5.5, J_t = 1.8 Hz, 1H), 3.71 (s, 3H), 2.48 – 2.37 (m, 2H), 2.29 – 2.21 (m, 2H) ppm.

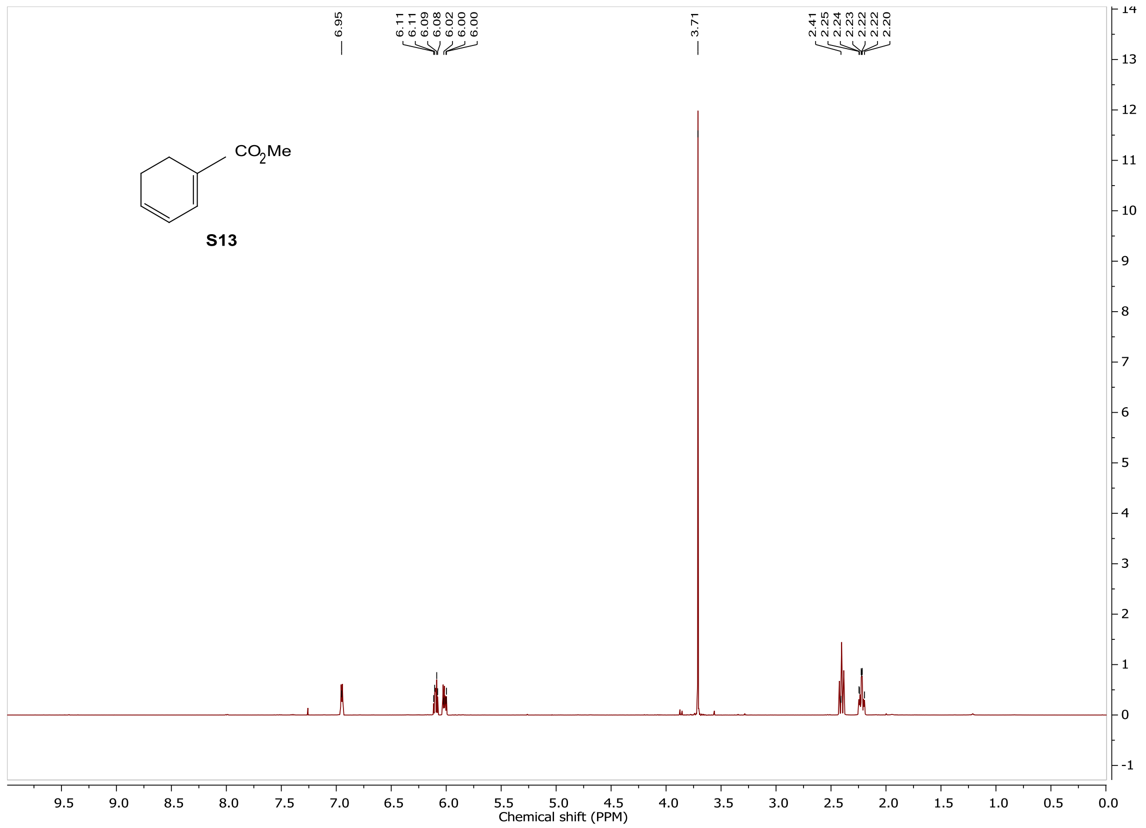

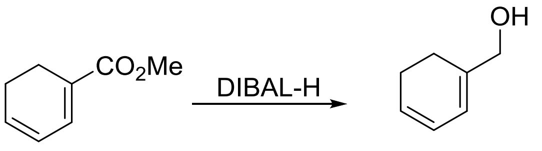

1,3-Cyclohexadiene-1-methanol (S14)

DIBAL-H in toluene (1.2 M, 4.1 mL, 5.0 mmol) was added to ester **S13** (350 mg, 2.5 mmol) in THF (10 mL) at −78 °C. The reaction was allowed to warm to room temperature and stirred overnight. The reaction mixture was filtered through Celite®, acidified with 10% aqueous HCl, extracted with diethyl ether, and dried using Na_2SO_4. The solution was concentrated and purified via silica gel chromatography (80:20, hexanes/EtOAc) to give (197 mg, 72%) of alcohol **S14**. R_f = 0.19 (80:20, hexanes/EtOAc). ^{1}H and ^{13}C NMR are consistent with literature reports.[xi] This compound was prone to air oxidation to benzyl alcohol so it was moved forward synthetically prior to complete removal of solvent. The impurities were taken into account when determining the yield of **S14**.

^{1}H NMR (400 MHz, $CDCl_3$) δ 5.94 – 5.87 (m, 2H), 5.77 (dt, J_d = 9.0, J_t = 4.0 Hz, 1H), 4.11 (s, 2H), 3.4 (s, O*H*), 2.26 – 2.13 (m, 4H) ppm.

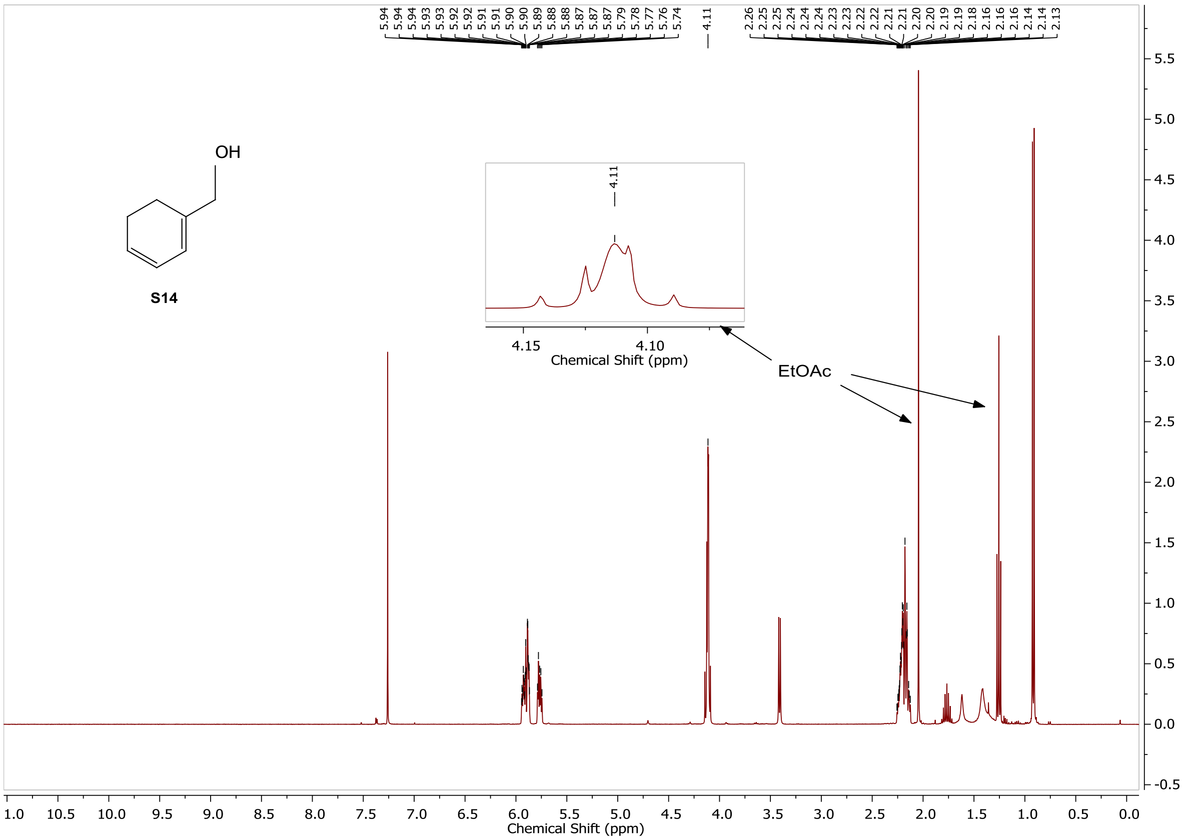

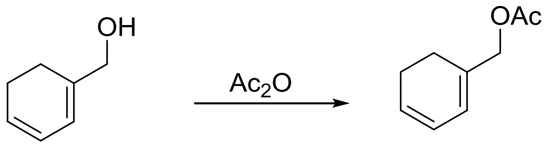

Cyclohexa-1,3-dien-1-ylmethyl acetate (7e)

To a solution of alcohol **S14** (274 mg, 2.5 mmol) in CH_2Cl_2 (25 mL) was added acetic anhydride (0.25 mL, 2.5 mmol) and DMAP (61 mg, 0.5 mmol) at 0 °C. After stirring for 18 hours at room temperature, the reaction was diluted with CH_2Cl_2 and washed with water. The aqueous layer was back extracted with CH_2Cl_2, and the combined organic layers were dried using Na_2SO_4 and concentrated to give acetate **7e** (171 mg, 45%) as a yellow liquid. R_f = 0.65 (90:10, hexanes/EtOAc). Due to the instability, HRMS data was not obtained.

^{1}H NMR (400 MHz, $CDCl_3$) δ 5.92 – 5.89 (m, 2H), 5.81 – 5.77(m, 1H), 4.55 (s, 2H), 2.25 – 2.10 (m, 4H), 2.09 (s, 3H) ppm.

^{13}C NMR (100 MHz, $CDCl_3$) δ 171.1, 132.8, 126.6, 123.9, 122.2, 67.7, 23.6, 22.6, 21.1 ppm.

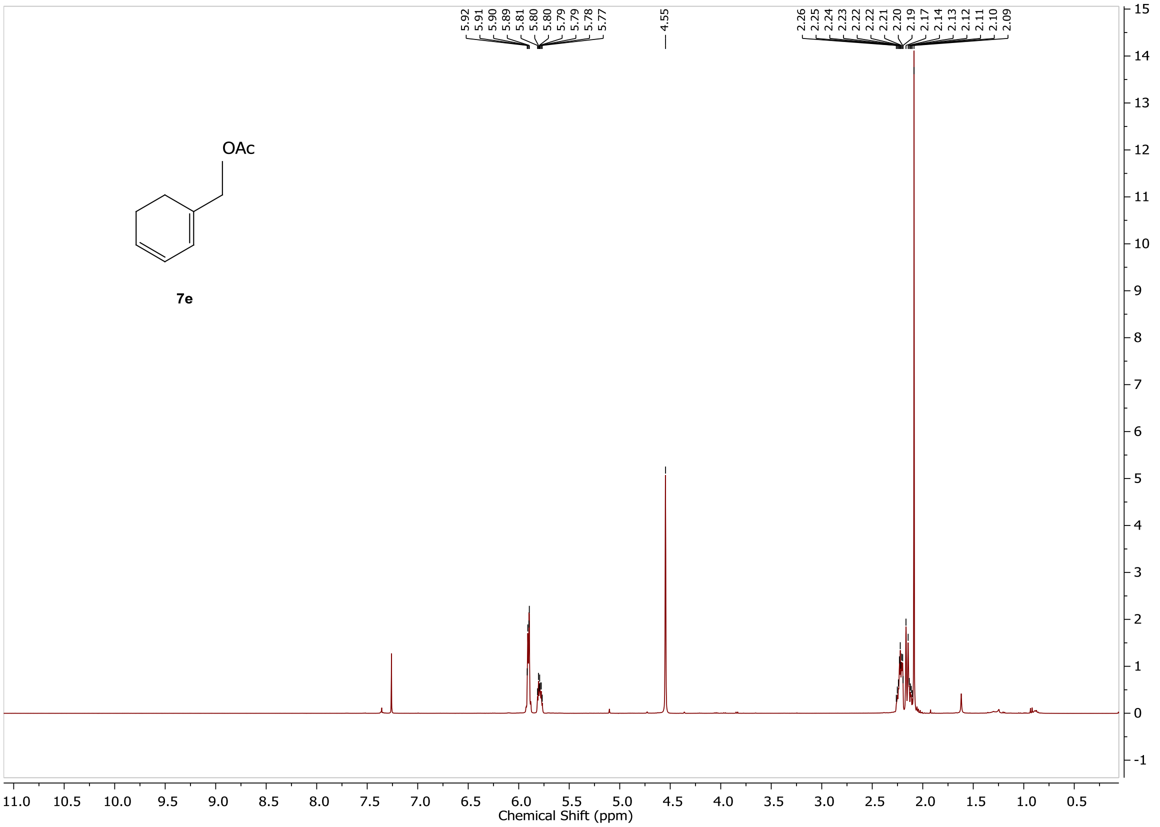

5.92
5.91
5.90
5.89
5.81
5.80
5.80
5.79
5.79
5.78
5.77
4.55
2.26
2.25
2.24
2.23
2.22
2.22
2.21
2.20
2.19
2.17
2.14
2.13
2.12
2.11
2.10
2.09
OAc
7e
Chemical Shift (ppm)

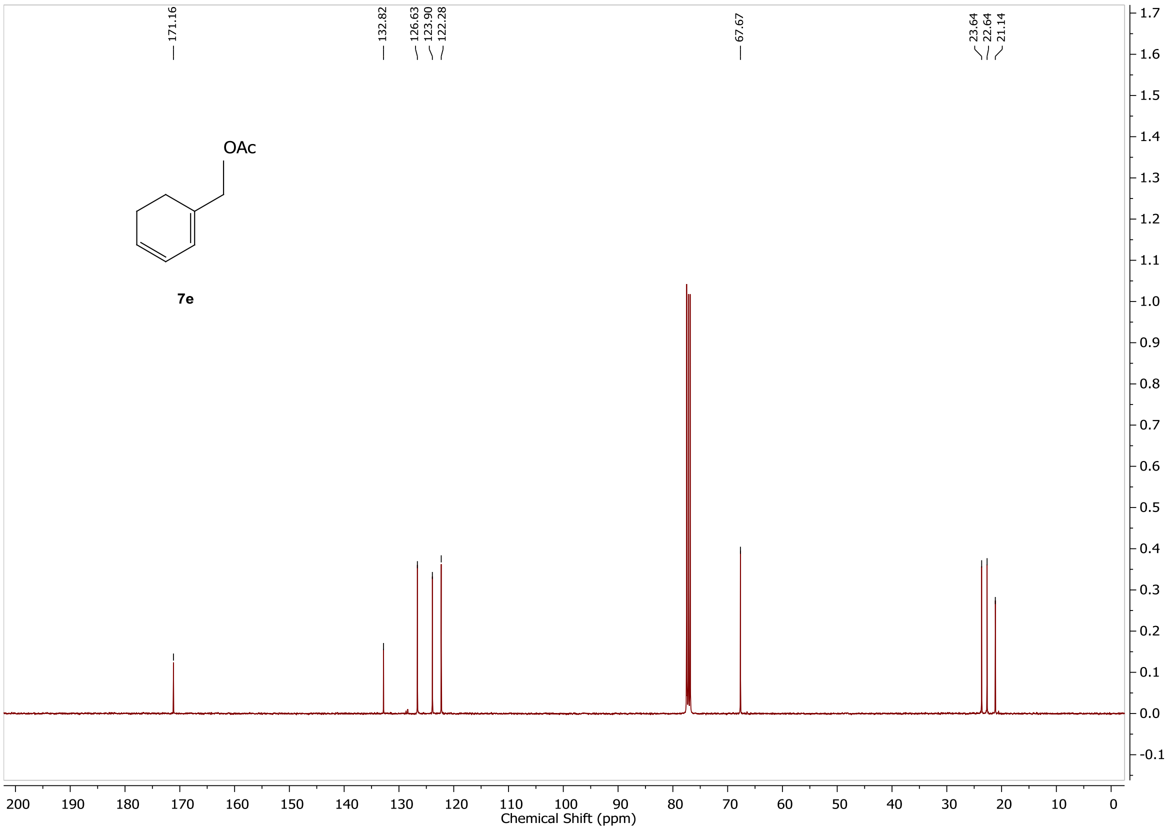

171.16
132.82
126.63
123.90
122.28
67.67
23.64
22.64
21.14
OAc
7e
Chemical Shift (ppm)

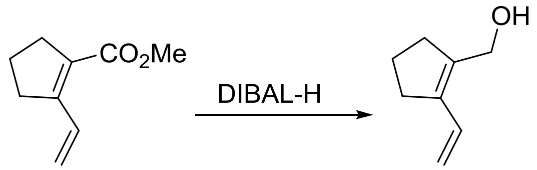

S7 **S15**

(2-Vinylcyclopent-1-en-1-yl)methanol (S15)

DIBAL-H in toluene (1.2 M, 1.0 mL, 1.2 mmol) was added to ester **S7** (88 mg, 0.58 mmol) in THF (5.8 mL) at −78 °C. The reaction was warmed to room temperature. After stirring for 4 hours the reaction mixture was quenched first with $Na_2SO_4 \cdot 10H_2O$ until bubbling ceased and then with saturated NH_4Cl solution, extracted with diethyl ether, and dried using Na_2SO_4. The solution was concentrated and purified via silica gel chromatography (80:20, pentane/diethyl ether) to give alcohol **S15** (53 mg, 75%) as a yellow oil. R_f = 0.31 (80:20, hexanes/EtOAc).

^{1}H NMR (500 MHz, $CDCl_3$) δ 6.70 (dd, *J* = 17.2, 10.7 Hz, 1H), 5.14 – 5.10 (m, 2H), 4.31 (s, 2H), 2.56 (t, *J* = 7.4 Hz, 2H), 2.52 (t, *J* = 7.6 Hz, 2H), 1.87 (quint, *J* = 7.5 Hz, 2H) ppm.

^{13}C NMR (125 MHz, $CDCl_3$) δ 140.3, 137.5, 130.3, 115.0, 58.8, 35.2, 32.8, 21.5 ppm.

HRMS (ESI) calcd. for $[C_8H_{12}O+H]^+$: 125.1901, found: 125.0966.

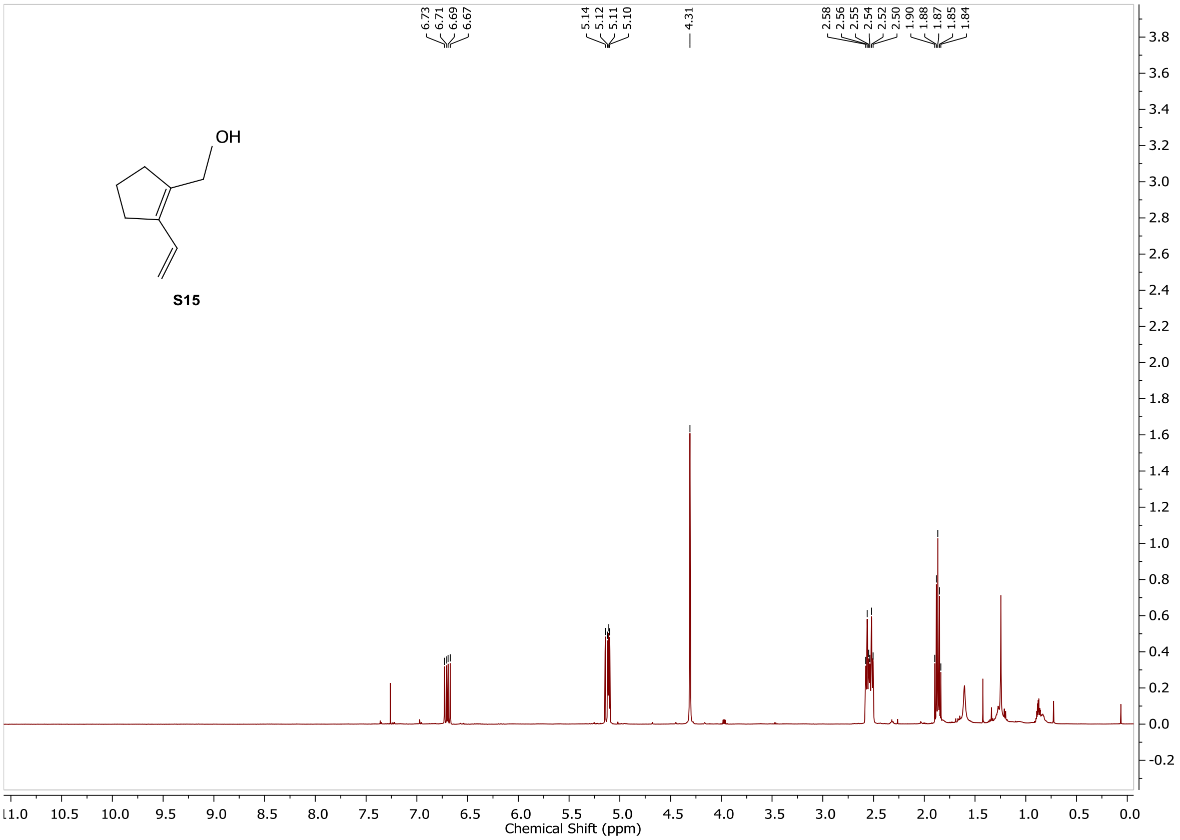
6.73
6.71
6.69
6.67
5.14
5.12
5.11
5.10
4.31
2.58
2.56
2.55
2.54
2.52
2.50
1.90
1.88
1.87
1.85
1.84
OH
S15
Chemical Shift (ppm)

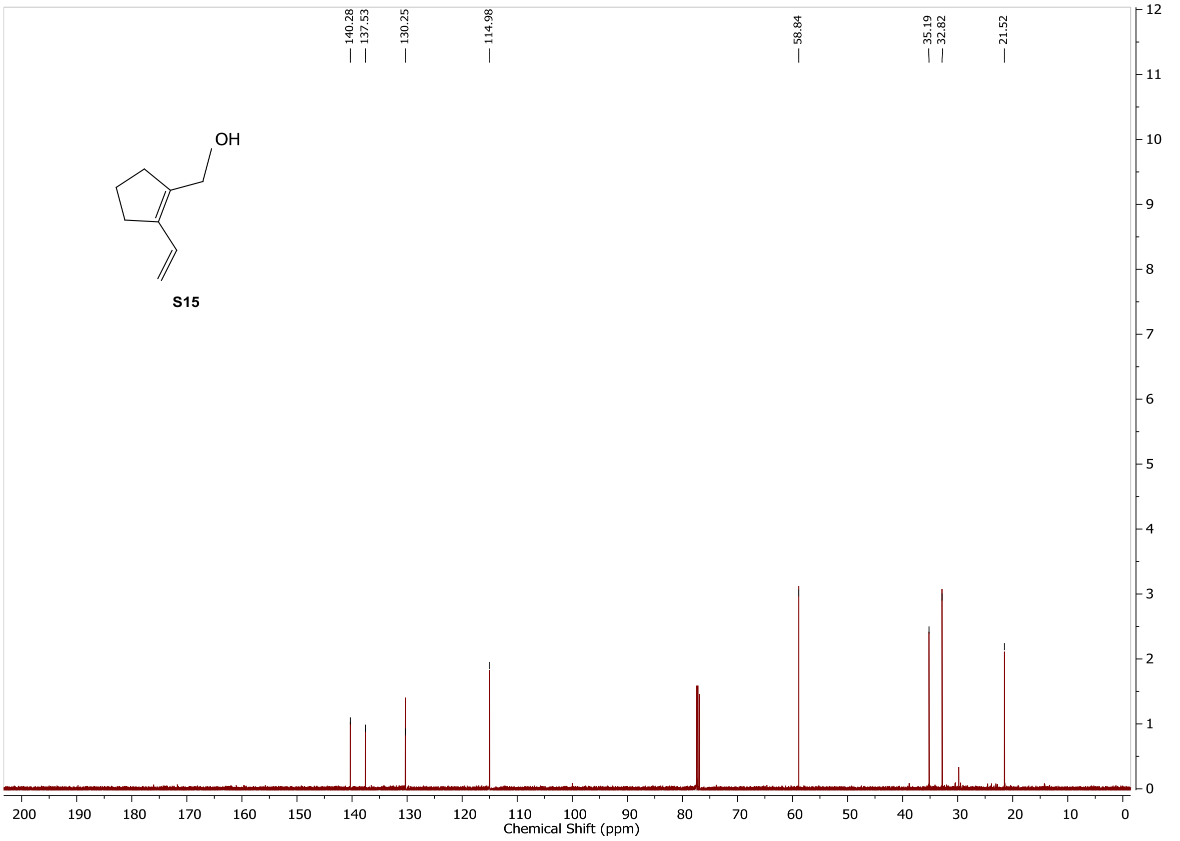
140.28
137.53
130.25
114.98
58.84
35.19
32.82
21.52
OH
S15
Chemical Shift (ppm)

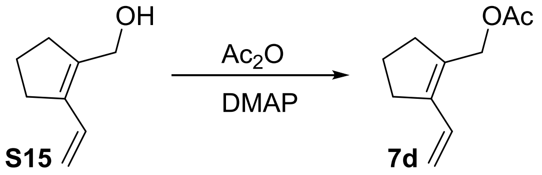

(2-Vinylcyclopent-1-en-1-yl)methyl acetate (7d)

To a solution of alcohol **S15** (20 mg, 0.16 mmol) and triethylamine (45µL, 0.32 mmol) in CH_2Cl_2 (1.6 mL) was added acetic anhydride (19 µL, 0.19 mmol) and DMAP (2.0 mg, 0.016 mmol) at 0 °C. After stirring for an hour at room temperature, the reaction was diluted with diethyl ether and washed with water. The aqueous layer was back extracted with diethyl ether, and the combined organic layers dried using Na_2SO_4 and concentrated. Purification via silica gel chromatography (95:5, pentane/diethyl ether) yielded acetate **7d** (15 mg, 56%) as a yellow oil. R_f = 0.76 (80:20, hexanes/EtOAc).

^{1}H NMR (500 MHz, $CDCl_3$) δ 6.70 (dd, *J* = 16.8, 11.1 Hz, 1H), 5.17 (d, *J* = 16.7 Hz, 1H), 5.16 (d, 11.0 Hz, 1H), 4.76 (s, 2H), 2.55 – 2.50 (m, 4H), 2.06 (s, 3H), 1.88 (quint, *J* = 7.6 Hz, 2H) ppm.

^{13}C NMR (125 MHz, $CDCl_3$) δ 171.2, 139.8, 135.3, 130.0, 115.8, 60.4, 35.5, 32.7, 21.4, 21.0 ppm.

HRMS (ESI) calcd. for $[C_{10}H_{14}O_2+H]^+$: 167.2273, found: 167.1072.

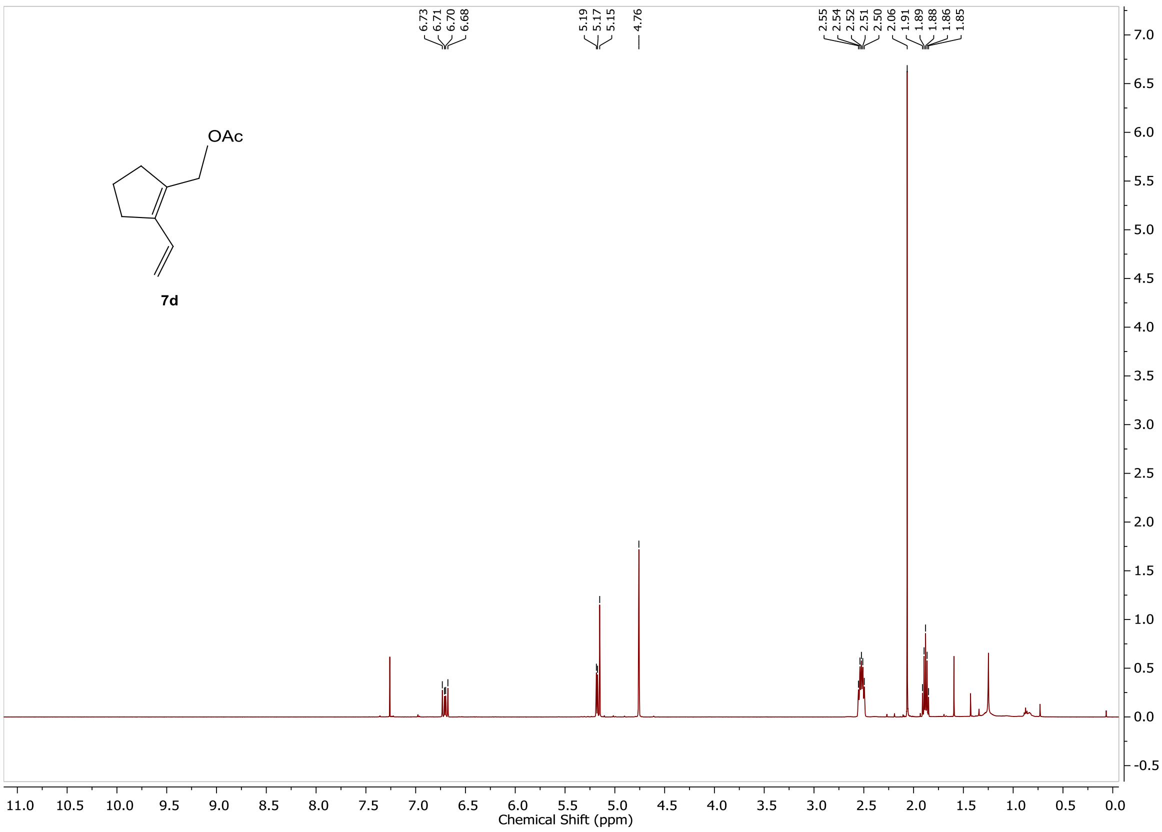
6.73
6.71
6.70
6.68
5.19
5.17
5.15
4.76
2.55
2.54
2.52
2.51
2.50
2.06
1.91
1.89
1.88
1.86
1.85
OAc
7d
Chemical Shift (ppm)

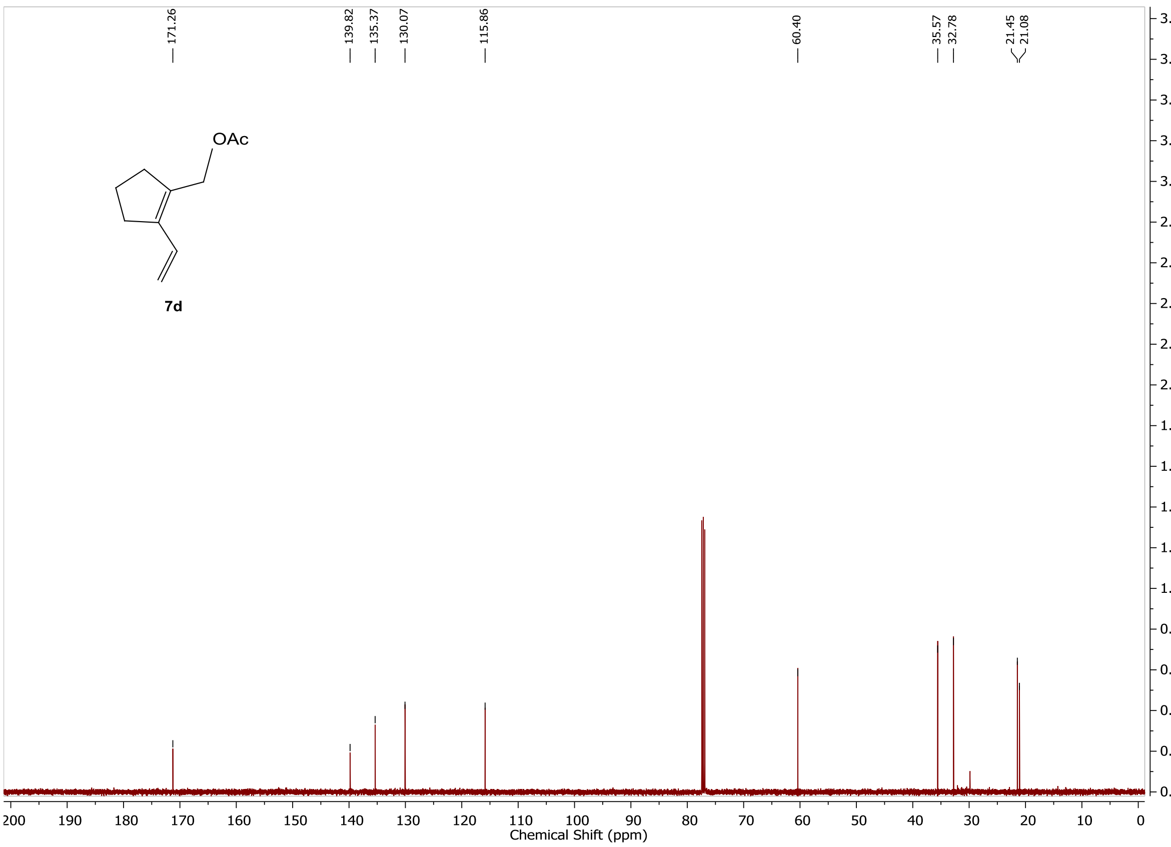
171.26
139.82
135.37
130.07
115.86
60.40
35.57
32.78
21.45
21.08
OAc
7d
Chemical Shift (ppm)

Experimental procedures for single component decarboxylative coupling

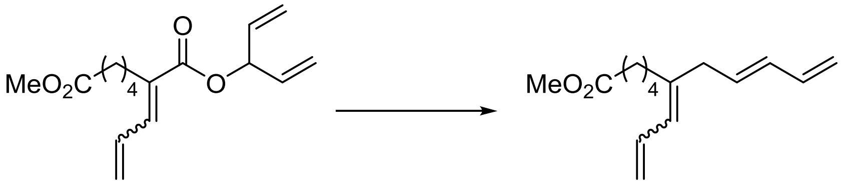

9 10

(6*E*/*Z*,8*E*)-Methyl 6-allylideneundeca-8,10-dienoate (10)

To a microwave vial was added diene **9** (40 mg, 0.14 mmol)[xii] and water (2.7 µL, 0.15 mmol) in CH_2Cl_2 (2 mL). Tetrakis-(triphenylphosphine) palladium (16 mg, 0.014 mmol) was added and the vial was sealed and purged with N_2. The mixture was a bright orange color. After 24 hours at room temperature, the mixture was a turbid yellow color. The reaction was concentrated and purified via silica gel chromatography (97:3, hexanes/EtOAc) to yield tetraene **10** (22 mg, 76%) as a yellow oil. Scale-up beyond 100 mg resulted in decreased yields; however, when eight vials were run simultaneously and purified together, the yield remained around 70%. R_f = 0.90 (90:10, hexanes/EtOAc). Spectral data matched those previously reported.[xii]

E Diastereomer:

^{1}H NMR (400 MHz, $CDCl_3$) δ 6.71 – 6.52 (m, 2H), 6.11 (t, *J* = 11.6 Hz, 1H), 5.87 (d, *J* = 11.0 Hz, 1H), 5.44 (q, *J* = 8.2 Hz, 1H), 5.23 (dd, *J* = 16.9, 1.9 Hz, 1H), 5.13 (ddd, *J* = 16.5, 5.8, 2.1 Hz, 2H), 5.02 (ddd, *J* = 10.0, 6.1, 2.0 Hz, 1H), 3.67 (s, 3H), 2.93 (d, *J* = 7.7 Hz, 2H), 2.32 (td, J_t = 7.5, J_d = 3.3 Hz, 2H), 2.18 (t, *J* = 7.8 Hz, 2H), 1.74 – 1.61 (m, 2H), 1.47 – 1.40 (m, 2H) ppm.

Z Diastereomer:

^{1}H NMR (400 MHz, $CDCl_3$) δ 6.72 – 6.50 (m, 2H), 6.03 (t, *J* = 11.15 Hz, 1H), 5.87 (d, *J* = 10.9 Hz, 1H), 5.34 (q, *J* = 7.5 Hz, 1H), 5.23 (dd, *J* = 1.4, 16.9 Hz, 1H), 5.16 – 5.08 (m, 2H), 5.02 (m, 1H), 3.67 (s, 3H), 3.06 (d, *J* = 6.9 Hz, 2H), 2.32 (m, 2H), 2.06 (t, *J* = 7.4 Hz, 2H), 1.65 – 1.60 (m, 2H), 1.47 – 1.40 (m, 2H) ppm.

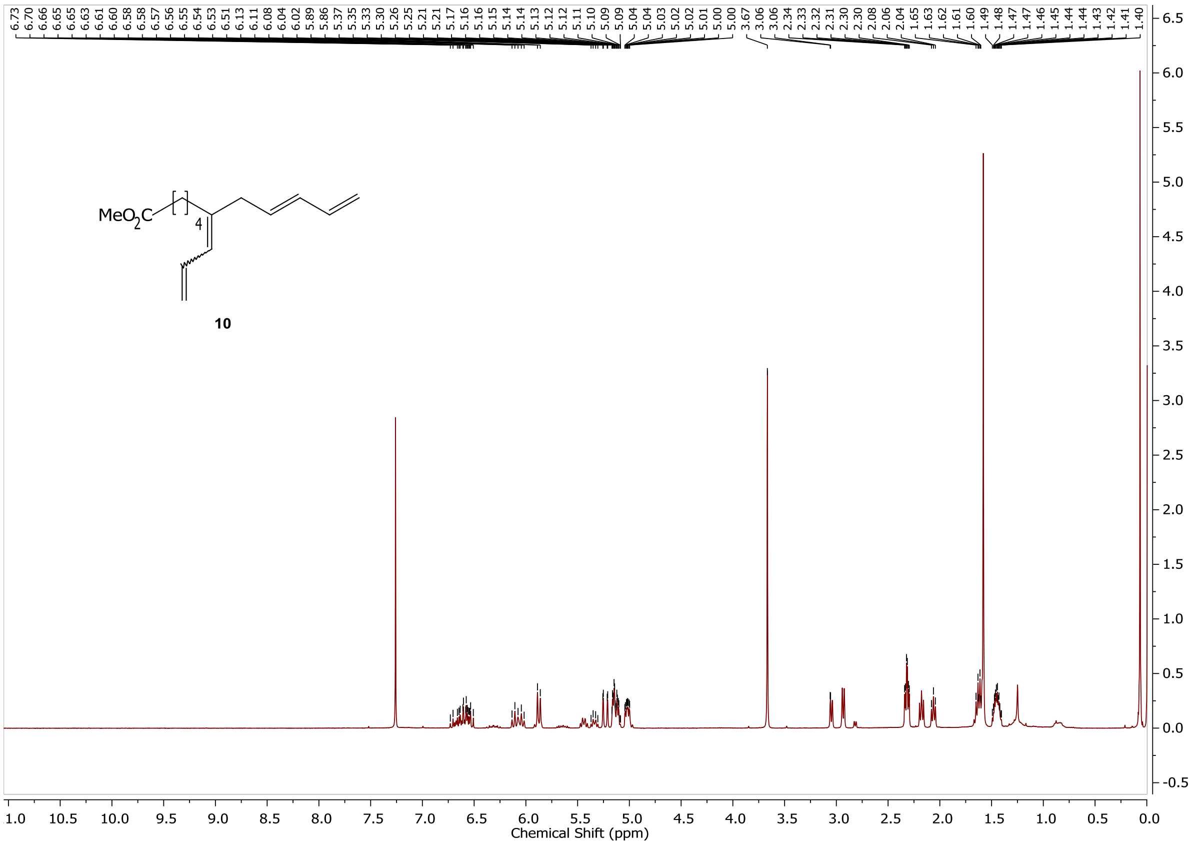

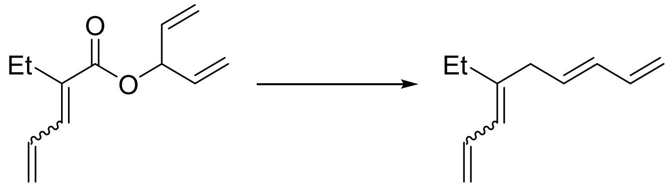

11 **12**

(6*E*)-4-Ethylnona-1,3,6,8-tetraene (12)

Dienoate **11** (40 mg, 0.17 mmol)[xii] was added to a small vial with CH_2Cl_2 (2 mL) after which tetrakis-(triphenylphosphine) palladium (19.7 mg, 0.017 mmol) was added. The vial was sealed and purged with N_2. When initially prepared, the solution was a dark orange color, but after 24 hours, it was a light yellow color. At this time, the reaction was concentrated and purified via silica gel chromatography (hexanes) to yield ethyl tetraene **12**. The yield was inconsistent due to the extreme volatility of the product which made removing solvent difficult.[xii]

Diastereomer A:

^{1}H NMR (500 MHz, $CDCl_3$) δ 7.18 (d, *J* = 11.5 Hz, 1H), 6.68 (ddd, *J* = 10.3, 11.3, 16.8 Hz, 1H), 5.89 (ddd, *J* = 5.8, 10.3, 16.6 Hz, 2H), 5.84 – 5.78 (m, 1H), 5.59 (dd, *J* = 1.1, 16.6 Hz, 1H), 5.47 (dd, *J* = 1.1, 10.3 Hz, 1H), 5.37 – 5.31 (m, 2H), 5.27 – 5.23 (m, 2H), 2.46 (q, *J* = 7.4 Hz, 2H), 1.06 (t, *J* = 7.4 Hz, 3H) ppm.

Diastereomer B:

¹H NMR (500 MHz, $CDCl_3$) δ 7.31 – 7.22 (m, 1H), 6.38 (d, *J* = 11.5 Hz, 1H), 5.89 (ddd, *J* = 5.8, 10.3, 16.6 Hz, 2H), 5.84 – 5.78 (m, 1H), 5.40 (dd, *J* = 1.7, 17.1 Hz, 1H), 5.37 – 5.31 (m, 3H), 5.27 – 5.23 (m, 2H), 2.38 (q, *J* = 7.4 Hz, 2H), 1.09 (t, *J* = 7.5 Hz, 3H) ppm.

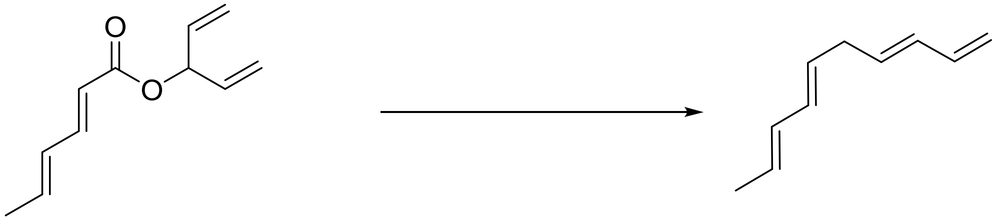

13 **8a**

(6*E*,8*E*)-Deca-1,3,6,8-tetraene (8a)

To a microwave vial with dienoate **13** (11 mg, 0.063 mmol) and water (1.25 µL, 0.07 mmol) in $CDCl_3$ (1.5 mL) was added tetrakis-(triphenylphosphine) palladium (7.3 mg, 0.0063 mmol) and the vial was sealed and purged with N_2. The mixture was stirred at room temperature for 24 hours and then transferred to a vial containing dimethylterphthalate (2.6 mg, internal standard). Quantitative ¹H NMR analysis of this mixture shows the formation of tetraene **8a** (5%, using Equation 1) in addition to the rearranged ester **20**. The spectral data for (6*E*,8*E*)-deca-1,3,6,8-tetraene, **8a** is provided below for the two component reaction.

$$^{1}\text{H-NMR Yield} = \frac{\left(\left[\dfrac{\text{area under the curve}_{(sample)}}{\text{\# of protons}}\right] \Big/ \left[\dfrac{\text{area under the curve}_{(IS)}}{\text{\# of protons}}\right]\right)\left(\dfrac{\text{weight}_{(IS)}\text{ added}}{\text{MW}_{(IS)}}\right)}{\text{Theoretical mmols}} \times 100 \qquad (\mathbf{1})$$

[See compound **8h**/**8i**/**8j** for an example using Equation 1.]

Experimental procedures for two component decarboxylative coupling

***General Procedure*:** A microwave vial with dienoic acid **5** (1.0 equiv), pentadienyl substrate **6** (1.2 equiv) and water (1.1 equiv) in $CDCl_3$ (0.1 M) was capped with a septum, and purged with N_2. Tris(dibenzylideneacetone)dipalladium(0)-chloroform adduct (5 mol %) and PPh_3 (20%) were dissolved in $CDCl_3$ (0.1 mL) and added to the mixture. The mixture was left at room temperature under a balloon of N_2 for 48 hours. The solution was concentrated and purified via silica gel chromatography using pentane.

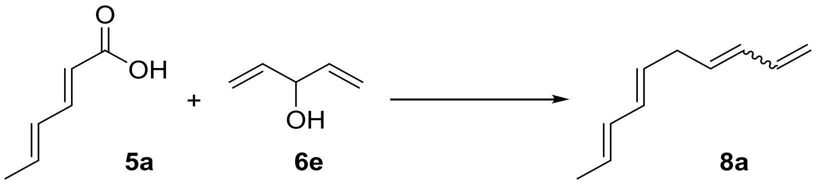

(6*E*,8*E*)-Deca-1,3,6,8-tetraene (8a)

Following the general procedure, tetraene **8a** was synthesized as a colorless solution in $CDCl_3$ (40% ^{1}H NMR yield). R_f (mixture of diastereomers) = 0.78 (hexanes). The product is a mixture of two diastereomers in a 10:1 ratio. Only the major, all-*E* diastereomer, was fully characterized.

^{1}H NMR (500 MHz, $CDCl_3$) δ 6.63 (dddd, *J* = 16.9, 11.2, 10.2, 1.1 Hz, 1H), 6.07 – 5.99 (m, 3H), 5.64 – 5.58 (m, 1H), 5.57 – 5.50 (m, 1H), 5.47 – 5.42 (m, 1H), 5.23 (dd, *J* = 16.8, 2.0 Hz, 1H), 5.12 (d, *J* = 10.2 Hz, 1H), 3.07 (t, *J* = 7.5 Hz, C*H_2 minor isomer*), 2.95 (t, *J* = 7.1 Hz, 2H), 1.73 (d, *J* = 6.0 Hz, 3H) ppm.

^{13}C NMR (125 MHz, $CDCl_3$) δ 132.1, 131.4, 131.1, 129.9, 129.8, 129.1, 127.8, 117.7, 30.8, 18.2 ppm.

GC-LR-MS (EI 70 eV) m/z (%) calcd. for $C_{10}H_{14}$: 134, found: 134.

Attempts to obtain HRMS data using ESI and API were unsuccessful.

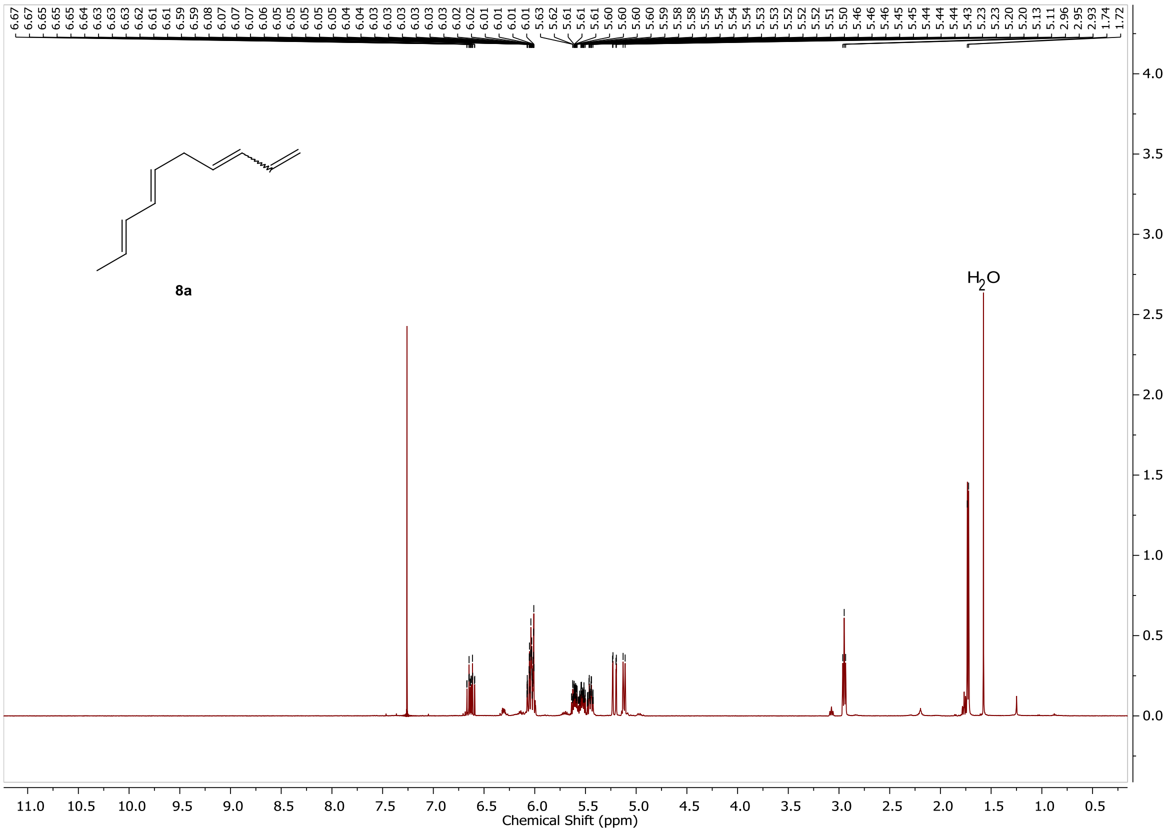

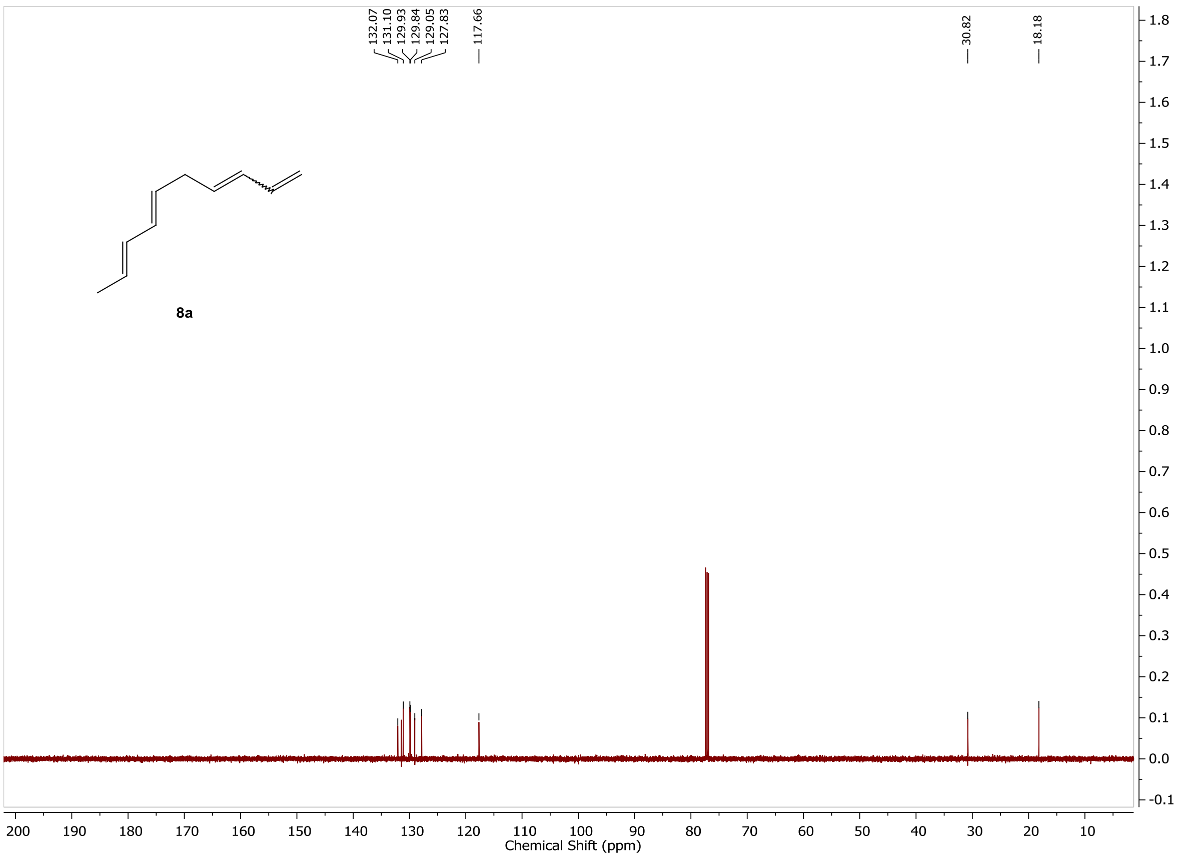

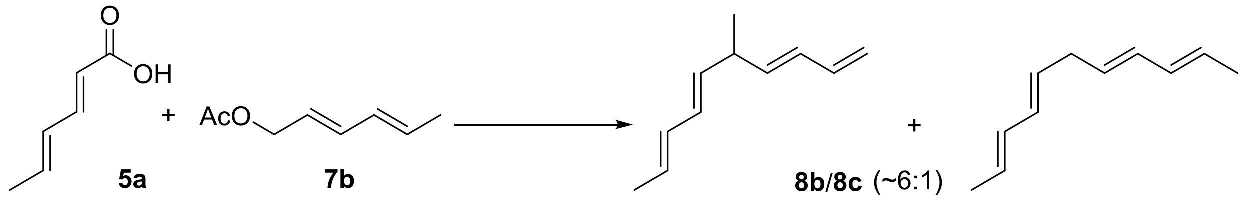

5-Methyldeca-1,3,6,8-tetraene (8b)

Following the general procedure, tetraene **8b** was synthesized as a colorless oil (3.4 mg, 8% ^{1}H NMR yield). R_f = 0.63 (hexanes). The product is a mixture of two isomers **8b**/**8c** in a 6:1 ratio. Only the major isomer **8b** was fully characterized.

^{1}H NMR (400 MHz, $CDCl_3$) δ 6.68 – 6.58 (m, 1H), 6.06 – 5.92 (m, 3H), 5.68 – 5.55 (m, 1H), 5.49 (dd, *J* = 14.3, 6.3 Hz, 1H), 5.27 (t, *J* = 10 Hz, 1H), 5.19 (dd, *J* = 16.8, 1.9 Hz, 1H), 5.10 (td, J_d = 9.9, J_t = 2.0 Hz, 1H), 3.35 (dq, J_q = 16.0, J_d = 6.8, 1H), 2.91 (t, *J* = 6.7 Hz, CH_2 *minor isomer* **8c**), 1.73 (d, *J* = 6.6 Hz, 3H), 1.08 (d, *J* = 6.8 Hz, 3H) ppm.

^{13}C NMR (100 MHz, $CDCl_3$) δ 136.3, 135.4, 132.3, 131.6, 128.8, 128.1, 127.9, 117.6, 34.9, 21.0, 18.2 ppm.

GC-LR-MS (EI 70 eV) m/z (%) calcd. for $C_{11}H_{16}$: 148, found: 148.

Attempts to obtain HRMS data using ESI and API were unsuccessful.

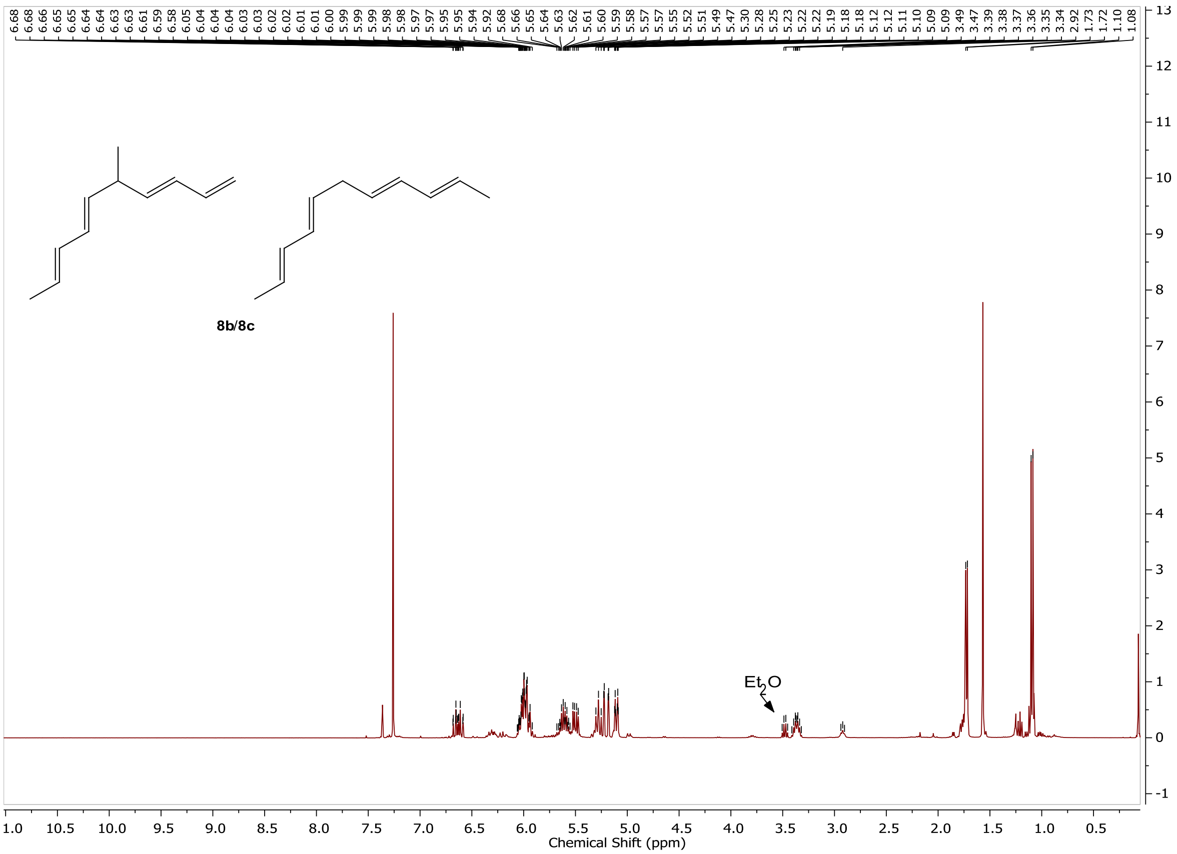
6.68 6.68 6.66 6.65 6.65 6.64 6.64 6.63 6.63 6.61 6.59 6.58 6.05 6.04 6.04 6.04 6.03 6.03 6.02 6.02 6.01 6.01 6.00 5.99 5.99 5.99 5.99 5.98 5.98 5.97 5.97 5.95 5.95 5.94 5.92 5.68 5.66 5.65 5.64 5.63 5.62 5.61 5.60 5.59 5.58 5.57 5.57 5.55 5.52 5.51 5.49 5.47 5.30 5.28 5.25 5.23 5.22 5.22 5.19 5.18 5.18 5.12 5.12 5.11 5.10 5.09 5.09 3.49 3.47 3.39 3.38 3.37 3.36 3.35 3.34 2.92 1.73 1.72 1.10 1.08
8b/8c
Et2O
Chemical Shift (ppm)

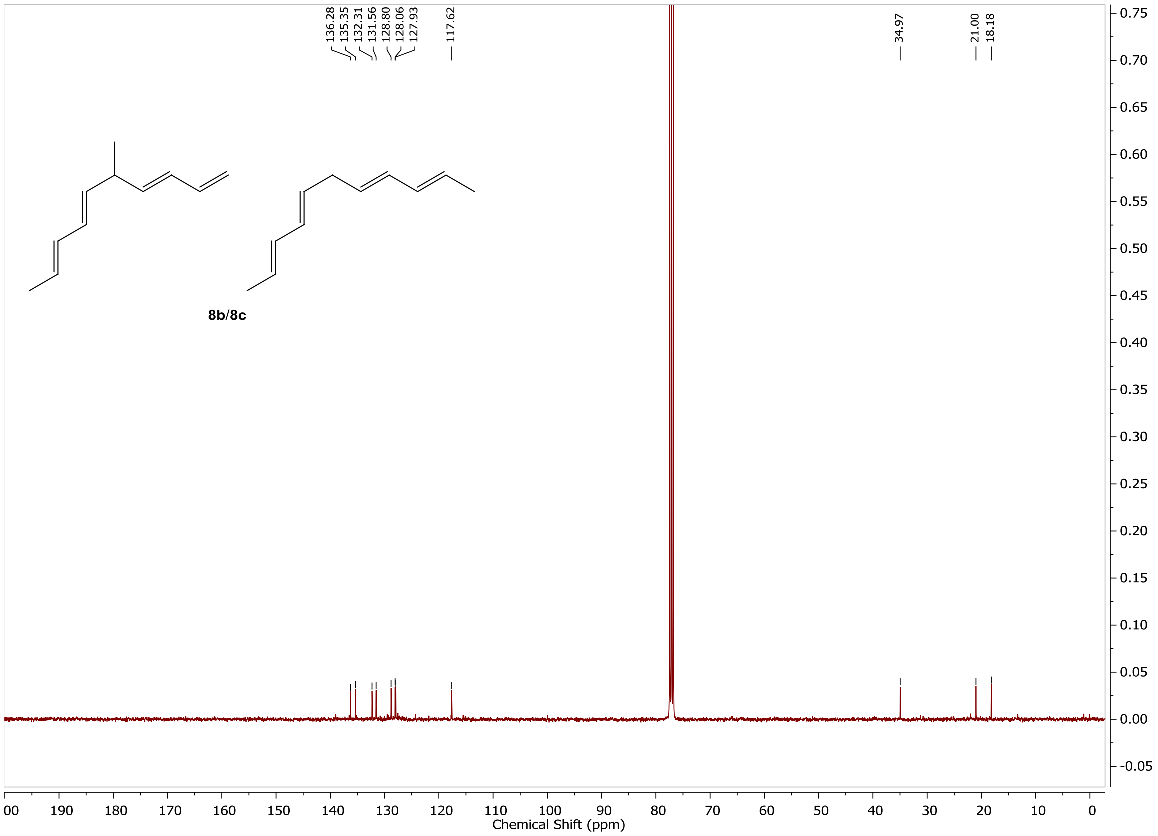
136.28 135.35 132.31 131.56 128.80 128.06 127.93 117.62
34.97
21.00 18.18
8b/8c
Chemical Shift (ppm)

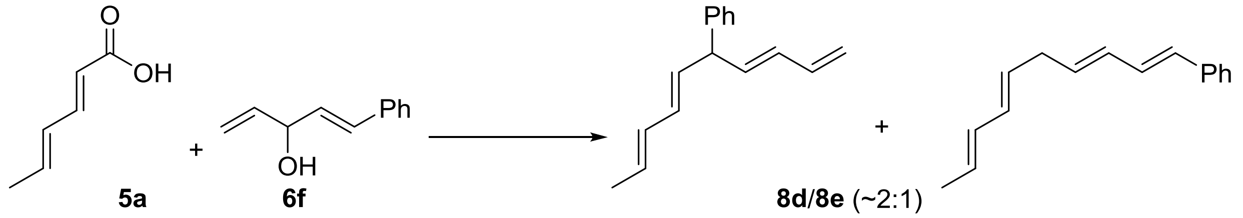

(Deca-1,3,6,8-tetraen-5-yl)benzene (8d)

Following the general procedure, tetraene **8d** was synthesized as a colorless oil, (6.3 mg, 17%, isolated yield). R_f = 0.37 (hexanes). The product is a mixture of two isomers **8d**/**8e** in a 2:1 ratio. Only the major isomer **8d** was fully characterized.

^{1}H NMR (400 MHz, $CDCl_3$) δ 7.45 – 7.38 (m, 1H), 7.35 – 7.27 (m, 2H), 7.25 – 7.15 (m, 2H), 6.73 (dt, J_d = 16.9, J_t = 10.6 Hz, 1H), 6.12 – 6.04 (m, 3H), 5.74 – 5.49 (m, 3H), 5.27 (dd, *J* = 16.9, 9.2 Hz, 1H), 5.17 (dd, *J* = 10.4, 1.9 Hz, 1H), 4.54 (dd, *J* = 9.9, 6.5 Hz, 1H, C*H benzylic* major isomer **8d**), 3.05 (t, *J* = 7.2 Hz, 2H, CH_2 *minor isomer* **8e**), 1.74 (d, *J* = 6.1 Hz, 3H, *major isomer* **8d**) 1.73 (d, *J* = 6.1 Hz, 3H, CH_3 *minor isomer* **8e**) ppm.

^{13}C NMR (100 MHz, $CDCl_3$) δ 133.2, 132.7, 132.0, 131.3, 131.0, 129.2, 128.8, 128.7, 128.6 (2C), 127.7 (2C), 126.5, 118.7, 46.2, 18.2 ppm.

GC-LR-MS (EI 70 eV) m/z (%) calcd. for $C_{16}H_{18}$: 210, found: 210.

Attempts to obtain HRMS data using ESI and API were unsuccessful.

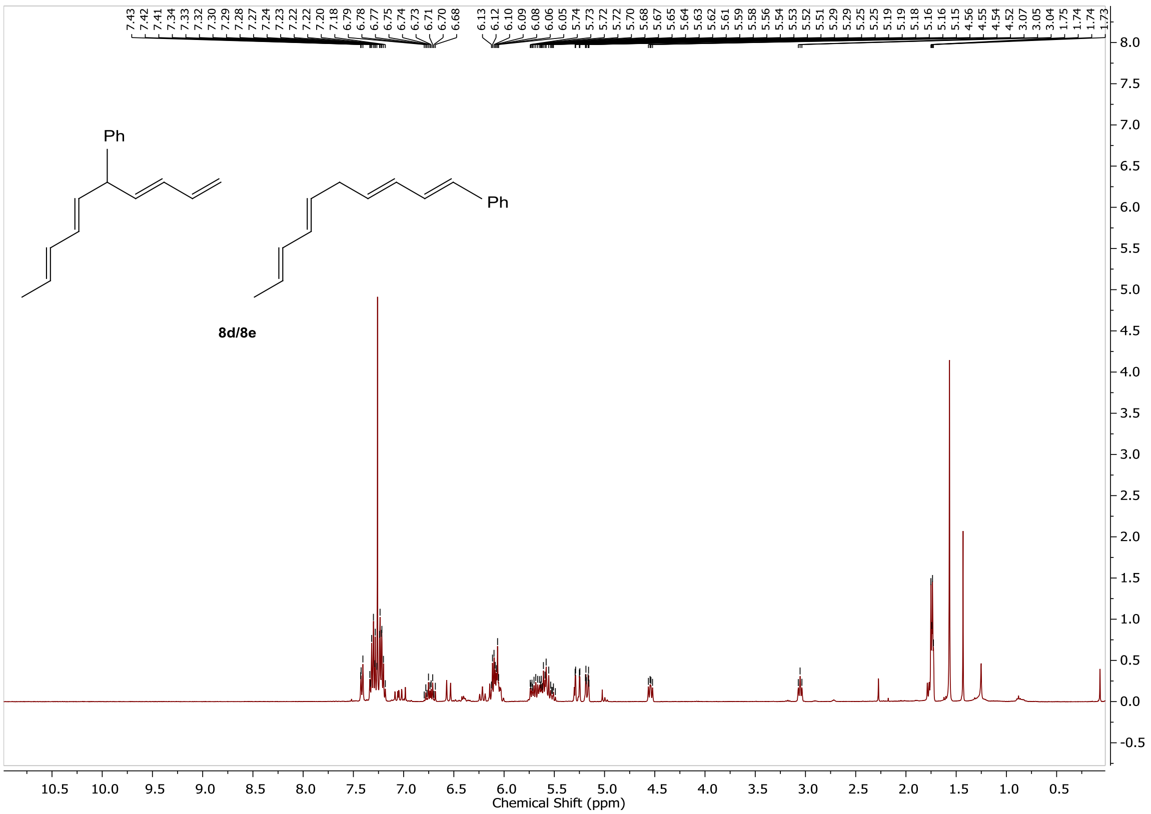
7.43 7.42 7.41 7.34 7.33 7.32 7.30 7.29 7.28 7.27 7.24 7.23 7.22 7.22 7.20 7.18 6.79 6.78 6.77 6.75 6.74 6.73 6.71 6.70 6.68
6.13 6.12 6.10 6.09 6.08 6.06 6.05 5.74 5.73 5.72 5.72 5.70 5.68 5.67 5.65 5.64 5.63 5.62 5.61 5.59 5.58 5.56 5.54 5.53 5.52 5.51 5.29 5.29 5.25 5.25 5.19 5.19 5.18 5.16 5.16 5.15 4.56 4.55 4.54 4.52 3.07 3.05 3.04 1.75 1.74 1.74 1.73
Ph
Ph
8d/8e
Chemical Shift (ppm)

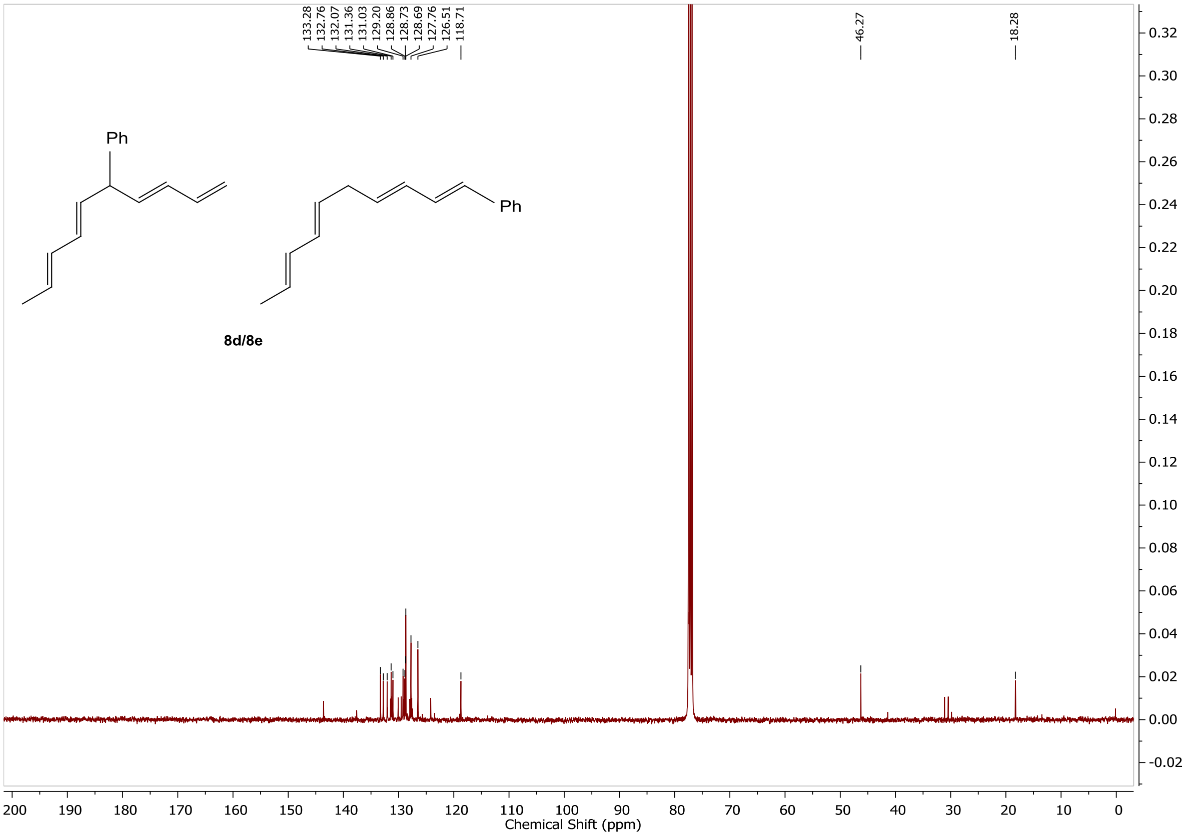
133.28 132.76 132.07 131.36 131.03 129.20 128.86 128.73 128.69 127.76 126.51 118.71
46.27
18.28
Ph
Ph
8d/8e
Chemical Shift (ppm)

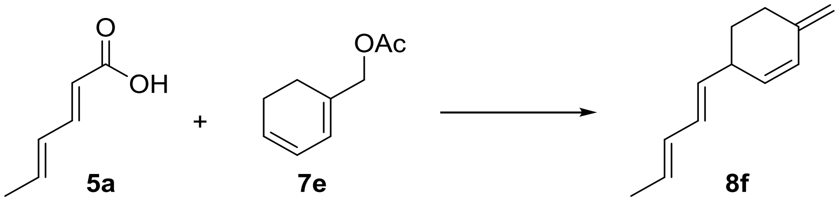

3-Methylene-6-((1*E*,3*E*)-penta-1,3-dien-1-yl)cyclohex-1-ene (8f)

Following the general procedure, tetraene **8f** was synthesized as a light yellow oil, (3.5 mg, 16%, isolated yield). R_f = 0.96 (90:10, hexanes/EtOAc).

^{1}H NMR (400 MHz, $CDCl_3$) δ 6.17 (dd, *J* = 10.0, 2.2 Hz, 1H), 6.05 – 5.99 (m, 2H), 5.69 (dd, *J* = 9.7, 3.7 Hz, 1H), 5.65 – 5.60 (m, 1H), 5.55 – 5.49 (m, 1H), 4.80 (s, 1H), 4.77 (s, 1H), 2.94 – 2.88 (m, 1H), 2.44 – 2.35 (m, 1H), 2.34 – 2.25 (m, 1H), 1.87 (ddt, J_t = 12.6, J_d = 7.5, J_d = 4.5 Hz, 1H), 1.74 (d, *J* = 6.5 Hz, 3H), 1.56 – 1.47 (m, 1H) ppm.

^{13}C NMR (125 MHz, $CDCl_3$) δ 143.0, 134.3, 132.7, 131.5, 130.3, 129.6, 128.1, 111.2, 38.5, 29.5, 28.8, 18.3 ppm.

HRMS (APPI) calcd. for $[C_{12}H_{16}]^+$: 160.1246, found: 160.1245.

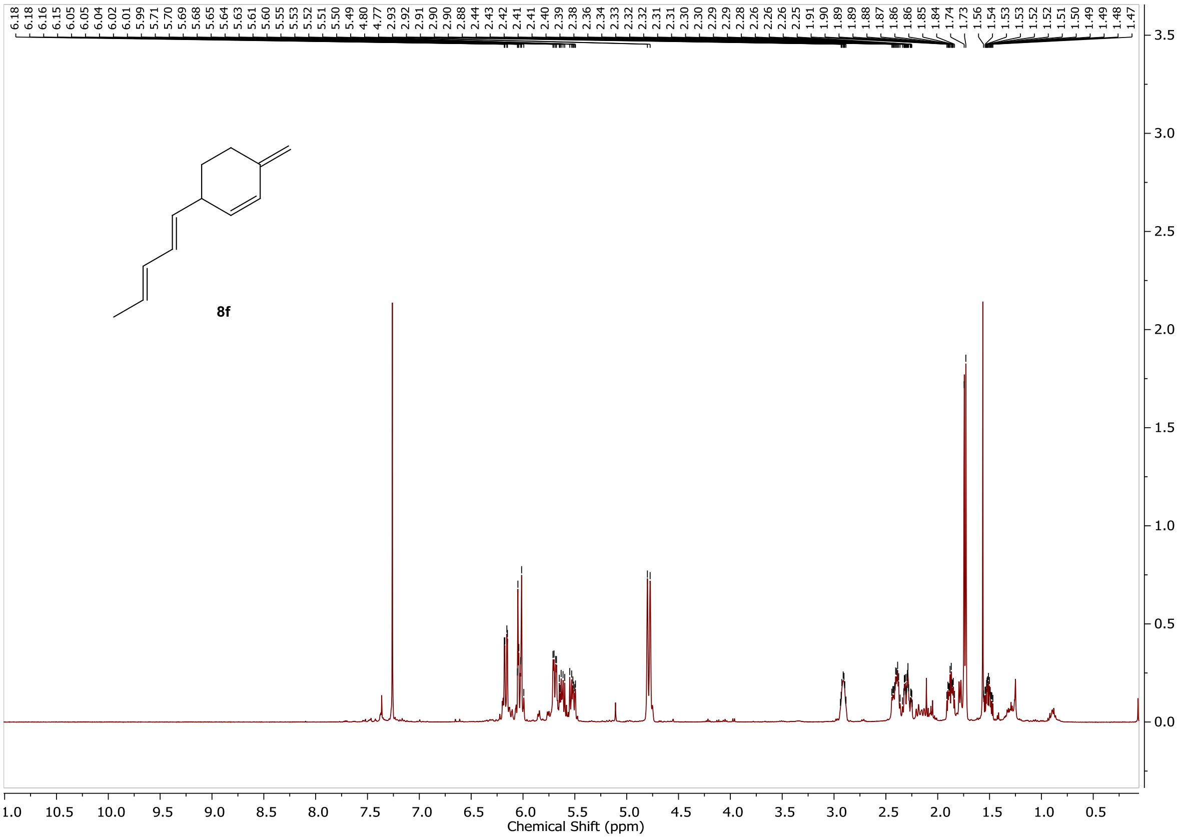

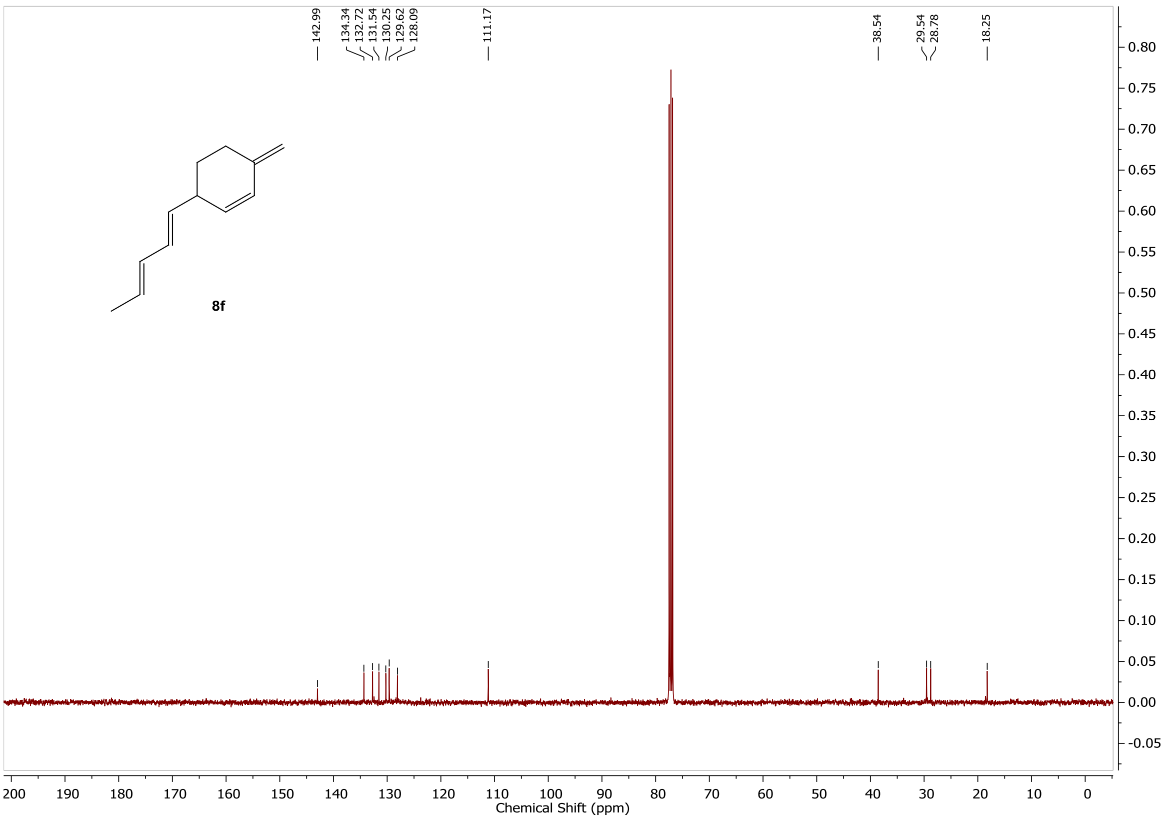

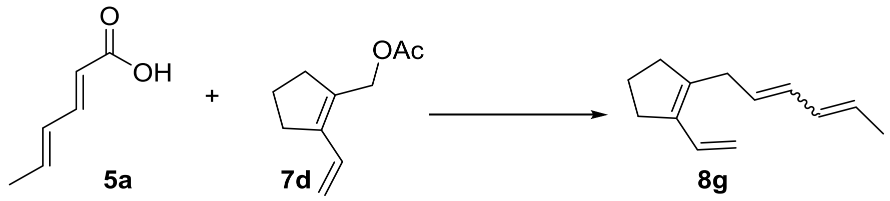

1-(Hexa-2,4-dien-1-yl)-2-vinylcyclopent-1-ene (8g)

Following the general procedure, tetraene **8g** was synthesized as a colorless oil, (2.0 mg, 18%, isolated yield). R_f = 0.96 (90:10, hexanes/EtOAc). The product is a mixture of two diastereomers in a 2:1 ratio. Only the major diastereomer was fully characterized.

^{1}H NMR (500 MHz, $CDCl_3$) δ 6.67 (dd, *J* = 16.8, 11.0 Hz, 1H), 6.06 – 5.95 (m, 2H), 5.63 – 5.55 (m, 1H), 5.53 – 5.45 (m, 1H), 5.09 – 5.02 (m, 2H), 2.96 (d, *J* = 6.9 Hz, 2H), 2.50 – 2.47 (m, 2H), 2.42 – 2.39 (m, 2H), 1.85 – 1.79 (m, 2H), 1.72 (d, *J* = 6.5 Hz, 3H) ppm.

^{13}C NMR (125 MHz, $CDCl_3$) δ 140.7, 131.5, 131.4, 131.0, 130.9, 128.7, 127.7, 113.3, 37.1, 32.6, 31.8, 21.5, 18.2 ppm.

HRMS (APPI) calcd. for $[C_{13}H_{18}+H]^+$: 175.1487, found: 175.1480.

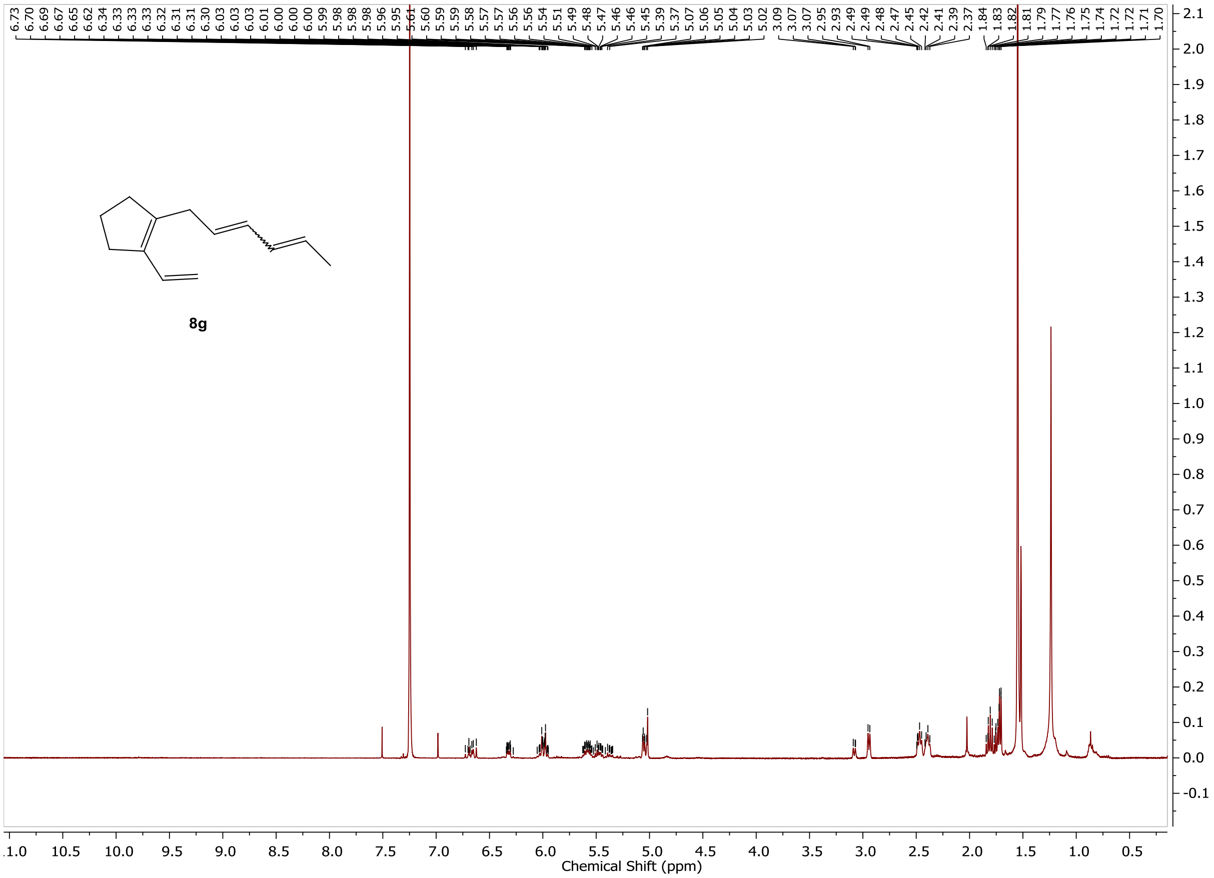
6.73 6.70 6.69 6.67 6.65 6.62 6.34 6.33 6.33 6.33 6.32 6.31 6.31 6.30 6.03 6.03 6.03 6.03 6.01 6.00 6.00 6.00 5.99 5.98 5.98 5.98 5.96 5.95 5.61 5.60 5.59 5.59 5.58 5.57 5.57 5.56 5.56 5.54 5.51 5.49 5.48 5.47 5.46 5.46 5.45 5.39 5.37 5.07 5.06 5.05 5.04 5.03 5.02 3.09 3.07 3.07 2.95 2.93 2.49 2.49 2.48 2.47 2.45 2.42 2.41 2.39 2.37 1.84 1.83 1.82 1.81 1.79 1.77 1.76 1.75 1.74 1.72 1.72 1.71 1.70
8g
Chemical Shift (ppm)

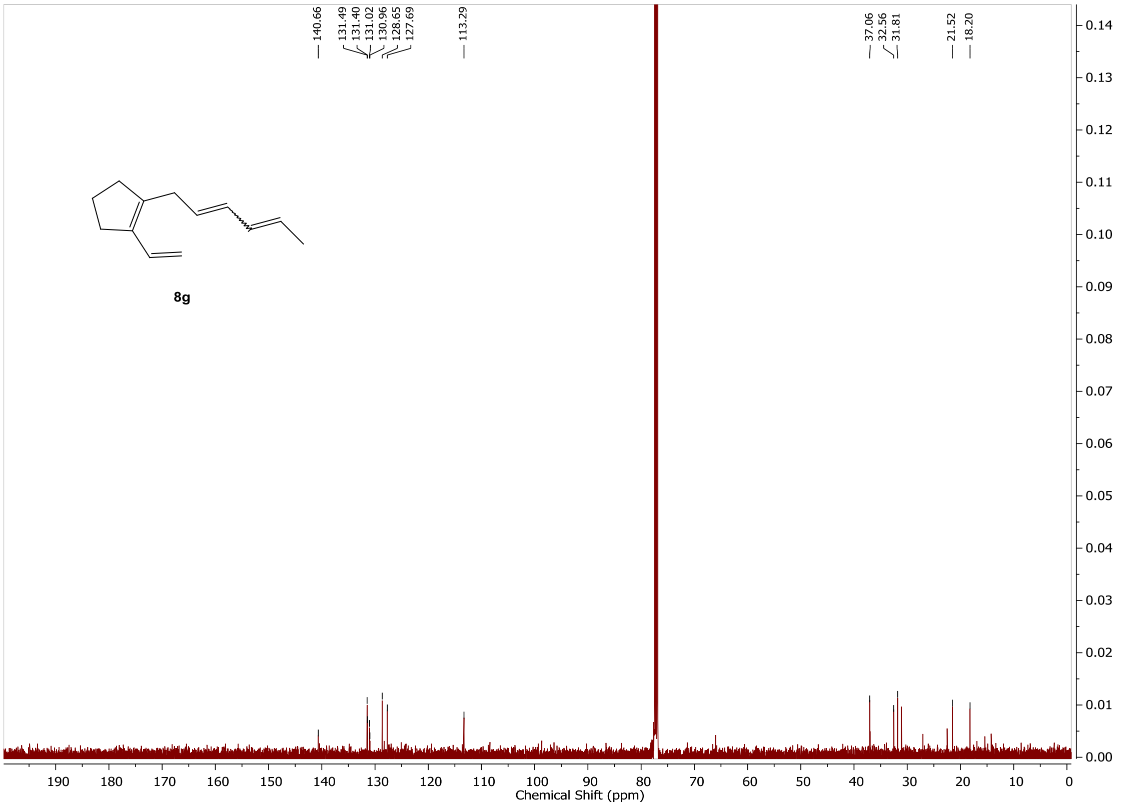
140.66 131.49 131.40 131.02 130.96 128.65 127.69 113.29 37.06 32.56 31.81 21.52 18.20
8g
Chemical Shift (ppm)

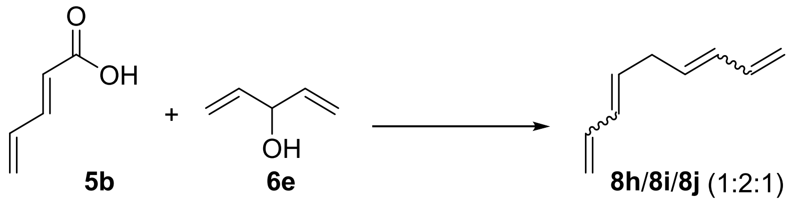

Nona-1,3,6,8-tetraene, 8h/8i/8j

Following the general procedure, tetraenes **8h/8i/8j** were synthesized as a colorless solution in $CDCl_3$ (14%, ^{1}H NMR yield). R_f = 0.73 (hexanes).

Mixture of three inseparable diastereomers (*E*/*E*, *E*/*Z*, *Z*/*Z*)

^{1}H NMR (400 MHz, $CDCl_3$) δ 6.71 – 6.58 (m, 1H), 6.38 – 6.27 (m, 1H), 6.13 – 6.00 (m, 2H), 5.70 (dq, J_d = 15.3 Hz, J_q = 6.4 Hz, 1H), 5.50 – 5.40 (m, 1H), 5.23 (dd, *J* = 16.9, 1.6 Hz, 1H), 5.15 – 5.1 (m, 2H), 4.99 (dd, *J* = 10.0, 3.3 Hz, 1H), 3.09 (t, *J* = 7.6 Hz, 0.53H *diastereomer* **8h**), 2.99 (t, *J* = 7.1 Hz, 0.99H *diastereomer* **8i**), 2.99 (t, *J* = 6.7 Hz, 0.60H *diastereomer* **8j**) ppm.

^{13}C NMR (100 MHz, $CDCl_3$) δ 137.0, 132.5, 132.3, 132.1, 132.0, 131.9, 131.6, 130.2, 129.8, 129.8, 129.8, 129.3, 117.8, 115.7, 115.6, 35.4, 30.7, 26.6 ppm.

GC-LR-MS (EI 70 eV) m/z (%) calcd. for C_9H_{12}: 120, found: 120.

Attempts to obtain HRMS data using ESI and API were unsuccessful.

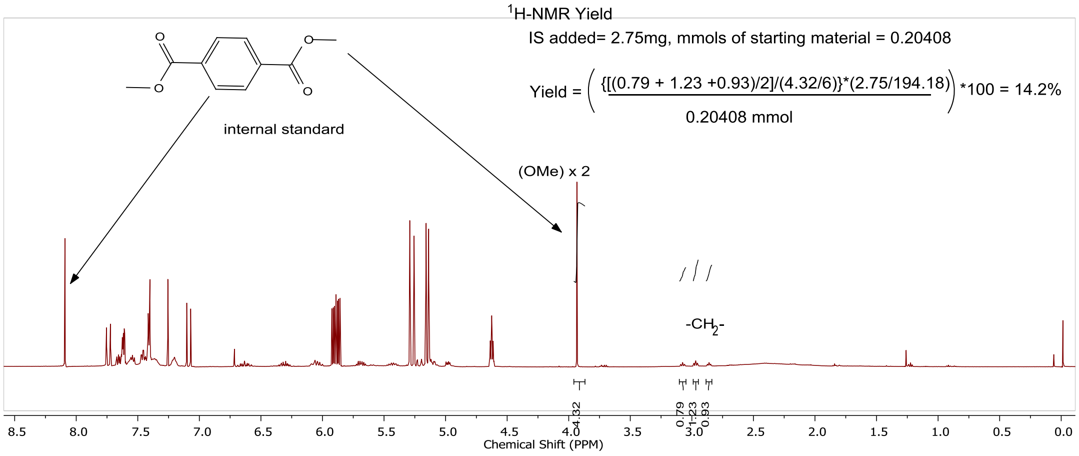

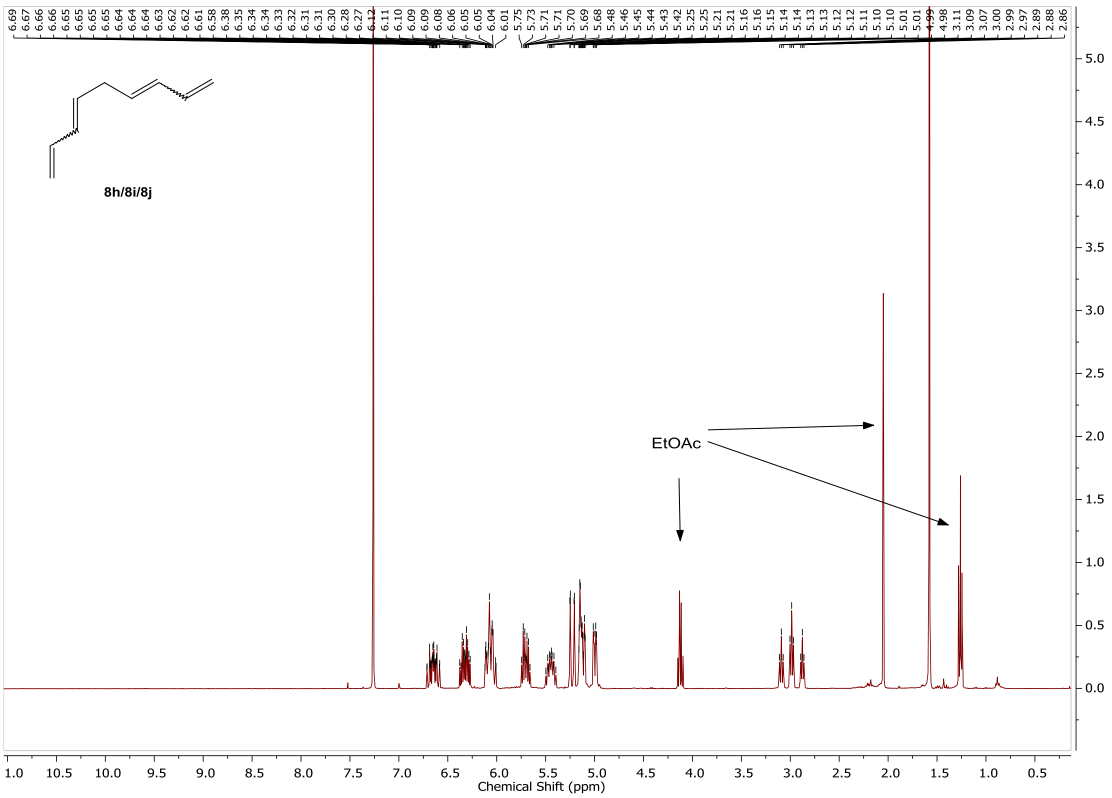
8h/8i/8j
EtOAc
Chemical Shift (ppm)

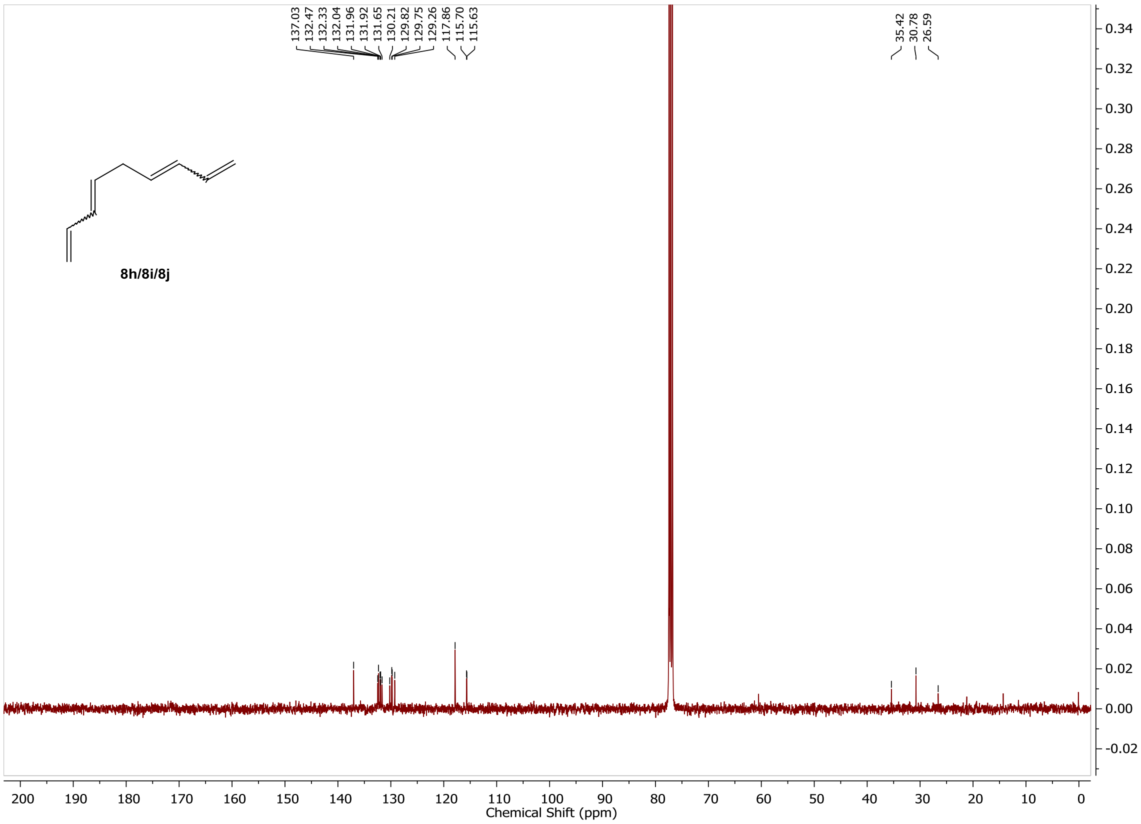
137.03
132.47
132.33
132.04
131.96
131.92
131.65
130.21
129.82
129.75
129.26
117.86
115.70
115.63
35.42
30.78
26.59
8h/8i/8j
Chemical Shift (ppm)

COSY for 8h/8i/8j

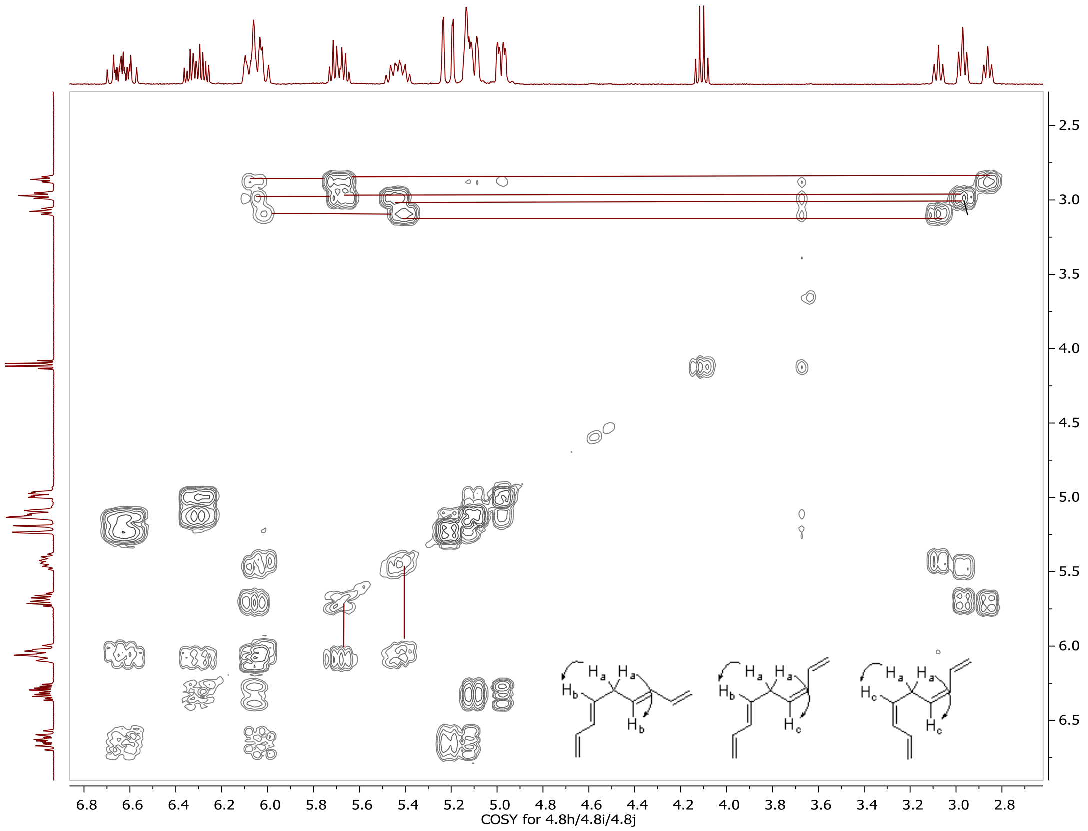

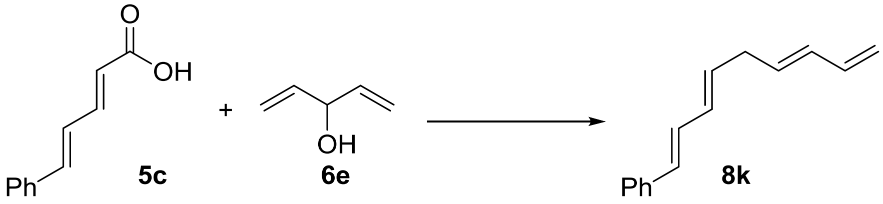

((1*E*,3*E*,6*E*)-Nona-1,3,6,8-tetraen-1-yl)benzene (8k)

Following the general procedure, tetraene **8k** was synthesized as a yellow oil (8.2 mg, 24%, isolated yield). R_f = 0.62 (hexanes).

^{1}H NMR (400 MHz, $CDCl_3$) δ 7.37 – 7.35 (m, 2H), 7.29 (dt, J_d = 8.6 Hz, J_t = 6.8 Hz, 2H), 7.19 (tt, J = 6.4, 1.2 Hz, 1H), 6.75 (dd, J = 15.6, 10.5 Hz, 1H), 6.64 (dtd, J_d = 16.9, 1.0 Hz, J_t = 10.6 Hz, 1H), 6.45 (d, J = 12.0 Hz, 1H), 6.23 (ddt, J_d = 14.0, 10.4 Hz, J_t = 1.7, 1H), 6.09 (t, J = 11 Hz, 1H), 5.80 (dt, J_d = 15.0 Hz, J_t = 6.6 Hz 1H), 5.49 (dt, J_d = 10.7 Hz, J_t = 7.9 Hz, 1H), 5.23 (dd, J = 16.9, 1.6 Hz, 1H), 5.14 (td, J_d = 10.4, J_t = 1.9 Hz, 1H), 3.04 (t, J = 7.1 Hz, 2H) ppm.

^{13}C NMR (100 MHz, $CDCl_3$) δ 137.6, 132.7, 132.0, 131.3, 130.8, 130.3, 129.3, 129.1, 128.6 (2C), 127.3, 126.2 (2C), 117.9, 31.1 ppm.

HRMS (APPI) calcd. for $[C_{15}H_{16} + H]^+$: 197.1330, found: 197.1323.

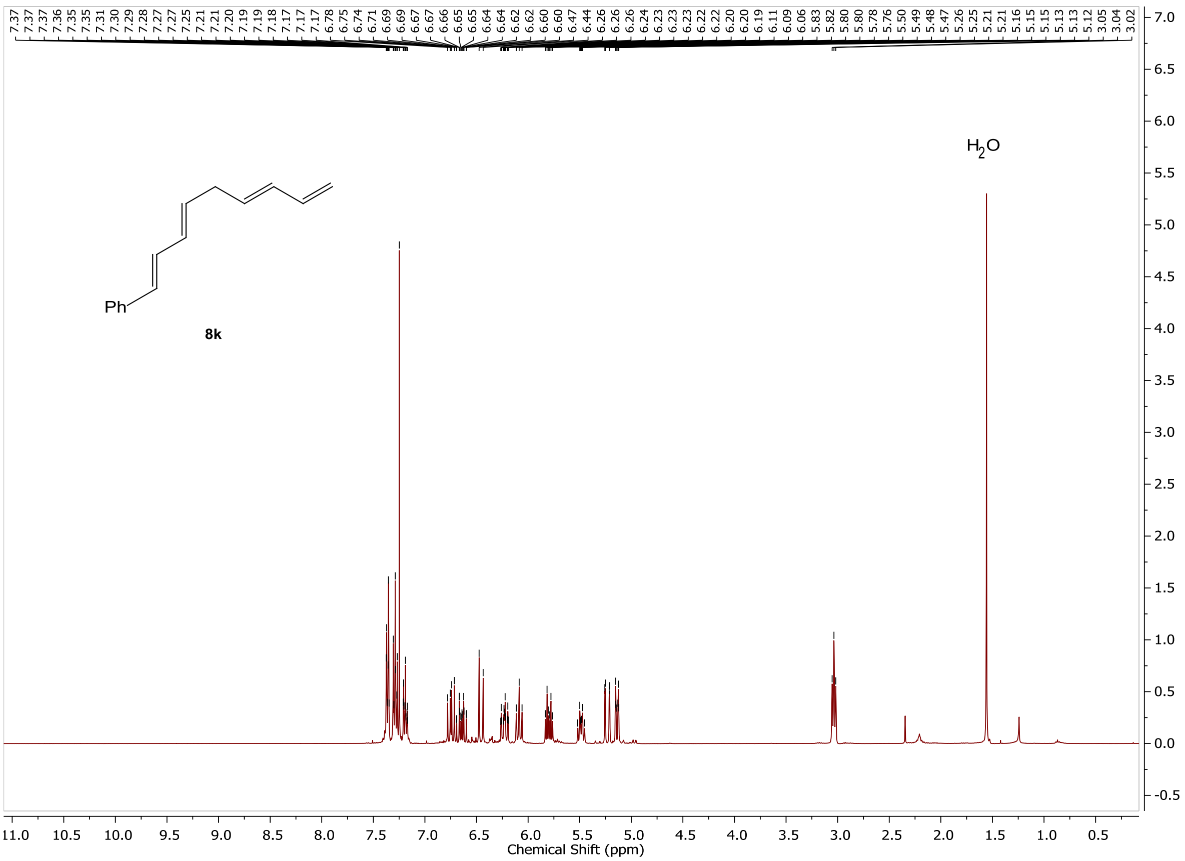

H_2O
Ph
8k
Chemical Shift (ppm)

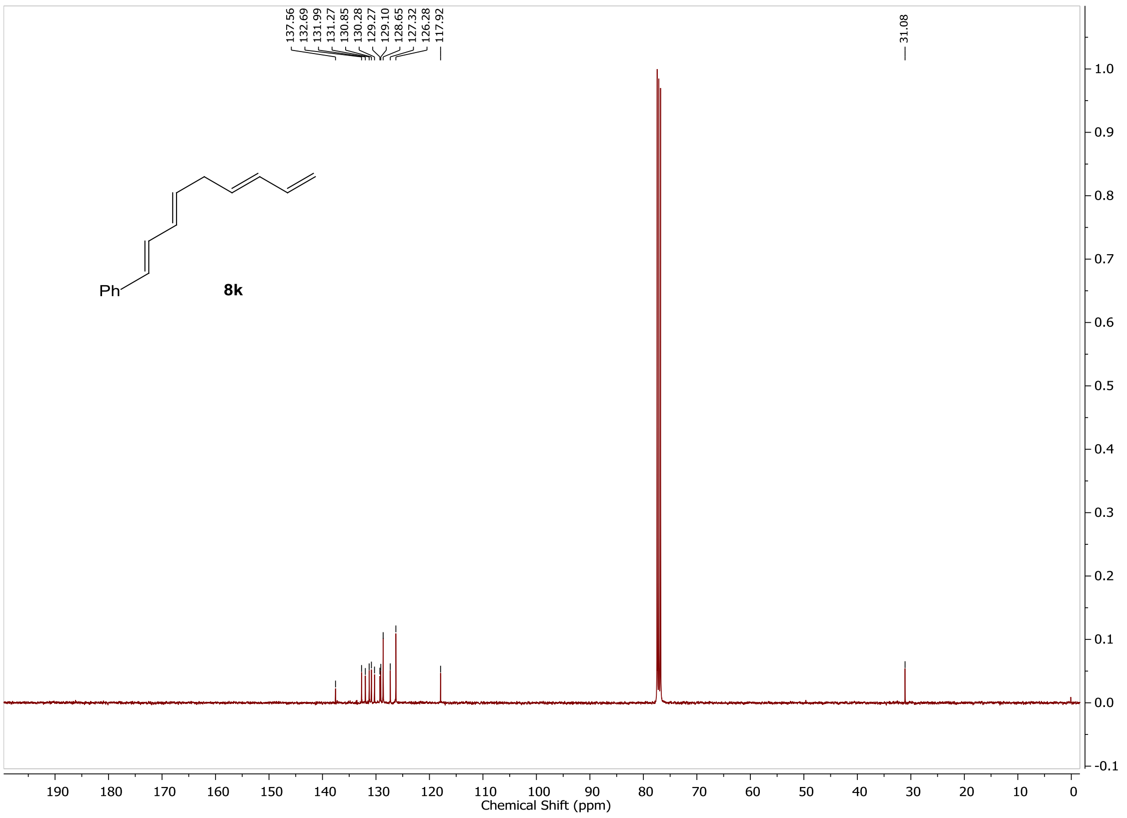

137.56
132.69
131.99
131.27
130.85
130.28
129.27
129.10
128.65
127.32
126.28
117.92
31.08
Ph
8k
Chemical Shift (ppm)

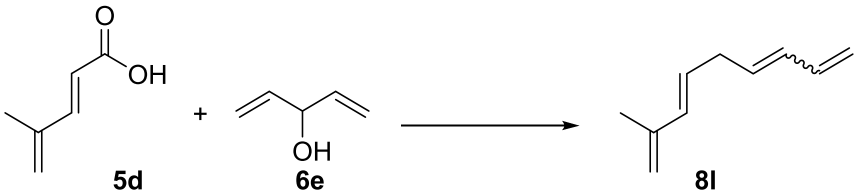

5d 6e 8l

(3*E*,6*E*)-2-Methylnona-1,3,6,8-tetraene (8l)

Following the general procedure, tetraene **8l** was synthesized as a colorless liquid (36% ^{1}H NMR yield). R_f = 0.69 (hexanes). The product is a mixture of two diastereomers in a 4:1 ratio.

Mixture of two inseparable diastereomers

^{1}H NMR (500 MHz, $CDCl_3$) δ 6.65 (dddd, *J* = 16.8, 11.2, 10.2, 1.1 Hz, 1H, *minor isomer*), 6.34 (dtd, J_d = 17.0, 0.7 Hz, J_t = 10.2 Hz, 1H, *major isomer*), 6.21 – 6.12 (m, 1H), 6.12 – 6.01 (m, 1H), 5.73 (dtd, J_d = 15.2, 0.7 Hz, J_t = 6.8 Hz, 1H), 5.68 – 5.58 (m, 1H), 5.52 – 5.42 (m, 1H, *minor isomer*), 5.25 – 5.21 (m, 1H, *minor isomer*), 5.15 – 5.11 (m, 1H), 5.00 (ddd, *J* = 10.3, 1.6, 0.9 Hz, 1H), 4.90 – 4.88 (m, 2H), 3.01 (t, *J* = 7.1 Hz, 2H, *CH_2 minor isomer*), 2.90 (t, *J* = 6.7 Hz, 2H, CH_2 *major isomer*), 1.84 (t, *J* = 1.0 Hz, 3H, CH_3 *major isomer*), 1.83 (t, *J* = 1.0 Hz, 1H, CH_3 *minor isomer*) ppm.

^{13}C NMR (125 MHz, $CDCl_3$) δ 142.1, 137.1, 134.0, 133.6, 132.8, 132.1, 131.9, 130.1, 129.7, 128.2, 128.0, 115.6, 115.2, 115.1, 35.7, 29.8, 18.8 ppm.

GC-LR-MS (EI 70 eV) m/z (%) calcd. for $C_{10}H_{14}$: 134, found: 134.

Attempts to obtain HRMS data using ESI and API were unsuccessful.

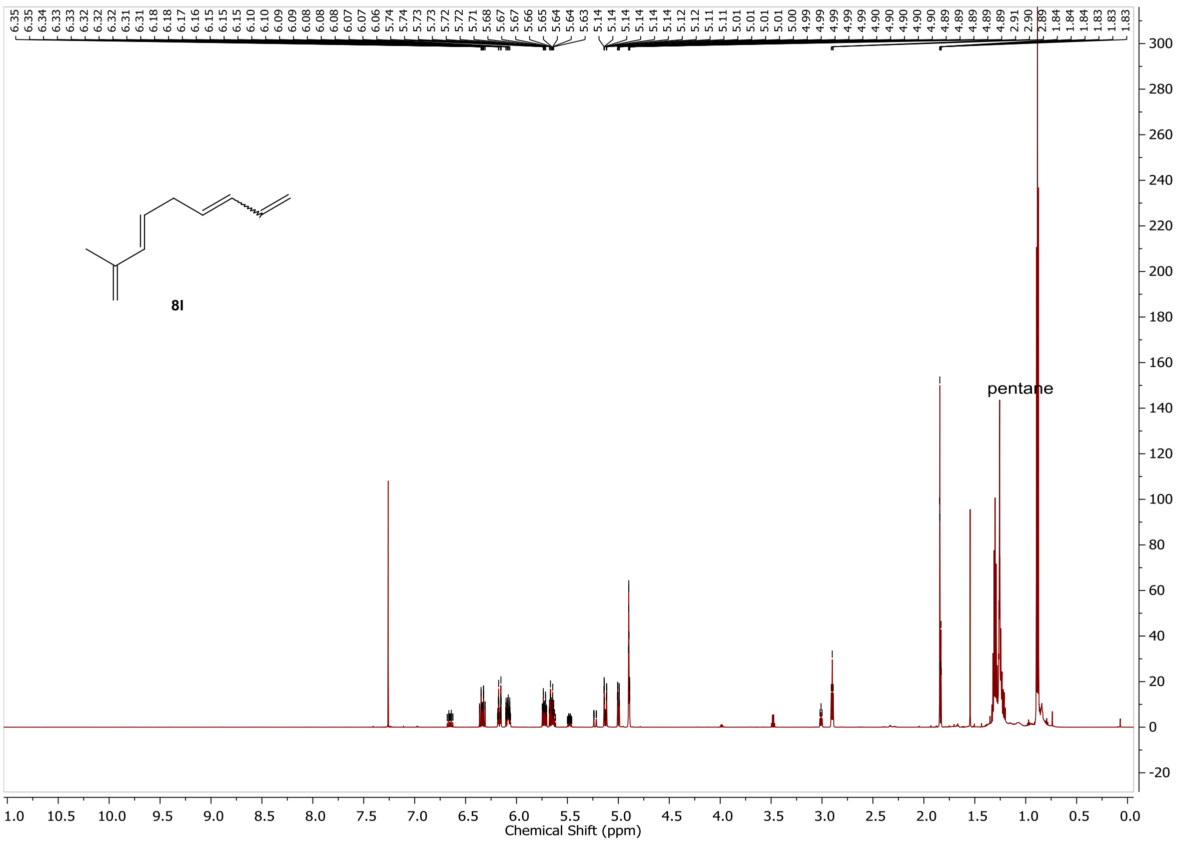
6.35 6.35 6.34 6.33 6.33 6.32 6.32 6.32 6.31 6.31 6.18 6.18 6.17 6.16 6.15 6.15 6.15 6.10 6.10 6.09 6.09 6.08 6.08 6.08 6.07 6.07 6.06 5.74 5.74 5.73 5.73 5.72 5.72 5.71 5.68 5.67 5.67 5.66 5.65 5.65 5.64 5.64 5.63 5.14 5.14 5.14 5.14 5.14 5.14 5.14 5.12 5.12 5.11 5.11 5.01 5.01 5.01 5.01 5.00 4.99 4.99 4.99 4.99 4.99 4.90 4.90 4.90 4.90 4.90 4.89 4.89 4.89 4.89 4.89 2.91 2.90 2.89 1.84 1.84 1.84 1.84 1.83 1.83 1.83
8l
pentane
Chemical Shift (ppm)

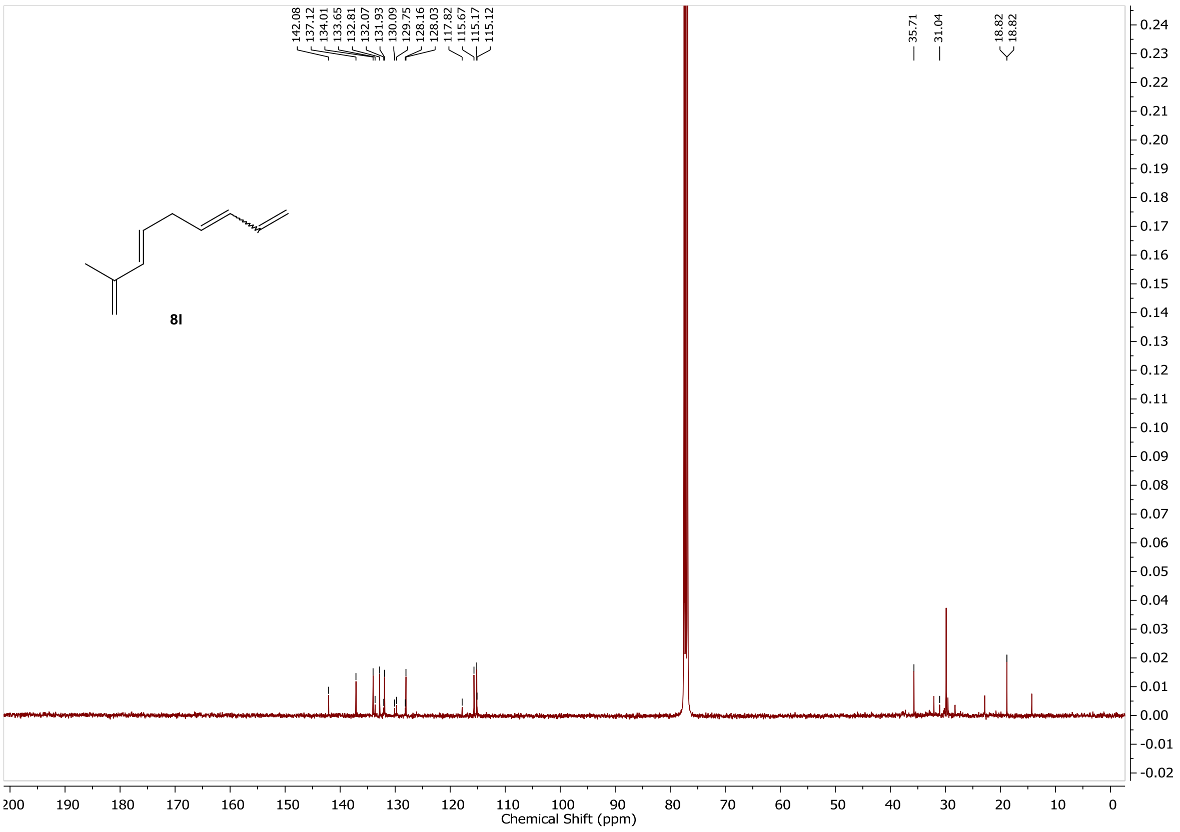
142.08 137.12 134.01 133.65 132.81 132.07 131.93 130.09 129.75 128.16 128.03 117.82 115.67 115.17 115.12
35.71
31.04
18.82 18.82
8l
Chemical Shift (ppm)

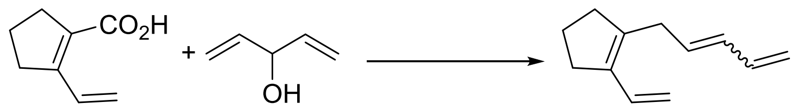

5g **6e** **8m**

(*E*,*Z*)-1(Penta-2,4-dien-1-yl)-2-vinylcyclopent-1-ene (8m)

Following the general procedure, tetraene **8m** was isolated as a colorless oil (15 mg, 74%, ^{1}H NMR yield). R_f (mixture of diastereomers) = 0.91 (hexanes). The product is a mixture of two diastereomers in a 1.85:1 ratio.

Mixture of two inseparable diastereomers

Diastereomer A (major):

^{1}H NMR (500 MHz, $CDCl_3$) δ 6.74 – 6.64 (m, 2H) 6.07 – 6.02 (m, 2H), 5.42 – 5.37 (m, 1H), 5.15 – 5.13 (m, 1H), 5.09 – 5.05 (m, 2H), 3.11 (d, J = 7.7 Hz, 2H), 2.47 – 2.51 (m, 2H), 2.40 – 2.43 (m, 2H), 1.79 – 1.86 (m, 2H) ppm.

Diastereomer B (minor):

^{1}H NMR (500 MHz, $CDCl_3$) δ 6.74 – 6.64 (m, 1H), 6.30 (dt, J_d = 17.0, J_t = 10.3 Hz, 1H), 5.68 - 5.62 (m, 1H), 5.42 – 5.37 (m, 1H), 5.24 – 5.20 (m, 2H), 5.15 – 5.13 (m, 1H), 4.97 – 4.99 (m, 1H), 2.99 (d, J = 7.1 Hz, 2H), 2.47 – 2.51 (m, 2H), 2.40 – 2.43 (m, 2H), 1.79 – 1.86 (m, 2H) ppm.

^{13}C NMR (125 MHz, $CDCl_3$) δ 140.4, 140.1, 137.1, 135.1, 134.7, 132.1, 132.0, 131.9, 130.9, 130.8, 130.0, 129.3, 117.7, 115.5, 113.5, 113.5, 37.1, 36.9, 32.5, 32.5, 31.8, 27.2, 21.5, 21.5. ppm.

HRMS (APPI) calcd. for [$C_{12}H_{16}]^{+}$: 161.1330, found: 161.1323.

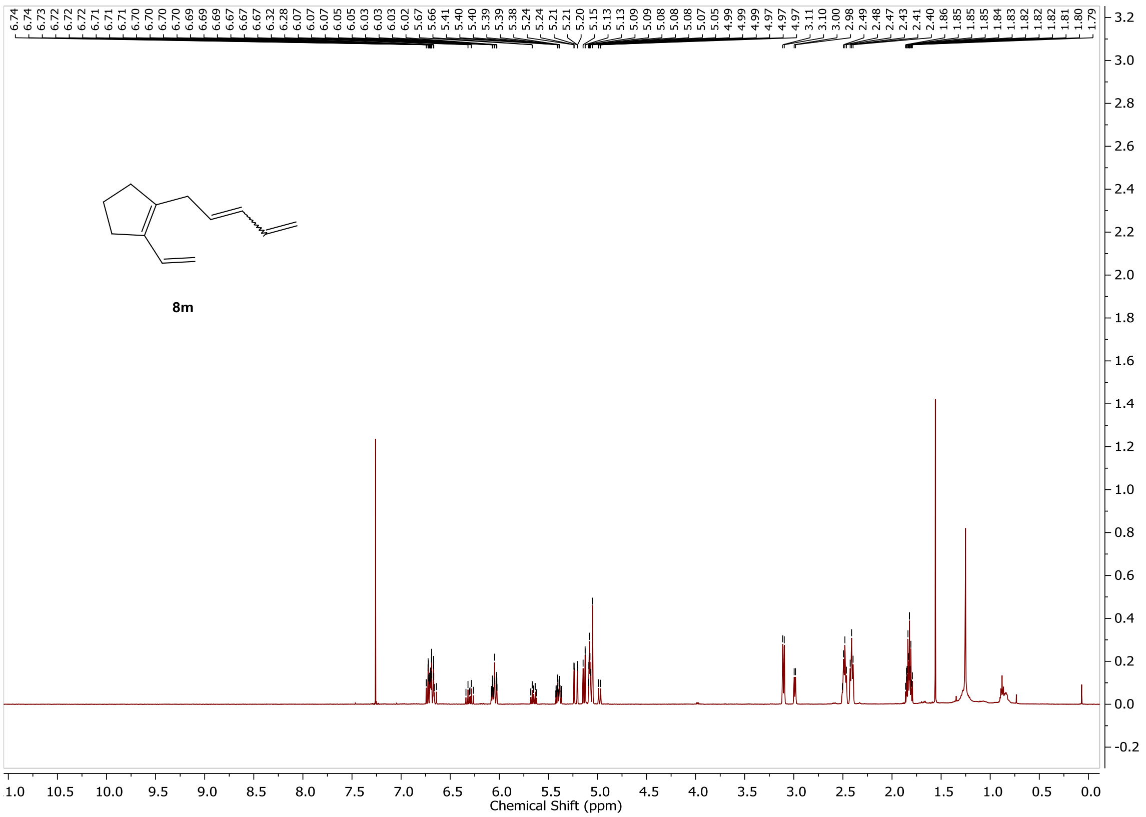
8m
Chemical Shift (ppm)

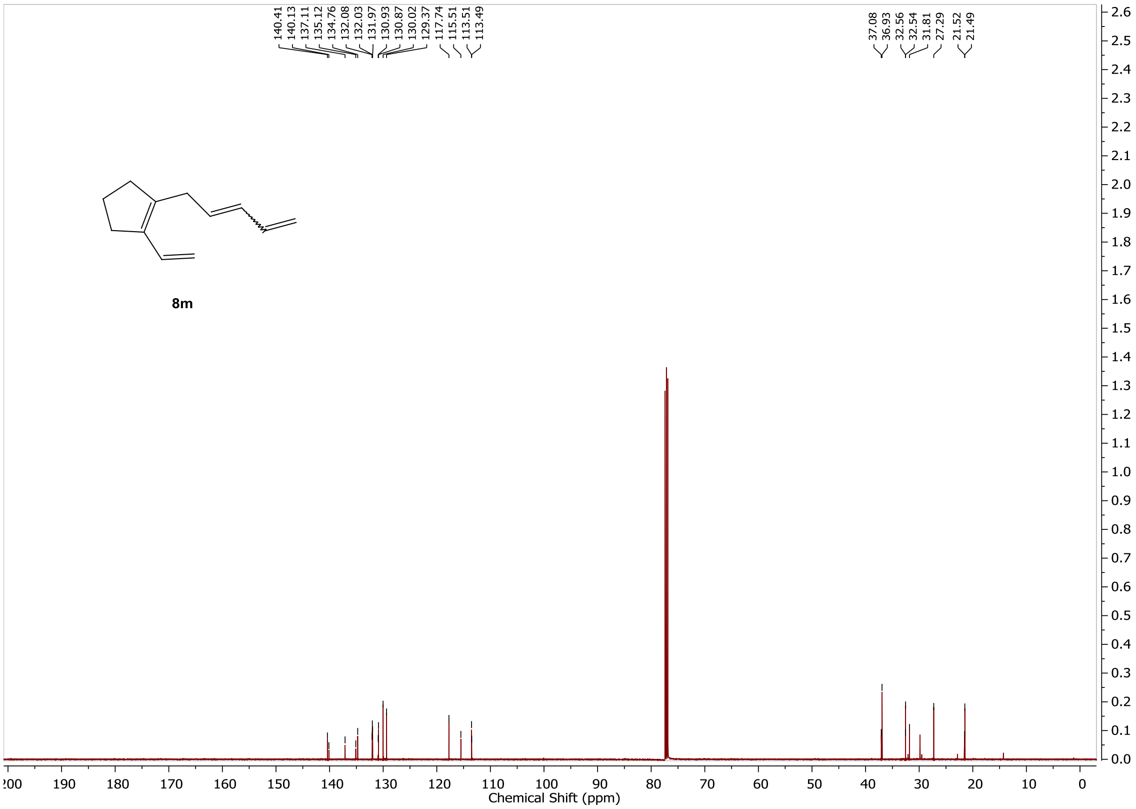
140.41
140.13
137.11
135.12
134.76
132.08
132.03
131.97
130.93
130.87
130.02
129.37
117.74
115.51
113.51
113.49
37.08
36.93
32.56
32.54
31.81
27.29
21.52
21.49
8m
Chemical Shift (ppm)

Product inhibition experiment

Two reactions were run side-by-side to determine if the 1,3,6,8-tetraenes were inhibiting formation of product (see below). The reactions used the same starting dienoic acid, divinylcarbinol, catalyst, ligand, solvent, temperature, and concentration. The only difference was that one reaction had 30 mol % of a previously synthesized tetraene product. In the presence of the tetraene, the yield dropped from 35% to 21% for the side-by-side comparison.

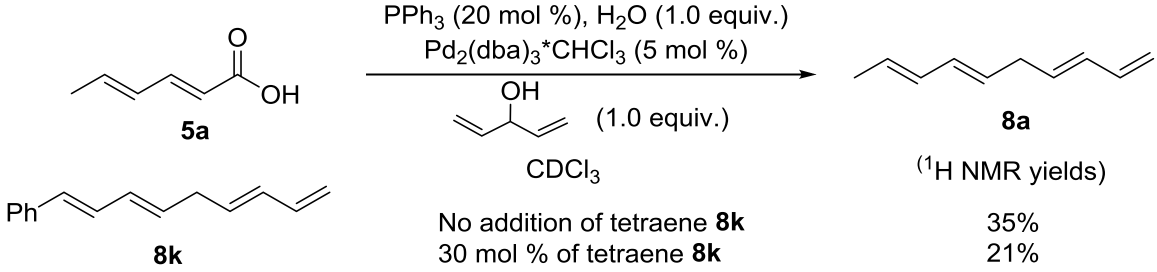

Computational methods

Computed energies, optimized molecular geometries, and harmonic frequencies were calculated with the Gaussian 09 program.[xiii] Two levels of theory were utilized for the structures reported in Scheme 3. For the Baylis–Hillman type mechanism, a 6-311+G** basis was used in order to accommodate the large degree of charge separation in the structures. For the new mechanism we proposed, the more modest 6-31G* basis was employed. The hybrid B3LYP functional was utilized along with a 6-31G* basis and a PCM approximation of water solvation. The nudged elastic band (NEB) method[xiv] was used to map out reaction pathways and locate transition states. NEB is a computational algorithm for determining the reaction energy path (REP) between a reactant and product. A series of interpolated structures, or images, is generated to depict the geometric evolution between the two end points, and the corresponding energies and energy gradients are computed for each image. The total gradient, or force, on each image, is projected into so-called parallel and perpendicular forces, where the former refers to the force along the REP, while the latter is that normal to the REP. Force constants are applied along the parallel direction to prevent the images from collapsing back to the local minima (the reactant or product) and to keep the images evenly spaced. The perpendicular forces are minimized (in mass weighted coordinates with our implementation, to accelerate convergence, which is particularly useful for systems with heavy atoms). In cases where more than one pathway exists between endpoints, NEB distinguishes between the paths and is able to locate the one of lowest energy. While NEB can be run to convergence to obtain the transition state, in practice for this study only a few hundred NEB cycles were run, the last one hundred or so with the climbing image option activated to ensure that the highest of the movable images was as close to the transition state as possible. This image, along with additional data from the NEB calculation, was utilized

in the Modified Dimer program[xv] to locate the transition state. Dimer is a local surface-walking algorithm that calculates only the lowest eigenvalue and eigenvector, rather than the full Hessian. The dimer vector, in our case, is defined by the two NEB images (the estimate of the TS and an adjacent image or point defining the tangent vector) and is rotated to locate the lowest curvature mode. The algorithm then steps in this direction uphill toward the saddle point.

Relevant Cartesian coordinates

Table S1. Cartesian coordinates and DFT/B3LYP/6-311+G** or 6-31G* energies of optimized molecular structures from Scheme 3. The first line of each list of coordinates is the number of atoms, the second line is the title line and total energy (in Hartrees), and the rest are the coordinates (units in Ångstroms).

34
Scheme 3 Structure B E(RB3LYP) = -821.973831292

46	1.093741	1.338656	2.111950
8	-1.023369	0.475422	-0.381876
6	0.423163	0.533845	-2.265819
1	-1.463438	-0.371310	-2.647902
1	-0.463981	-0.196047	-4.079745
1	3.775348	0.472237	-5.450155
1	4.230694	0.751837	-3.678942
6	-0.772770	0.302955	-3.158748
1	1.427092	0.303946	-4.850349
6	3.456937	0.572090	-4.419095
6	2.163524	0.480122	-4.072064
1	2.467698	0.793530	-1.955770
1	2.989527	3.079777	2.346119
6	1.455913	1.824318	4.149024
1	0.954769	1.234921	4.908599
1	1.252052	2.891405	4.168399
1	3.027938	0.356977	3.835842
1	-1.188269	0.698786	1.148893
6	-1.517248	1.603493	-3.513522
1	-2.391166	1.389218	-4.135355
1	-1.861998	2.116148	-2.612309
1	-0.868048	2.288305	-4.066343
6	2.645704	1.338512	3.572003
6	1.699769	0.611833	-2.701747
6	3.147447	2.005881	2.427323
6	4.215363	1.447962	1.603156
1	4.445642	0.395189	1.748714
6	4.904882	2.157139	0.700955
1	5.694114	1.705341	0.111972
1	4.698717	3.208701	0.527492
8	1.121584	1.053316	-0.041151

6 0.150267 0.699615 -0.788331
8 -1.082072 0.955909 2.125205
1 -1.316949 0.190152 2.661258

34
Scheme 3 PPh3 E(RB3LYP) = -1036.50530375 Sum of PPh3 and B is baseline: -1858.479135042
6 3.732119 5.730963 -2.169009
6 3.171509 3.140764 -6.007801
6 -2.319371 5.793793 -1.393592
6 2.929862 3.041411 -4.638878
6 3.312063 4.581241 -0.091112
1 3.533602 5.881450 -3.223494
1 0.956075 5.602503 -6.761643
1 3.475295 2.308720 -4.052473
6 4.698588 6.513937 -1.536095
6 1.281443 4.794662 -4.796216
6 -1.035685 3.857764 -2.058361
6 4.966258 6.339878 -0.178756
6 -2.266878 4.469369 -1.830590
6 1.989969 3.874606 -4.012926
6 1.514206 4.885779 -6.169268
1 -3.276093 6.268482 -1.207116
1 3.904887 2.492157 -6.473903
6 -1.134663 6.500555 -1.191386
15 1.739728 3.641358 -2.190716
1 -1.166939 7.529013 -0.848781
6 3.022506 4.758215 -1.452956
1 4.474942 5.224161 1.598244
1 -3.182861 3.909562 -1.983578
6 2.461100 4.062881 -6.777774
1 5.716852 6.949748 0.311233
1 2.640886 4.134895 -7.844507
1 1.009287 6.455864 -1.272988
6 0.098111 5.891585 -1.431774
1 0.544478 5.443161 -4.337742
6 4.268168 5.371476 0.544060
1 -1.006802 2.821839 -2.381380
6 0.163094 4.564186 -1.873704
1 5.239803 7.261943 -2.105073
1 2.787413 3.819374 0.476821

68
Scheme 3 Structure C E(RB3LYP) = -1858.44645747 Is +20.5 kcal/mol above baseline
46 -0.700875 -5.563515 1.503436
8 -0.358568 -5.335205 -1.829809
6 0.362582 -3.109535 -2.070647
1 0.640475 -4.365323 -3.786629

1	1.331523	-2.764171	-3.948308
1	0.969328	1.438390	-1.798623
1	1.099000	0.499612	-0.315871
6	0.458740	-3.310473	-3.568054
1	1.132439	-0.642332	-3.188114
6	1.420548	0.542481	-1.360312
6	1.086083	-0.702912	-2.106781
1	0.632242	-1.831687	-0.397928
1	-1.498791	-4.063764	3.477133
6	-1.135672	-6.714898	3.218042
1	-0.857347	-7.763104	3.213174
1	-2.187157	-6.514143	3.406617
1	0.871410	-6.045974	3.728230
1	-0.705307	-6.010341	-1.133626
6	-0.784379	-2.853288	-4.356992
1	-0.645289	-2.989322	-5.435497
1	-1.666509	-3.427210	-4.057777
1	-1.000284	-1.795832	-4.178774
6	-0.158528	-5.747348	3.554433
6	0.701100	-1.859554	-1.484042
6	-0.463898	-4.382653	3.367879
6	0.547050	-3.335307	3.466043
1	1.584822	-3.661587	3.452607
6	0.270400	-2.028664	3.569240
1	1.058231	-1.288460	3.642776
1	-0.752275	-1.664502	3.583372
8	-0.262977	-4.039267	0.031025
6	-0.089564	-4.153522	-1.240409
8	-1.280085	-6.862002	-0.051179
1	-0.918592	-7.740378	0.099550
1	3.626233	-1.004357	-3.380744
15	3.245858	0.980271	-1.277489
6	4.161122	-0.282513	-0.345735
6	3.610786	-0.797444	0.836862
1	2.635877	-0.476982	1.182298
6	4.324292	-1.727384	1.587151
1	3.893733	-2.122760	2.499142
6	5.586225	-2.147748	1.166495
1	6.138102	-2.873127	1.752860
6	6.137987	-1.634422	-0.005620
1	7.119326	-1.955734	-0.333756
6	5.430413	-0.703086	-0.762843
1	5.870267	-0.308825	-1.669848
6	3.428342	2.581186	-0.431865
6	2.563528	3.639481	-0.752844
1	1.799740	3.526394	-1.512558
6	2.686964	4.861394	-0.096688
1	2.016559	5.674005	-0.349769

6	3.666596	5.036829	0.880339
1	3.758735	5.988981	1.389655
6	4.526069	3.987534	1.202579
1	5.288085	4.119890	1.961439
6	4.409828	2.761427	0.552070
1	5.079767	1.952229	0.813503
6	3.939447	1.094044	-2.952616
6	4.416754	2.312774	-3.452163
1	4.401600	3.203141	-2.837093
6	4.922219	2.382174	-4.748520
1	5.292737	3.327160	-5.127653
6	4.954899	1.242321	-5.550279
1	5.350826	1.299476	-6.557568
6	4.484988	0.026066	-5.054479
1	4.515224	-0.863242	-5.672733
6	3.979398	-0.054238	-3.759964

68

Scheme 3 Structure D E(RB3LYP) = -1858.44159742 Is +23.6 kcal/mol above baseline

46	0.965854	-7.023109	0.912038
8	-2.392212	-4.772501	-2.397900
6	-2.420531	-2.765765	-1.119380
1	-4.269962	-1.667879	-1.209387
1	-4.361772	-3.253115	-1.954745
1	0.211364	0.980547	-1.685008
1	-0.739130	0.912914	-0.204283
6	-3.734595	-2.363469	-1.863452
1	-0.008449	-1.491082	-2.021288
6	-0.022291	0.359897	-0.815628
6	-0.561348	-0.973933	-1.249739
1	-2.209343	-0.931613	0.018028
1	-2.148535	-6.066849	0.889388
6	-0.298453	-7.170301	2.569123
1	0.271197	-7.402535	3.465161
1	-1.129056	-7.846653	2.374674
1	0.246380	-5.085357	2.600790
1	-1.825998	-5.389468	-2.888109
6	-3.535672	-1.726347	-3.240441
1	-3.059824	-2.418443	-3.941392
1	-4.502269	-1.443921	-3.665359
1	-2.918940	-0.826302	-3.183484
6	-0.348034	-5.844410	2.094267
6	-1.679011	-1.498654	-0.746589
6	-1.456877	-5.325634	1.289659
6	-1.681824	-4.026970	1.026509
1	-0.992862	-3.282509	1.422331
6	-2.828990	-3.516937	0.203371
1	-3.422844	-2.799475	0.782871

1	-3.493853	-4.341033	-0.064135
8	-0.433193	-3.713712	-2.212753
6	-1.616443	-3.755699	-1.962389
8	2.285476	-7.593453	-0.574649
1	3.186539	-7.447912	-0.267530
1	1.708192	-2.494810	-0.726148
15	1.540697	0.301416	0.173178
6	1.213347	-0.466856	1.784973
6	0.074902	-0.087945	2.512572
1	-0.624416	0.639671	2.119428
6	-0.159914	-0.641974	3.766828
1	-1.041120	-0.348778	4.324578
6	0.734986	-1.568854	4.302761
1	0.547286	-1.998619	5.279712
6	1.869526	-1.940898	3.584941
1	2.567919	-2.657828	3.999339
6	2.113005	-1.392733	2.327385
1	2.998528	-1.686847	1.779254
6	2.087617	2.015835	0.427932
6	2.049942	2.933375	-0.632949
1	1.697674	2.641627	-1.614528
6	2.480119	4.241672	-0.431631
1	2.447898	4.946958	-1.253246
6	2.949002	4.640897	0.819902
1	3.281292	5.661046	0.972280
6	2.990039	3.730260	1.874138
1	3.353346	4.037417	2.847432
6	2.560436	2.419134	1.683643
1	2.591822	1.720663	2.510116
6	2.829555	-0.635136	-0.699905
6	4.030105	-0.010751	-1.062177
1	4.203295	1.030544	-0.823828
6	5.013912	-0.735361	-1.731569
1	5.940581	-0.247572	-2.008968
6	4.807146	-2.078549	-2.038390
1	5.574904	-2.639687	-2.558158
6	3.614746	-2.704217	-1.673185
1	3.452764	-3.749701	-1.905926
6	2.624782	-1.990678	-1.004854

68
Image 11 (TS) from bhcc NEB E(RB3LYP) = -1858.37090135 Is +67.9 kcal/mol above baseline

46	-0.026721655	0.306537975	0.291274425
8	-2.629269820	0.742161717	3.045291450
6	-2.947581210	2.984841920	3.739142450
1	-4.110571520	3.444711120	5.444061110
1	-2.758072350	2.442310900	5.873711410

1	-4.322584300	7.333111680	3.280948210
1	-2.573388310	7.117921700	3.267402000
6	-3.527086640	2.585272430	5.093761630
1	-4.697751540	4.890067490	2.876725630
6	-3.509251790	6.750630870	2.838550470
6	-3.703091210	5.278949250	3.071654520
1	-1.766949720	4.879725180	3.700596390
1	1.508402090	1.347667160	2.118755040
6	1.997119560	-0.111998189	-0.042692375
1	2.275335820	-0.586946710	-0.976540007
1	2.340441380	-0.619409947	0.855010399
1	1.872340750	1.894430000	-0.913057482
1	-2.584157940	0.292509396	2.190869080
6	-4.462948130	1.351906390	5.092354530
1	-3.949526800	0.446280438	5.421660160
1	-5.323907660	1.509633480	5.748689170
1	-4.834871110	1.154903080	4.086366840
6	1.824399670	1.299452870	-0.005657602
6	-2.752087560	4.440455540	3.497194230
6	1.356152660	1.875003040	1.180789350
6	1.047034360	3.290502510	1.279772700
1	0.900909329	3.817031530	0.343173685
6	1.004204720	3.965611090	2.434535710
1	0.834059751	5.033791730	2.454290830
1	1.133294140	3.471917380	3.391001740
8	-2.396793970	2.368540950	1.487434780
6	-2.652421330	2.117229990	2.709565220
8	-1.283137040	-1.272229490	-0.055933619
1	-1.536245440	-1.345803290	-0.982631808
1	-4.479942740	4.546852430	0.509662143
15	-3.486022350	7.252141800	1.054887260
6	-2.005920090	6.618364370	0.217549177
6	-0.754599708	6.783654370	0.827866732
1	-0.671011854	7.211500960	1.819529960
6	0.397811536	6.394777700	0.155019449
1	1.362920200	6.513202960	0.632152932
6	0.310401448	5.816257090	-1.111399250
1	1.210338830	5.483698720	-1.615780630
6	-0.931585665	5.642697480	-1.716119740
1	-1.002473630	5.186657230	-2.696094860
6	-2.090684460	6.054225580	-1.061265550
1	-3.048615890	5.936429600	-1.550042430
6	-3.437257690	9.068090840	0.987506030
6	-4.260797200	9.825485300	1.833500460
1	-4.938080370	9.347701010	2.530237180
6	-4.220501170	11.215525700	1.776749910
1	-4.858490210	11.795127300	2.432740320
6	-3.363664470	11.856264900	0.882078697

1	-3.333318080	12.938702900	0.843294617
6	-2.546290980	11.106045700	0.038607681
1	-1.878435410	11.599691900	-0.657079395
6	-2.580151430	9.714772010	0.087248776
1	-1.938111270	9.142357950	-0.569550557
6	-4.964521160	6.638236420	0.197072320
6	-5.875690450	7.537117720	-0.370553265
1	-5.706712700	8.604302270	-0.309342047
6	-7.006890670	7.055967770	-1.026317970
1	-7.707501100	7.755937260	-1.465335080
6	-7.231742820	5.684136350	-1.118843610
1	-8.112775450	5.313187560	-1.629272060
6	-6.320739740	4.786233590	-0.561112455
1	-6.489769300	3.718899470	-0.638353054
6	-5.186165440	5.255057740	0.093874958

65

Scheme 3 Structure A E(RB3LYP) = -1781.58575554 Is +5.4 kcal/mol relative baseline

46	1.307980	1.243169	-0.522035
15	1.284600	3.326376	-1.629884
6	-0.314043	4.213612	-1.414439
6	-1.495137	3.457255	-1.314574
1	-1.456588	2.371059	-1.353062
6	-2.729360	4.090976	-1.167185
1	-3.634503	3.495297	-1.091335
6	-2.798456	5.485815	-1.108727
1	-3.759047	5.978285	-0.987057
6	-1.629792	6.244304	-1.202374
1	-1.677265	7.328600	-1.156105
6	-0.392629	5.613593	-1.355071
1	0.509351	6.212863	-1.424890
6	1.436002	3.042879	-3.443213
6	2.313808	2.039369	-3.890223
1	2.875240	1.450534	-3.168992
6	2.469932	1.793133	-5.254860
1	3.152050	1.015985	-5.587454
6	1.744157	2.538691	-6.187806
1	1.860145	2.342405	-7.249909
6	0.865546	3.533576	-5.752052
1	0.298091	4.114580	-6.473513
6	0.710963	3.787089	-4.387298
1	0.025777	4.562976	-4.061047
6	2.578955	4.562991	-1.212151
6	3.348379	5.197252	-2.199778
1	3.194020	4.963401	-3.247939
6	4.317879	6.137138	-1.840324
1	4.908690	6.620552	-2.613342
6	4.524047	6.454081	-0.496350

1	5.278736	7.184725	-0.218704
6	3.759153	5.825118	0.490318
1	3.916271	6.061972	1.538955
6	2.793530	4.879877	0.141245
1	2.226536	4.394404	0.932983
6	2.879386	1.343111	0.871920
1	2.908494	2.256491	1.463037
1	3.765888	1.092215	0.288912
6	2.006355	0.311743	1.334219
1	1.345691	0.549149	2.169500
6	1.776114	-0.798816	0.518222
1	2.526981	-1.079501	-0.222264
6	0.680528	-1.732367	0.724291
1	-0.029954	-1.491272	1.513650
6	0.519476	-2.849929	-0.006238
1	-0.308049	-3.529776	0.170971
1	1.216542	-3.117348	-0.797165
8	0.292570	2.187088	3.295479
6	1.015771	3.155462	3.668438
8	2.037672	3.609100	3.070489
6	0.631772	3.864329	4.976240
6	1.567008	4.942923	5.471442
1	1.058470	5.612004	6.174985
1	1.876625	5.547712	4.612767
6	2.830149	4.369389	6.141201
1	3.509024	5.172827	6.452253
1	3.363947	3.720463	5.440312
1	2.573545	3.779665	7.029354
6	-0.496749	3.476903	5.614132
1	-1.061108	2.673425	5.143094
6	-1.041145	4.003302	6.856330
1	-0.498595	4.807579	7.351178
6	-2.171782	3.549111	7.425966
1	-2.551155	3.963938	8.355592
1	-2.751125	2.744650	6.976701

65

Scheme 3 Structure E E(RB3LYP) = -1781.59053785 Is +2.4 kcal/mol relative to baseline

46	1.653093	1.178381	-0.342701
15	1.541039	3.295350	-1.397765
6	0.406609	4.463888	-0.547854
6	-0.495691	3.975629	0.409243
1	-0.510906	2.915850	0.646002
6	-1.372535	4.845560	1.062465
1	-2.064529	4.455444	1.803029
6	-1.353589	6.209570	0.766831
1	-2.033008	6.886576	1.276747
6	-0.454755	6.705580	-0.182749

1	-0.434021	7.766909	-0.412687
6	0.423185	5.840697	-0.835802
1	1.123551	6.239713	-1.563295
6	0.960597	3.183632	-3.143565
6	1.595697	2.271574	-4.007702
1	2.418687	1.659723	-3.647906
6	1.182018	2.145298	-5.334199
1	1.688676	1.443723	-5.990421
6	0.114507	2.911258	-5.810815
1	-0.213054	2.807028	-6.841119
6	-0.532470	3.805409	-4.956493
1	-1.366159	4.400286	-5.318337
6	-0.112888	3.945006	-3.630909
1	-0.624923	4.647489	-2.982866
6	3.139204	4.222369	-1.470531
6	3.928554	4.274411	-2.629047
1	3.589156	3.809289	-3.547505
6	5.157224	4.940330	-2.619056
1	5.752853	4.974411	-3.526840
6	5.612555	5.563447	-1.456534
1	6.566989	6.081959	-1.452038
6	4.830177	5.522327	-0.299041
1	5.170282	6.011555	0.609206
6	3.605115	4.856225	-0.303942
1	3.004251	4.849640	0.600153
6	3.101213	1.764084	1.121863
1	2.964915	2.709689	1.635903
1	4.031591	1.671798	0.562195
6	2.480716	0.604240	1.657221
1	1.769835	0.710406	2.475180
6	2.548291	-0.617732	0.979614
1	3.361324	-0.777358	0.270410
6	1.774711	-1.787676	1.364903
1	0.997216	-1.639172	2.113115
6	1.978157	-3.012009	0.848428
1	1.387599	-3.865718	1.165815
1	2.746862	-3.195752	0.101212
8	-2.456835	-1.060041	2.104685
6	-3.124634	-0.208020	1.457466
8	-4.288446	0.206154	1.710653
6	-2.449173	0.413086	0.211707
6	-3.208009	1.491301	-0.519164
1	-2.532541	2.144412	-1.083943
1	-3.718520	2.109319	0.225638
6	-4.268899	0.908551	-1.474163
1	-4.836851	1.711226	-1.959269
1	-4.966640	0.279946	-0.913775
1	-3.804019	0.298854	-2.257813

6	-1.249035	-0.084569	-0.174333
1	-0.855651	-0.902887	0.423889
6	-0.457193	0.332870	-1.315332
1	-0.816403	1.168965	-1.910903
6	0.652890	-0.347709	-1.766844
1	1.092656	-0.105393	-2.728695
1	0.931158	-1.299509	-1.322216

65

Scheme 3 Structure F E(RB3LYP) = -1781.59145294 Is +1.8 kcal/mol relative to baseline

46	1.782770	1.075669	-0.894901
15	1.618014	3.269812	-1.651548
6	0.283626	4.200329	-0.789669
6	-0.668761	3.508353	-0.025412
1	-0.602818	2.429067	0.071514
6	-1.692644	4.199145	0.627199
1	-2.419870	3.649753	1.217890
6	-1.773964	5.588895	0.526103
1	-2.568399	6.126738	1.035521
6	-0.824525	6.289082	-0.224052
1	-0.877897	7.371305	-0.299191
6	0.200778	5.602377	-0.874599
1	0.940886	6.160760	-1.439438
6	1.259549	3.374572	-3.457155
6	1.946120	2.500985	-4.319327
1	2.645774	1.775583	-3.911678
6	1.731538	2.551456	-5.697205
1	2.272955	1.874087	-6.351215
6	0.813867	3.461269	-6.229554
1	0.639116	3.494422	-7.301119
6	0.116111	4.320740	-5.379099
1	-0.604878	5.024412	-5.785323
6	0.337900	4.282021	-4.000139
1	-0.214305	4.955770	-3.354546
6	3.101405	4.341990	-1.411290
6	3.992461	4.620092	-2.458449
1	3.797026	4.247265	-3.458253
6	5.135442	5.390303	-2.227226
1	5.812372	5.602230	-3.049962
6	5.402437	5.889960	-0.951820
1	6.290047	6.490673	-0.775409
6	4.520739	5.615782	0.098137
1	4.718424	6.003177	1.093511
6	3.380360	4.845328	-0.127432
1	2.701878	4.646153	0.697269
6	3.920301	1.341259	0.143581
1	4.024136	2.350421	0.523313
1	4.524024	1.081492	-0.723573

6	3.285134	0.373280	0.878238
1	2.816439	0.645300	1.823718
6	3.177624	-1.007788	0.452549
1	3.669833	-1.256975	-0.488931
6	2.409175	-1.947882	1.047989
1	1.915540	-1.716201	1.991334
6	1.986804	-3.155497	0.306994
1	1.851424	-4.033704	0.946279
1	2.700496	-3.399818	-0.484746
8	1.096506	-4.275299	-2.385667
6	0.470217	-4.301052	-1.300968
8	-0.154468	-5.245632	-0.770047
6	0.541501	-2.945716	-0.414909
6	-0.557684	-2.885095	0.666728
1	-0.415169	-1.991108	1.289277
1	-0.412695	-3.746656	1.325883
6	-2.002372	-2.919981	0.146553
1	-2.708259	-2.898761	0.985742
1	-2.171361	-3.838313	-0.421094
1	-2.236305	-2.069130	-0.502063
6	0.597250	-1.784194	-1.322323
1	1.203630	-1.942362	-2.212716
6	-0.026958	-0.566154	-1.167966
1	-0.677093	-0.408971	-0.310297
6	0.142079	0.542274	-2.075235
1	-0.647931	1.290328	-2.100994
1	0.558562	0.325631	-3.060247

65

Scheme 3 Structure E-F TS E(RB3LYP) = -1781.58296413 Is +7.2 higher than baseline

46	-0.024890	0.131336	-0.023887
15	2.341082	0.070201	-0.012123
6	3.043311	-0.317043	-1.667812
6	2.210315	-0.845302	-2.665670
1	1.154794	-0.997679	-2.463357
6	2.724018	-1.164463	-3.924563
1	2.065760	-1.570849	-4.686813
6	4.075823	-0.954200	-4.201661
1	4.475840	-1.199194	-5.181336
6	4.913128	-0.419707	-3.217927
1	5.964282	-0.247607	-3.430269
6	4.402655	-0.099639	-1.959712
1	5.062601	0.326375	-1.210428
6	3.022330	-1.189754	1.150939
6	2.449311	-1.291514	2.431628
1	1.611566	-0.654717	2.704676
6	2.940337	-2.213944	3.356009
1	2.492277	-2.276832	4.343325

6	3.995110	-3.061951	3.006633
1	4.371462	-3.786849	3.722709
6	4.556218	-2.981094	1.731089
1	5.369826	-3.643379	1.449207
6	4.076315	-2.049402	0.807016
1	4.523674	-2.001373	-0.179504
6	3.198960	1.637522	0.461855
6	3.691854	1.847453	1.758580
1	3.635689	1.059438	2.501608
6	4.273823	3.069876	2.105046
1	4.654890	3.213260	3.112170
6	4.372301	4.095889	1.164354
1	4.826354	5.044644	1.435676
6	3.887201	3.894605	-0.130930
1	3.963483	4.684531	-0.872445
6	3.303155	2.676981	-0.480121
1	2.941033	2.535027	-1.494254
6	-0.066916	2.242425	0.025378
1	0.640955	2.763001	-0.610485
1	0.030481	2.463716	1.088750
6	-1.348884	1.922281	-0.503228
1	-1.529842	2.045935	-1.569914
6	-2.322311	1.294648	0.268642
1	-2.192638	1.264512	1.350587
6	-3.597994	0.866923	-0.258260
1	-3.732431	0.930632	-1.336750
6	-4.603581	0.419912	0.514366
1	-5.550457	0.105723	0.091036
1	-4.497799	0.340691	1.593880
8	-5.185550	-2.752141	-0.088846
6	-4.852904	-2.693845	-1.302896
8	-5.610959	-2.771801	-2.308848
6	-3.348425	-2.504653	-1.613881
6	-2.927250	-2.642437	-3.054456
1	-1.972894	-2.137520	-3.244311
1	-3.689332	-2.153756	-3.668988
6	-2.820072	-4.116564	-3.492223
1	-2.549331	-4.188992	-4.552077
1	-3.778811	-4.624056	-3.347592
1	-2.057655	-4.646733	-2.909334
6	-2.496395	-2.269875	-0.584578
1	-2.938867	-2.209063	0.406192
6	-1.055965	-2.199583	-0.670731
1	-0.603018	-2.326679	-1.652046
6	-0.208682	-2.155798	0.414810
1	0.825541	-2.459404	0.308173
1	-0.612196	-2.177831	1.426246

References:

[i] Marcus, A. P.; Lee, A. S.; Davis, R. L.; Tantillo, D. J.; Sarpong, R. *Angew. Chem. Int. Ed.* **2008**, *47*, 6379.

[ii] Song, B.; Knauber, T.; Gooßen, L. J. *Angew. Chem. Int. Ed.* **2013**, *52*, 2954.

[iii] Luo, Y.; Herndon, J. W.; Cervantes-Leeb, F. *J. Am. Chem. Soc.* **2003**, *125*, 12720.

[iv] Hettrick, C. M.; Kling, J. K.; Scott, W. J. *J. Org. Chem.* **1991**, *56* (4), 1489.

[v] Nilsson, Y. I. M; Andersson, P. G.; Backval, J. E. *J. Am. Chem. Soc.* **1993**, *115*, 6609.

[vi] Multu, H.; Ruiz, J.; Solleder, S. C.; Meier, M. A. R *Green Chem.* **2012**, *14*, 1728.

[vii] Irene Bosque, I.; Bagdatli, E.; Foubelo, F.; Gonzalez-Gomez, J. C. *J. Org. Chem.* **2014**, *79*, 1796.

[viii] Graza, V. J.; Krische, M. J. *J. Am. Chem. Soc.* **2016**, *138*, 3655.

[ix] Tabuchi, T.; Inanaga, J.; Yamaguchi, M. *Tetrahedron Lett.* **1986**, *27*, 601.

[x] Graden, H.; Hallberg, J.; Kann, N. *J. Comb. Chem.* **2004**, *6*, 783.

[xi] Harding, K. E.; Strickland, J. B.; Pommerville, J. *J. Org. Chem.* **1988**, *53*, 4877.

[xii] Nagy, E. E.; Hyatt, I. F. D.; Gettys, K. E.; Yeazell, S. T.; Frempong, S. K.; Croatt, M. P. *Org. Lett.* **2013**, *15*, 586.

[xiii] (a) Gaussian 09, Revision D.01, Frisch, M. J.; Trucks, G. W.; Schlegel, H. B.; Scuseria, G. E.; Robb, M. A.; Cheeseman, J. R.; Scalmani, G.; Barone, V.; Mennucci, B.; Petersson, G. A.; Nakatsuji, H.; Caricato, M.; Li, X.; Hratchian, H. P.; Izmaylov, A. F.; Bloino, J.; Zheng, G.; Sonnenberg, J. L.; Hada, M.; Ehara, M.; Toyota, K.; Fukuda, R.; Hasegawa, J.; Ishida, M.; Nakajima, T.; Honda, Y.; Kitao, O.; Nakai, H.; Vreven, T.; Montgomery, J. A., Jr.; Peralta, J. E.; Ogliaro, F.; Bearpark, M. J.; Heyd, J. J.; Brothers, E. N.; Kudin, K. N.; Staroverov, V. N.; Keith, T. A.; Kobayashi, R.; Normand, J.; Raghavachari, K.; Rendell, A. P.; Burant, J. C.; Iyengar, S. S.; Tomasi, J.; Cossi, M.; Rega, N.; Millam, J. M.; Klene, M.; Knox, J. E.; Cross, J. B.; Bakken, V.; Adamo, C.; Jaramillo, J.; Gomperts, R.; Stratmann, R. E.; Yazyev, O.;Austin, A. J.; Cammi, R.; Pomelli, C.; Ochterski, J. W.; Martin, R. L.; Morokuma, K.; Zakrzewski, V. G.; Voth, A.; Salvador, P.; Dannenberg, J. J.; Dapprich, S.; Daniels, A. D.; Farkas, O.; Foresman, J. B.; Ortiz, J. V.; Cioslowski, J.; Fox, D. J. Gaussian, Inc., Wallingford CT, 2013; (b) in all cases, the def2-tzvp basis for Pd was employed: Weigend, F.; Ahlrichs, R. *Phys. Chem. Chem. Phys.* **2005**, *7*, 3297-3305; (c) Andrae, D.; Haeussermann, U.; Dolg, M.; Stoll, H.; Preuss, H. *Theor. Chim. Acta* **1990**, *77*, 123-141.

[xiv] Alfonso, D. R.; Jordan, K. D. *J. Comput. Chem.* **2003**, *24*, 990-996.

[xv] (a) Henkelman, G.; Jónsson, J. *J. Chem. Phys.* **1999**, *111*, 7010-7022.; Heyden, A.; (b) Bell, A.T.; Keil, F.J. *J. Chem. Phys.* **2005**, *123*, 224101-14.
